# Identification of Differentially Expressed Genes Particularly Associated with Immunity in Uremia Patients by Bioinformatic Analysis

**DOI:** 10.1155/2022/5437560

**Published:** 2022-12-29

**Authors:** Guixia Li, Shijun Wang, Jing Huo

**Affiliations:** ^1^Department of Basic Medicine, Changzhi Medical College, Changzhi 046000, China; ^2^Department of Chemistry, Changzhi University, Changzhi 046000, China

## Abstract

Uremia is a common syndrome that happens to nearly all end-stage kidney diseases, which profound have changes in human gene expressions, but the related pathways are poorly understood. Gene Ontology categories and Kyoto Encyclopedia of Genes and Genomes pathways enriched in the differentially expressed genes (DEGs) were analyzed by using clusterProfiler, org.Hs.eg.db, and Pathview, and protein–protein interaction (PPI) network was built by Cytoscape. We identified 3432 DEGs (including 3368 down- and 64 up-regulated genes), of which there were 52 different molecular functions, and 178 genes were identified as immune genes controlled by the four transcription factors (POU domain class 6 transcription factor 1 (POU6F1), interferon regulator factor 7 [IRF7], forkhead box D3 (FOXD3), and interferon-stimulated response element [ISRE]). In the gender research, no significant difference was observed. The top 15 proteins of 178 immune-related genes were identified with the highest degree in PPI network. The DEG analysis of uremia patients was expected to provide fundamental information to relieve pain and add years to their life.

## 1. Introduction

More than two million people around the world have been subjected to chronic kidney disease (CKD) [1]. Uremia is a common syndrome that occurs in nearly all end-stage kidney diseases followed by a series of clinical manifestations, such as metabolic dysregulation and multiple organ disturbance [2]. The etiology of uremia is very complicated, mainly resulting from primary and secondary glomeruli, nephritis, chronic pyelonephritis, and so on [3], which are associated with severe alterations of the immune system, and its molecular mechanism is too hard to figure out in comparison to other single diseases [4]. Several studies in the literature have investigated the concentration changes of individual uremic retention solutes [5, 6]. Multiple causes associated with end-stage renal diseases (ESRD) were highly heterogeneous [7]. Some hereditary nephropathies—including autosomal dominant polycystic kidney disease, Fabry disease, and Alport syndrome—can progress to ESRD [8]. It was reported that less than 10% of adult ESRD was thought to be genetic, mainly attributed to clinically diagnosed autosomal dominant polycystic kidney disease [9]. However, the differences in gene expression in patients with uremia are not fully understood. Teng et al. performed integrated bioinformatics analysis to identify the differentially expressed genes (DEG) and hub genes for the function and pathways in the occurrence and development of calcific aortic valve disease [10], which provided a new idea for the treatment of uremia.

Clinical and experimental data indicated gender difference existed in renal anatomy, physiology, and susceptibility to renal diseases [11]. Sexual dimorphism in renal injury has been acknowledged since the 1940s, and the possible role of sex hormones has been intensively discussed in the last 50 years [12]. It is even more complicated that the disease progression, resultant complications, and overall mortality are heterogeneous by sex in CKD. For example, it appears that men showed a faster decline in kidney function and higher mortality before dialysis, but the death rate is relatively equal between men and women in kidney failure or after dialysis [13].

With the increase in ESRD cases, the economic burden and pain of dialysis are always observed in uremic patients, as well as their caregivers and payers. In this work, we investigated several aspects to provide a close view of uremia, which would lay the foundation for clinical interventions.

## 2. Materials and Methods

### 2.1. Data Resources and Reprocessing

The original dataset GSE37171 was downloaded from the Gene Expression Omnibus (GEO) expression database (http://www.ncbi.nlm.nih.gov/geo), which consisted of 75 uremia samples and 20 normal samples. Raw data were processed by rectangular approximation method from the Affy and affPLM packages of the R program, and Perl was used to convert Probe ID into gene symbols, and limma packages for differential expression analysis of genes. The significance analysis of microarrays algorithm | Log_2_(fold change) | = 1, adjust *P* < 0.05 was used to screen DEGs between uremia patients and control subjects. The heatmap was produced by the gplots package.

### 2.2. The Analysis of GO and KEGG in the DEGs

To explore molecular functions of DEGs, Gene Ontology (GO) analysis was used to annotate and illustrate genes. Kyoto Encyclopedia of Genes and Genomes (KEGG) is a comprehensive database, which contains the information of high-level functions and biological systems from large-scale molecular dataset [14]. Software packages, including clusterProfiler, org.Hs.eg.db, and Pathview, were used in GO and KEGG enrichment analyses.

### 2.3. The Character of Immune Genes in Uremia

Many diseases are associated with immune abnormalities, but the relationship between immunity and uremia has not been explored [15, 16]. More than 3000 immune-related genes were obtained from the Immunology Database and Analysis Portal (https://immport.niaid.nih.gov). CIBERSORT (https://cibersort.stanford.edu/) and ESTIMATE were utilized to discover the composition of 22 kinds of immune cells. HiPlot was used to show significantly different immune cells.

### 2.4. The Regulatory Network of Transcription Factor

In order to investigate the regulatory network of transcription factor (TF) about immune-related genes, DAVID (v6.8) was utilized by UCSC_TFBS, which *Homo sapiens* was considered as the background database, and Benjamini was less than or equal to 0.05 as the screening standard. Cytoscape software (v3.7.0) was taken to map the regulatory networks of TF.

### 2.5. Protein–Protein Interaction Network

String was used to select the interacting proteins, and Cytoscape (v3.7.0) was used to map the PPI regulatory network. The thickness of the line represented the combined score, and the color gradient from blue to red represented enhanced reliability. The size and color of the circle displayed the degree to which the protein could interact with it. The larger and redder circle illustrated that more proteins could be linked to it.

### 2.6. Role of Gender in Uremia

Numerous studies have shown that gender has diverse expression patterns in the same disease, such as cardiovascular disease [17]. To understand the role of gender in uremia, we screened for differential genes by comparing healthy male population with male patients and healthy female population with female patients, as well as healthy male population with healthy female population and male patients with female patients, respectively. KEGG analyzed the signaling pathways involved in various differential genes.

## 3. Results

### 3.1. Differentially Expressed Genes

**Figure 1:**
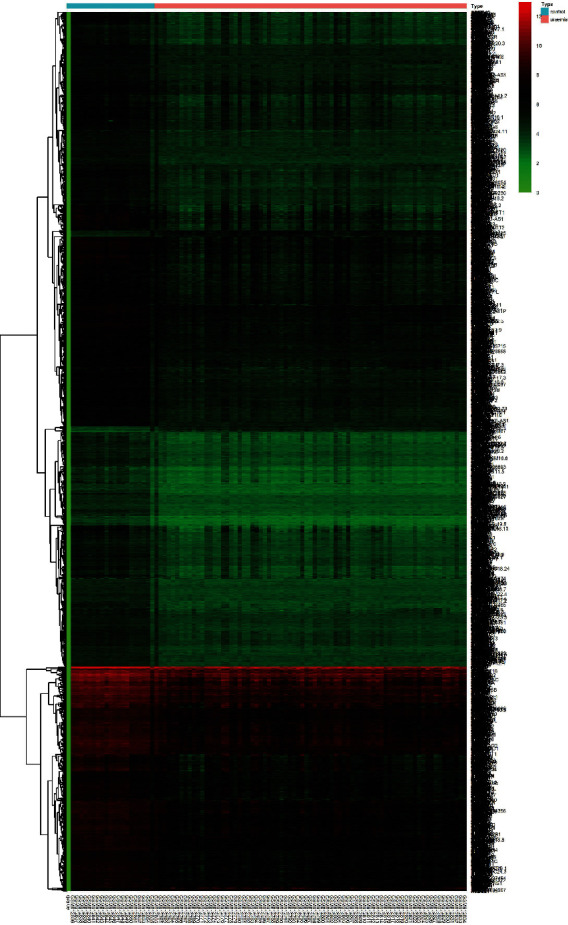
Heatmap of the top 3432 significant DEGs. Red and green indicate up-regulated and down-regulated gene expression.

**Figure 2:**
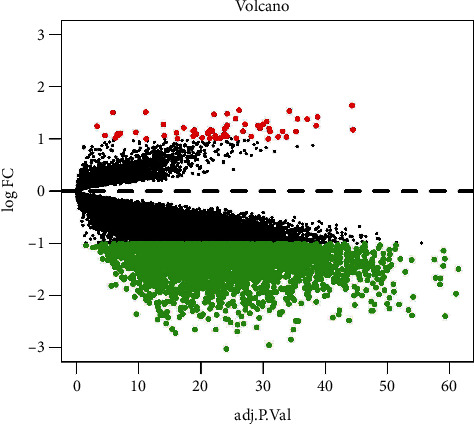
The volcano plot shows the DEGs. The red dots represent up-regulated genes, the green dots represent down-regulated genes, but the black dots represent genes that are non-significantly differentially expressed.

The GSE37171 dataset was standardized to obtain 21,629 genes. Using limma analysis, 3432 DEGs were investigated, of which 3368 genes were down-regulated and 64 genes were up-regulated (Figure 1). The DEGs were represented by a volcano plot, of which the down-regulated genes accounted for the majority, and the down-regulated genes with significant differences marked as green were much more than the up-regulated genes (Figure 2). The heatmap explained the difference in gene expression between normal and diseased individuals. The clustering of DEGs demonstrated that they could be divided into at least four categories. It was indicated that the messenger ribonucleic acid (mRNA) expression profile of the uremia patients was different from that of healthy individuals.

### 3.2. GO and KEGG Analyses

**Figure 3:**
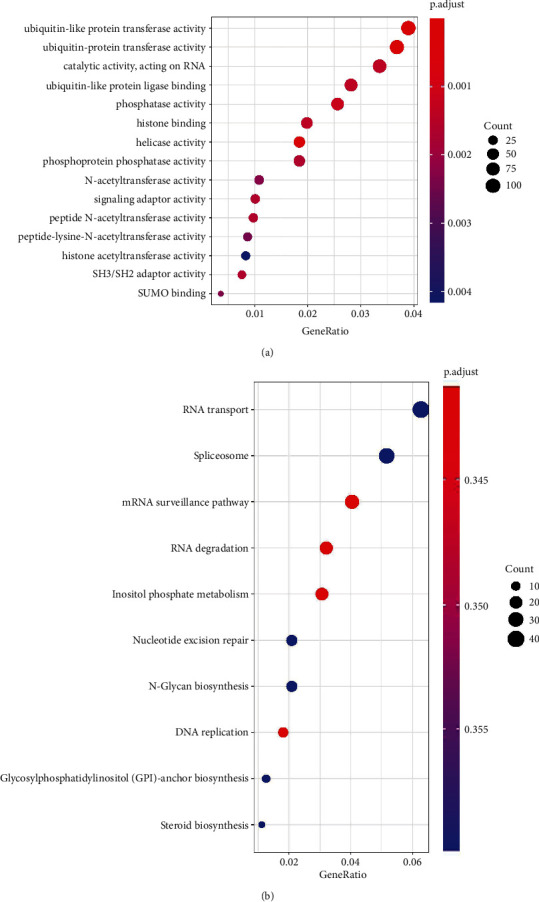
GO and KEGG pathway enrichment analyses of DEGs. (a) Top 15 GO enrichment analysis. (b) Top 10 pathways enrichment analysis.

Go enrichment analysis of DEGs showed that there were 52 different molecular functions, biological processes, and cell components. GO enrichment results of top 15 biological roles had been shown in Figure 3(a). We could observe that the enrichment mainly included ubiquitin-like protein transfer activity, ubiquitin protein transfer activity, helicase activity, phosphatase activity, ubiquitin like protein binding, histone binding, catalytic activity, and acting on RNA. KEGG analysis of 3432 DEGs revealed that there were 10 pathways filtered by adjusted *P* value (less than 0.05), such as RNA transport, spliceosome, mRNA surveillance pathway, RNA degradation, and so on (Figure 3(b)).

### 3.3. The Role of Immune Genes in Uremia

**Figure 4:**
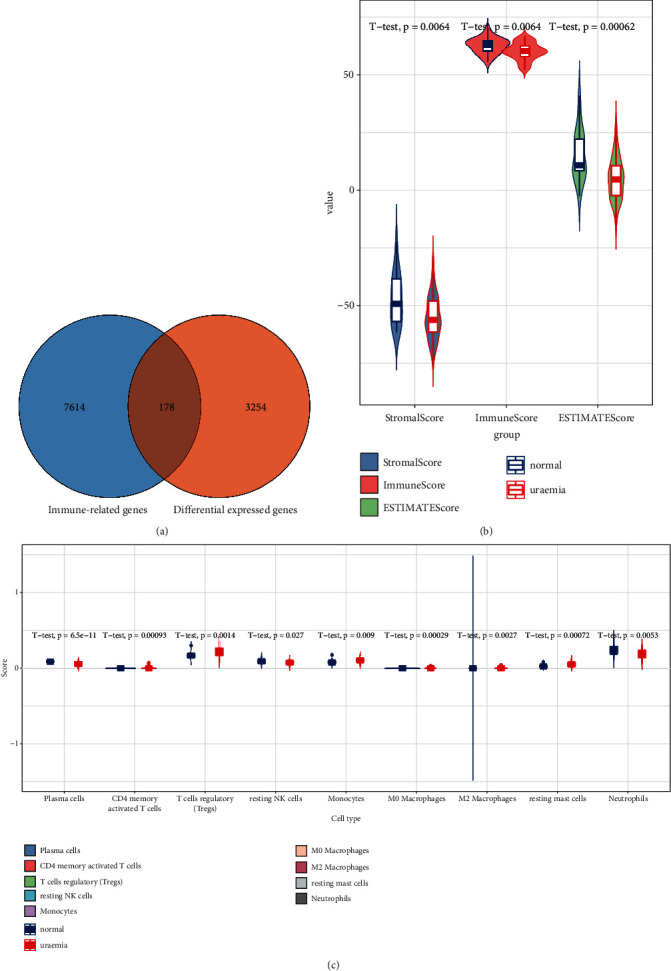
Immune-related genes. (a) Venn diagram for the overlapping comparison of immune-related genes and DEGs. (b) Immune infiltration. (c) Nine types of immune cells with significant differences in normal and uremic samples.

A total of 178 DEGs were identified as immune genes (Figure 4(a)). *T* test was used to analyze the differences of 22 types of immune cells in normal and uremic samples. The results reflected that there were significant differences in nine types of immune cells (*P* < 0.05), among which plasma cells, CD4 memory activated T cells, and T cells regulatory (Tregs; Figure 4(b)) ESTIMATE were used to analyze the level of immune infiltration. As could be seen from the graph, estimate that the normal individuals had relatively high level of immune infiltration and higher immune score (Figure 4(c)).

### 3.4. The Regulatory Network of TF

**Figure 5:**
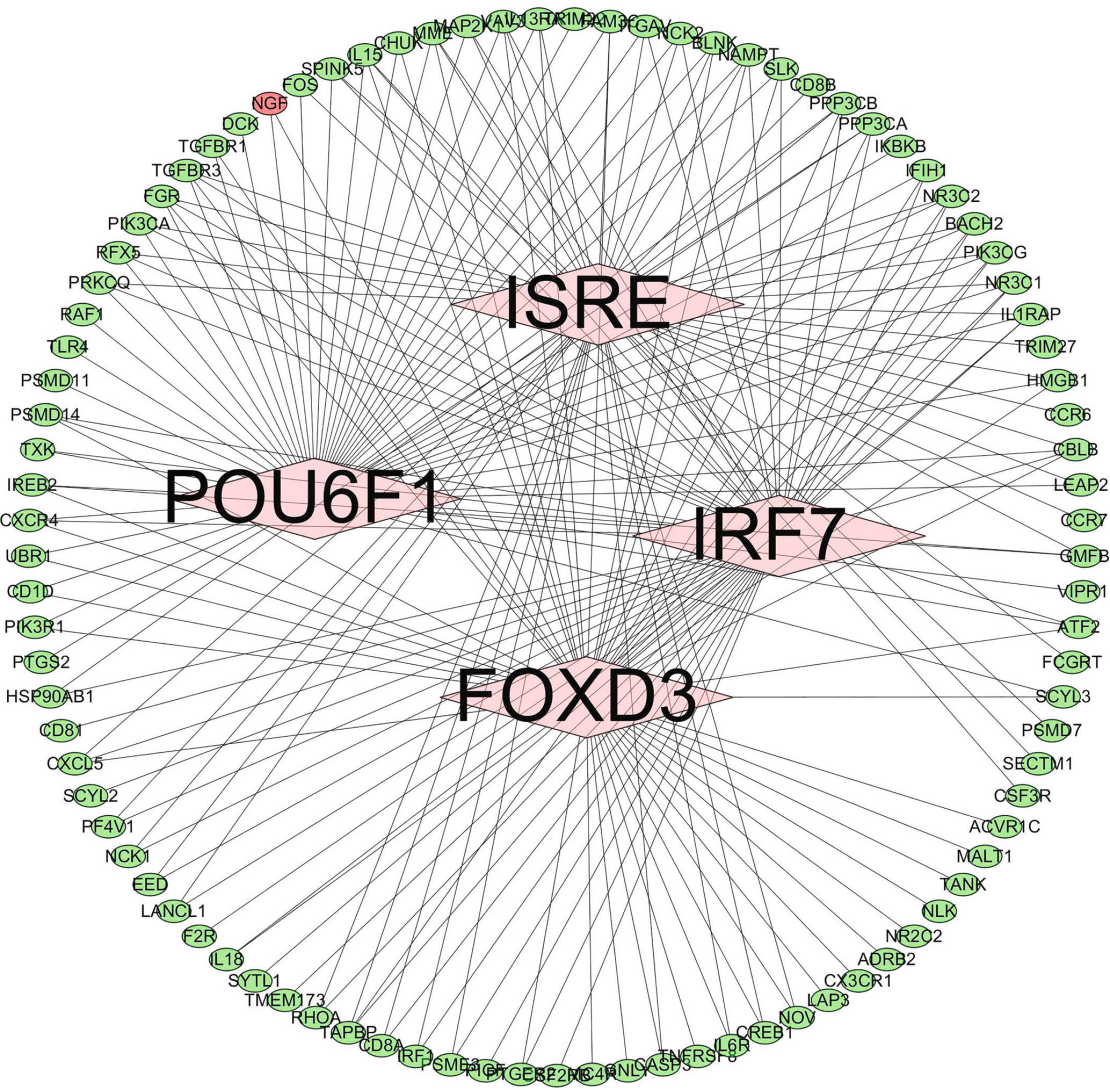
The regulatory network of transcription factor. The lines between two nodes mean that they have a subordinate relationship or can interact with each other.

The four TFs (POU domain class 6 transcription factor 1 (POU6F1), interferon regulator factor 7 [IRF7], forkhead box D3 (FOXD3), and interferon-stimulated response element [ISRE]) that could be relevant to immune-related genes were identified by David analysis. POU6F1 is a member of the POU TF family that binds favorably to a variant of the octamer motif (5′-ATGATAAT-3′) and contributes to activity-dependent circuit remodeling [18]. IRF7 is the main regulator of type I interferon (IFN) expression, which can form dimers with IRF3, and type I IFN expression was impaired in Irf7−/−plasmacytoid dendritic cell precursors (pDCs) or mouse embryonic fibroblast (MEFs) upon virus infection [19]. FOXD3 regulates the interleukin 10 (IL-10) promoter to facilitate the regulation of regulatory B (Breg) cell production [20]. ISRE is related to the specific transcriptional activation of IFNa and IFN-stimulated gene factor [21] (Figure 5). IRF7 regulated the most immune-related genes (87), FOXD3 the least (69), and POU6F1 and ISRE, 83 and 84, respectively. Fourteen immune-related genes were controlled by the four TFs, and these were fibroblast growth factor, transforming growth factor beta receptor III, IL-15, phosphatase 3 catalytic subunit beta, nuclear receptor subfamily 3 group C member 2, membrane metalloendopeptidase (VAV3), alpha-methyl-*para*-tyrosine, protein phosphatase catalytic subunit alpha, BTB and CNC homolog 2, phosphatidylinositol-4,5-bisphosphate 3-kinase catalytic subunit gamma, nuclear receptor subfamily 3 group C member 1, IL 1 receptor accessory protein, and Cbl proto-oncogene B.

### 3.5. Protein–Protein Interaction Network

**Figure 6:**
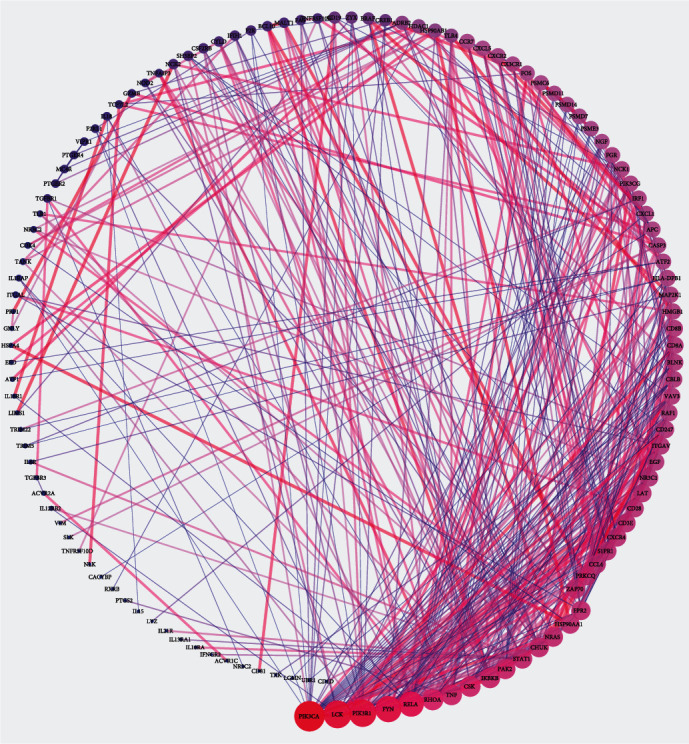
The exploration of PPI with 178 target genes using String. The area of the circle is proportional to the size of the degree.

The protein–protein interaction (PPI) network of 178 immune-related genes was analyzed by String. The confidence value of the interacting proteins was set to 0.9. The degree of each protein was presented by the size of the circle. The larger the circle, the greater the degree. The top 15 proteins with the highest degree in PPI were phosphatidylinositol-4,5-bisphosphate 3-kinase catalytic subunit alpha, lymphocyte-specific protein tyrosine kinase, phosphoinositide-3-kinase regulatory subunit 1, tyrosine protein kinase (FYN), v-rel reticuloendotheliosis viral oncogene homolog A, Ras homolog family member A, tumor necrosis factor, C-terminal Src kinase, inhibitor of kappa light polypeptide gene enhancer in B-cells kinase beta, p21 protein activated kinase 2, signal transducer and activator of transcription 1, conserved helix-loop-helix ubiquitous kinase, neuroblastoma RAS viral oncogene homolog, heat shock protein 90 alpha family class A member 1, and formyl peptide receptor type 2 (Figure 6). Meanwhile, the degree of proteins in the PPI network was also shown in the supplementary material.

### 3.6. The Role of Gender in Uremia

**Figure 7:**
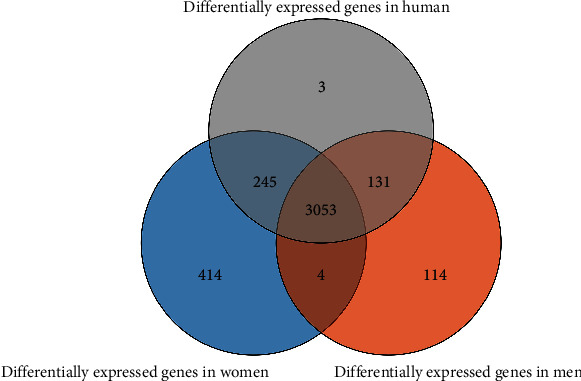
Venn diagram of DEGs. The DEGs were identified from the three microarray datasets showed overlaps of 3053 in both male and female.

The DEGs of 3302 were screened by comparing healthy males with sick males. In the same way, 3716 were screened out by comparing healthy female with female patients. It was noted that 3057 genes were to be differentially expressed by men and women, and 245 genes were restricted to men and 659 genes to women (Figure 7).

## 4. Discussion

Uremia is a progressive and irreversible disease that remains incurable despite dialysis and kidney transplants. In this study, we analyzed 3432 DEGs, including 3368 down-regulated genes and 64 up-regulated genes. Subsequently, we utilized bioinformatic methods to deeply explore the DEGs, including GO and KEGG pathway enrichment analyses, PPI network construction, and target genes. The GO and KEGG pathway analyses were conducted to explore interactions among the DEGs, including top 15 GO enrichment and top 10 pathways enrichment. The cause of uremia was proposed to be complicated [7], and Ying and Zhou [22] had verified end-stage renal failure had profound changes in human gene expressions.

In patients with ESRD, the two major causes of death in patients with ESRD, cardiovascular disease and infection, are both connected with the impaired immune response [2, 23, 24]. As activated T cells, monocytes and macrophages play critical roles in the formation of atherosclerotic plaques [25]. In the present study, a total of 178 DEGs were identified as immune genes, and nine types of immune cells had significant differences among twenty-two types in normal and uremic samples. The intrinsic function of T and B cells is normal when they are fitted with normal signaling from antigen-presenting cells (APCs) [26]. Patients with chronic renal failure show a defective function of APCs-derived costimulation leading to impaired activation of effector lymphocytes [27]. In other words, the abnormal APCs could not effectively activate T and B cells, so that the plasma cells decreased in uremia patients. Monocytes are precursors to dendritic cells, a group of the professional and highly efficient APCs in the immune system [28]. In patients with chronic renal failure, as well as those treated with maintenance hemodialysis, monocytes are largely dysregulated [29]. The contribution of monocytes to the dysregulated immune response in uremia can be based on altered features of individual cells or a change in the relative numbers of the three different populations [30]. A review came to the conclusion that the resting monocytes in the circulation of patients with CKD had a higher level of activation than in healthy individuals [29], which was consistent with the present result that the number of active monocytes in uremia was significantly higher in normal. It is possible to state that the level of immune infiltration in patients is relatively low, and the relationship between immunity and uremia is elaborated [31]. In our research, the normal individuals had relatively high level of immune infiltration and higher immune score; these results fall in line with historical findings. Renal failure led to decreasing erythropoietin (secreted by peritubular interstitial cells of the kidney) and vitamin D production, which adversely affected the immune system [31]. Immune-related genes were regulated by four TFs (POU6F1, IRF7, FOXD3, and ISRE) and top 15 proteins expressed with the highest degree identified in the PPI network, which could provide important information changes in cellular biology and function [32]. Shinohara et al. studied that renal failure was significantly associated with an increase in aortic pulse wave velocity regardless of gender, which was consistent with our research [33]. However, men began dialysis more likely before death, and the causal mechanisms were uncertain [34], perhaps it was considered from heredity, income, and role. Studies have disclosed that in both animals and humans shows a connection between male gender and a more rapid progression of renal diseases, independent of blood pressure and serum cholesterol level [35].

## 5. Conclusions and Outlook

In conclusion, we identified 3432 DEGs in patients with uremia compared with healthy individuals. The 178 immune-related genes were selected to establish the regulatory network of TFs and PPI network. Expression levels of DEGs were preliminarily detected according to the GSE37171 dataset. Our study indicated that DEGs could contribute to the progression of uremia by regulating biological processes, molecular function, and signaling pathways independent of gender. In the future, we expect several promising molecular markers will be found for kidney disease. However, only a small fraction was evaluated in populations of different samples. To validate its clinical utility, studies in a large representative population of specific kidney diseases are required.

## Data Availability

Data used in this paper can be downloaded from the original dataset GSE37171, http://www.ncbi.nlm.nih.gov/geohttps://cibersort.stanford.edu/index.php.

## Supplementary Materials

Supplementary Materials This supplementary information includes detailed information of 3432 differentially expressed genes, degree data, and immune-related genes regulated by four transcription factors.

## References

[1] Robinson B. M., Akizawa T., Jager K. J., Kerr P. G., Saran R., Pisoni R. L. (2016). Factors affecting outcomes in patients reaching end-stage kidney disease worldwide: differences in access to renal replacement therapy, modality use, and haemodialysis practices. *The Lancet*.

[2] Girndt M., Sester U., Sester M., Kaul H., Köhler H. (1999). Impaired cellular immune function in patients with end-stage renal failure. *Nephrology Dialysis Transplantation*.

[3] Meyer T. W., Hostetter T. H. (2014). Approaches to uremia. *Journal of the American Society of Nephrology*.

[4] Chiang C., Tanaka T., Nangaku M. (2012). Dysregulated oxygen metabolism of the kidney by uremic toxins: review. *Journal of Renal Nutrition*.

[5] Vanholder R., Pletinck A., Schepers E., Glorieux G. (2018). Biochemical and clinical impact of organic uremic retention solutes: a comprehensive update. *Toxins*.

[6] Ciceri P., Artioli L., Magagnoli L. (2022). The role of uremic retention solutes in the MIA syndrome in hemodialysis subjects. *Blood Purification*.

[7] Gusev E., Solomatina L., Zhuravleva Y., Sarapultsev A. (2021). The pathogenesis of end-stage renal disease from the standpoint of the theory of general pathological processes of inflammation. *International Journal of Molecular Sciences*.

[8] Niaudet P. (2010). Living donor kidney transplantation in patients with hereditary nephropathies. *Nature Reviews. Nephrology*.

[9] Ottlewski I., Münch J., Wagner T. (2019). Value of renal gene panel diagnostics in adults waiting for kidney transplantation due to undetermined end-stage renal disease. *Kidney International*.

[10] Teng P., Xu X., Ni C. (2020). Identification of key genes in calcific aortic valve disease by integrated bioinformatics analysis. *Medicine*.

[11] Hosszu A., Fekete A., Szabo A. J. (2020). Sex differences in renal ischemia/reperfusion injury. *American Journal of Physiology-Renal Physiology*.

[12] Muller V., Szabó A., Viklicky O. (1999). Sex hormones and gender-related differences: their influence on chronic renal allograft rejection. *Kidney International*.

[13] Carrero J. J., Hecking M., Chesnaye N. C., Jager K. J. (2018). Sex and gender disparities in the epidemiology and outcomes of chronic kidney disease. *Nature Reviews Nephrology*.

[14] Kanehisa M. (2002). The KEGG database. *Novartis Foundation Symposium*.

[15] Liston A., Dooley J., Yshii L. (2022). Brain-resident regulatory T cells and their role in health and disease. *Immunology Letters*.

[16] Ghassabi A., Motavalli R., Iranzad R. (2022). Potential contribution of the immune system to the emergence of renal diseases. *Immunology Letters*.

[17] Yi T. W., Levin A. (2022). Sex, gender, and cardiovascular disease in chronic kidney disease. *Seminars in Nephrology*.

[18] McClard C. K., Kochukov M. Y., Herman I. (2018). POU6f1 mediates neuropeptide-dependent plasticity in the adult brain. *The Journal of Neuroscience*.

[19] Shen Y. J., Lam A. R., Ho S. W. S., Koo C. X., Le Bert N., Gasser S. (2014). Cancer pathogenesis and DNA sensing. *Biological DNA Sensor*.

[20] Zhang Y., Wang Z., Xiao H. (2017). Foxd3 suppresses interleukin-10 expression in B cells. *Immunology*.

[21] Sheikh S. Z., Kobayashi T., Matsuoka K., Onyiah J. C., Plevy S. E. (2011). Characterization of an interferon-stimulated response element (ISRE) in the Il23a promoter. *Journal of Biological Chemistry*.

[22] Ying X. X., Zhou C. X. (2016). Comparing classification performance of several types of significant genes to identify key genes in uremia. *European Review for Medical and Pharmacological Sciences*.

[23] Betjes M. G. H. (2013). Immune cell dysfunction and inflammation in end-stage renal disease. *Nature Reviews. Nephrology*.

[24] Chebla R. B., Tamima H., Dagher G. A. (2021). Sepsis in end-stage renal disease patients: are they at an increased riskof mortality?. *Annals of Medicine*.

[25] Hansson G. K., Libby P. (2006). The immune response in atherosclerosis: a double-edged sword. *Nature Reviews Immunology*.

[26] Girndt M., Sester M., Sester U., Kaul H., Köhler H. (2001). Molecular aspects of T- and B-cell function in uremia. *Cellular Immune Defects in Uremia*.

[27] Girndt M., Sester M., Sester U., Kaul H., Kohler H. (2001). Molecular aspects of T- and B-cell function in uremia. *Kidney International Supplements*.

[28] Goxe B., Latour N., Bartholeyns J., Romet-Lemonne J. L., Chokri M. (1998). Monocyte-derived dendritic cells: development of a cellular processor for clinical applications. *Research in Immunology*.

[29] Girndt M., Trojanowicz B., Ulrich C. (2020). *Toxins*.

[30] Cohen G. (2020). Immune dysfunction in uremia 2020. *Toxins*.

[31] Haverty J. C. R. A. (1990). Vitamin D and immune function in uremia. *Basic Science and Dialysis*.

[32] Scherer A., Günther O. P., Balshaw R. F. (2013). Alteration of human blood cell transcriptome in uremia. *BMC Medical Genomics*.

[33] Shinohara K., Shoji T., Tsujimoto Y. (2004). Arterial stiffness in predialysis patients with uremia. *Kidney International*.

[34] Hecking M., Tu C., Zee J. (2022). Sex-specific differences in mortality and incident dialysis in the chronic kidney disease outcomes and practice patterns study. *Kidney International Reports*.

[35] Silbiger S. R., Neugarten J. (1995). The impact of gender on the progression of chronic renal disease. *American Journal of Kidney Diseases*.

