# Supplementary material for: Identification of Differentially Expressed Genes Particularly Associated with Immunity in Uremia Patients by Bioinformatic Analysis
Source: Anal Cell Pathol (Amst). 2022 Dec 29;2022:5437560. doi: 10.1155/2022/5437560 (PMC9815924; doi:10.1155/2022/5437560)
Supplement: Supplementary Materials — This supplementary information includes detailed information of 3432 differentially expressed genes, degree data, and immune-related genes regulated by four transcription factors. [file 5437560.f1.pdf]

Differentially expressed genes

Gene ID	Log_2(Fold Change)	Average Expression	P.Value	adjust P.Value	Benjamini
PIGL	-1.491955546	3.892655688	1.83E-66	3.95E-62	141.222208
DCLRE1A	-1.966083899	4.545018107	9.12E-66	9.88E-62	139.640398
ZFP30	-2.395371558	3.321088347	7.18E-64	5.19E-60	135.340517
TRADD	-1.296522309	7.193819784	1.13E-63	6.13E-60	134.891461
CALU	-1.141605685	4.961123052	2.25E-63	9.76E-60	134.212644
TAF1	-1.432216339	4.904520278	2.91E-63	1.05E-59	133.962308
YRDC	-1.673056925	5.384928036	1.01E-62	3.14E-59	132.728485
ZNF213-AS1	-1.787303352	3.282177997	1.33E-62	3.59E-59	132.463579
SLC39A13	-1.665456812	5.310808605	6.95E-62	1.67E-58	130.828594
UQCC1	-1.311684792	4.774210987	1.23E-61	2.67E-58	130.262207
RP11-61L19.3	-2.023988072	3.449154531	4.01E-58	7.24E-55	122.257809
PARG	-2.382012188	4.438969804	6.30E-58	1.05E-54	121.810208
ENTPD7	-1.320760213	4.414523487	8.18E-58	1.27E-54	121.551188
PIGW	-2.278412246	3.192312168	2.66E-57	3.84E-54	120.381875
ZNF419	-1.548640323	5.546434358	9.98E-57	1.35E-53	119.069972
ZNF626	-1.300204755	3.727575735	6.90E-56	8.79E-53	117.150883
OTUD3	-2.167153797	3.868900616	1.08E-55	1.30E-52	116.706165
FAM219B	-1.033461442	5.392349589	4.00E-55	4.33E-52	115.404434
NRF1	-1.10871798	4.913572685	5.97E-55	6.16E-52	115.006666
RP11-140I16.3	-1.926054848	3.542831623	9.29E-55	9.14E-52	114.568057
BHMT	-1.78511677	4.122986819	1.16E-54	1.10E-51	114.343583
ACTR3B	-1.412197505	4.766670919	2.43E-54	2.19E-51	113.612444
GTF3C4	-1.210480012	4.532651495	4.81E-54	4.17E-51	112.932649
EXTL3-AS1	-1.449904193	2.792068865	5.76E-54	4.62E-51	112.753975
KIF3A	-2.041730081	3.761908054	5.87E-54	4.62E-51	112.734781
BC047484	-2.24910512	3.581283495	5.97E-54	4.62E-51	112.718726
ZNF789	-1.126723982	3.871701841	6.77E-54	5.06E-51	112.593257
AIG1	-1.305247316	3.499670151	7.89E-54	5.70E-51	112.440588
RP11-29H23.4	-2.158769987	3.578391595	1.61E-53	1.12E-50	111.730202
KIAA0391	-1.516866381	3.579127035	1.66E-53	1.12E-50	111.701924
PIGN	-1.467052472	4.000823018	2.83E-53	1.86E-50	111.168912
RP11-5C23.2	-1.899904852	4.111746175	4.48E-53	2.85E-50	110.713617
NBPF20	-1.648725333	3.755663589	5.71E-53	3.53E-50	110.471981
LINC00667	-1.294406189	4.305317251	7.31E-53	4.40E-50	110.226078
CDK2	-1.193928632	3.909854354	8.03E-53	4.70E-50	110.133407
SNORA72	-1.373636799	2.666057178	9.00E-53	5.13E-50	110.019614
BMF	-1.671995565	4.938634808	9.91E-53	5.50E-50	109.923604
ABCB1	-1.564389189	3.619348808	1.24E-52	6.70E-50	109.702273
PCGF6	-1.764627375	3.706667565	1.84E-52	9.71E-50	109.309008
ANKLE2	-1.364628649	4.450573436	1.92E-52	9.88E-50	109.267568
SMURF1	-1.033516379	4.282476009	3.23E-52	1.63E-49	108.747992
LIX1L	-1.976889257	5.60847267	4.85E-52	2.33E-49	108.343507
SLC25A15	-1.579863867	4.405526674	5.44E-52	2.56E-49	108.229084
UTP15	-1.09229198	3.06589816	5.69E-52	2.62E-49	108.184188
ZNF75D	-1.469568046	3.67774556	7.85E-52	3.54E-49	107.863444
UEVLD	-1.500748097	4.92085537	9.40E-52	4.15E-49	107.684671
LOC100129198	-1.689641406	3.535256428	9.89E-52	4.24E-49	107.63355
SCML2	-1.849324505	4.413730109	1.80E-51	7.37E-49	107.034572
CWF19L1	-1.232606433	5.64526445	2.93E-51	1.18E-48	106.551466
E2F5	-2.170400453	3.341950065	3.14E-51	1.24E-48	106.481562
SNHG22	-1.602603192	3.804606354	3.28E-51	1.27E-48	106.441035
GCFC2	-1.472474117	3.162769039	3.58E-51	1.36E-48	106.352831
SH3BP2	-1.168588751	5.456261184	3.74E-51	1.40E-48	106.307688
FAM188A	-1.517482019	3.076504265	4.67E-51	1.71E-48	106.087869
HMGCS1	-1.174174729	3.396134393	6.20E-51	2.24E-48	105.805125
NUP205	-1.768381981	4.561485245	6.42E-51	2.27E-48	105.770708

CXorf24	-2.290713472	3.887161302	6.50E-51	2.27E-48	105.758569
NDUFAF7	-1.36337104	3.640821853	7.58E-51	2.61E-48	105.604703
LOC100996251	-1.304857301	3.029624862	7.92E-51	2.68E-48	105.561266
ERCC4	-1.389360232	4.121700901	9.78E-51	3.26E-48	105.351305
FRMD8	-1.347549674	5.613445107	1.14E-50	3.73E-48	105.200687
ZNF43	-2.114387205	3.960416503	1.18E-50	3.82E-48	105.163663
LSR	-2.291188301	4.772256075	1.26E-50	4.01E-48	105.09944
MUS81	-1.421901776	5.522765301	1.35E-50	4.25E-48	105.028056
HHAT	-1.615153587	3.481836933	3.56E-50	1.10E-47	104.064855
MEAF6	-1.406968929	5.098547229	3.83E-50	1.17E-47	103.990579
AL109706	-1.385506094	3.344345208	4.19E-50	1.26E-47	103.901021
PTPRM	-1.467878173	3.373004858	4.78E-50	1.42E-47	103.769674
POLR2D	-1.712713158	3.984945205	5.65E-50	1.65E-47	103.603609
PARP15	-1.481447848	3.42764617	6.74E-50	1.95E-47	103.427312
CTC-338M12.4	-1.187540348	4.017747948	7.15E-50	2.04E-47	103.36909
SLC30A6	-1.461758228	5.005905041	8.86E-50	2.49E-47	103.15572
ZCCHC18	-1.898690216	4.411669431	9.54E-50	2.62E-47	103.081477
FUT8	-1.158125224	4.172631088	1.01E-49	2.74E-47	103.023384
LOC101928708	-1.602159402	3.718919033	1.17E-49	3.09E-47	102.87866
DNAJC14	-1.542273163	5.15283157	1.49E-49	3.89E-47	102.636961
ZNF85	-1.067872088	3.493776097	1.58E-49	4.07E-47	102.579326
RP11-45M22.3	-1.331639483	5.199004182	1.65E-49	4.21E-47	102.533424
ZNF717	-1.166478746	4.137398811	1.67E-49	4.21E-47	102.523325
FAM149B1	-1.007765413	3.517306324	1.93E-49	4.81E-47	102.378708
WEE1	-1.5395708	4.281599297	1.96E-49	4.82E-47	102.363692
RP5-1085F17.3	-1.246645751	5.279127743	2.22E-49	5.39E-47	102.240804
ADAT2	-1.13465494	3.95714032	2.48E-49	5.96E-47	102.130955
EXOSC6	-1.364352639	4.660633623	3.29E-49	7.82E-47	101.847997
RP11-454F8.2	-1.446596892	4.010690134	3.86E-49	9.08E-47	101.68864
SEC22C	-1.084914282	4.853189607	4.77E-49	1.11E-46	101.476408
ZNF329	-1.998724229	3.859262108	5.38E-49	1.24E-46	101.357602
LIN52	-1.34650126	4.668805168	7.15E-49	1.60E-46	101.072969
ZBED4	-1.695341213	3.802254524	1.06E-48	2.28E-46	100.685201
RP4-714D9.5	-1.663105518	3.52004857	1.56E-48	3.34E-46	100.297485
MTA3	-1.16992634	4.564782374	1.62E-48	3.45E-46	100.254649
ZNF30	-1.590245907	3.397216346	1.70E-48	3.54E-46	100.209451
HIGD2A	-1.4470604	6.240600424	1.93E-48	3.97E-46	100.084438
ZBED6CL	-1.594292737	5.669429282	2.57E-48	5.25E-46	99.7977292
TDRD3	-1.320750343	3.595441477	3.19E-48	6.45E-46	99.58253
SEC24B-AS1	-1.870214573	4.105530855	3.41E-48	6.84E-46	99.514667
APEX2	-1.241730451	5.609551429	3.57E-48	7.09E-46	99.4697674
MRPL44	-1.962016834	4.106334863	3.74E-48	7.36E-46	99.423686
DNAJC27	-1.13109129	3.859249073	4.08E-48	7.90E-46	99.335228
RAB9B	-1.389349324	3.756591557	4.70E-48	9.00E-46	99.1957533
UCKL1	-1.101324255	5.651294747	5.31E-48	1.01E-45	99.0724918
GRAMD4	-1.242082481	5.2491389	6.01E-48	1.13E-45	98.9495069
LINC01278	-1.471016044	4.479961532	6.27E-48	1.17E-45	98.90771
TMEM169	-1.36036616	3.872289076	9.34E-48	1.73E-45	98.5100929
RXRB	-1.231277604	5.65923959	9.93E-48	1.81E-45	98.4482533
DNAAF2	-2.179677485	3.023420874	9.93E-48	1.81E-45	98.4482229
ZSCAN25	-1.194431976	5.271632678	1.19E-47	2.15E-45	98.2653322
NAF1	-1.832060537	2.927884206	1.21E-47	2.16E-45	98.2530129
LOC101929787	-1.99890882	3.466325543	1.35E-47	2.40E-45	98.1392231
MBIP	-1.559926256	3.070088837	1.51E-47	2.66E-45	98.029402
AC114730.11	1.172833623	3.840967236	2.05E-47	3.55E-45	97.7246869
USP21	-1.489220511	6.106421485	2.35E-47	4.04E-45	97.5880812
SPATA2	-1.471267413	4.966947876	2.37E-47	4.04E-45	97.5804526
LINC00928	-1.374452221	3.726719063	2.48E-47	4.19E-45	97.5371265

PPP1R14D	1.640621679	5.083354495	2.82E-47	4.73E-45	97.4088068
FKRP	-1.207868027	5.060583406	3.03E-47	5.01E-45	97.3349633
DUSP10	-1.295710616	4.853437019	4.11E-47	6.70E-45	97.0319604
HELQ	-1.329168634	3.973829511	4.11E-47	6.70E-45	97.0300972
SLC9B2	-1.433052192	4.144693821	4.30E-47	6.94E-45	96.9871399
ZSCAN22	-1.539202905	4.026478734	4.75E-47	7.62E-45	96.887227
GTF2I	-1.529871542	3.466802383	5.57E-47	8.87E-45	96.7274353
ING1	-1.553582	5.113461611	6.15E-47	9.72E-45	96.6294482
GLTSCR1	-1.297653302	5.768496582	6.82E-47	1.06E-44	96.5263476
BRD1	-2.479110065	4.75329296	9.60E-47	1.48E-44	96.1844792
ZNF440	-1.164795303	3.23109322	1.19E-46	1.83E-44	95.9659817
TMCO4	-1.221011225	5.476924421	1.25E-46	1.91E-44	95.9182309
SCAI	-1.091741905	3.350404902	1.30E-46	1.96E-44	95.8842629
DDX52	-1.264131461	4.376038983	1.57E-46	2.36E-44	95.6952208
TRIM32	-1.180148392	4.096906894	1.66E-46	2.47E-44	95.6399073
ATE1	-1.113540778	4.11312206	1.67E-46	2.48E-44	95.6298505
LOC101928954	-1.443804859	4.358590329	1.69E-46	2.49E-44	95.619922
TIMM10B	-1.60555357	5.181057573	1.88E-46	2.74E-44	95.5121518
CDC23	-1.702734094	3.670652047	2.01E-46	2.91E-44	95.4454221
RP11-111M22.4	-1.421132373	3.870152938	2.13E-46	3.05E-44	95.3895276
CMTM4	-1.042040905	4.447753133	2.32E-46	3.29E-44	95.3017945
DNA2	-1.858533005	3.303306305	2.64E-46	3.71E-44	95.1755994
RALGAPA1	-1.108409625	3.908983617	2.73E-46	3.81E-44	95.1413021
C8orf37	-1.213333359	2.922502901	2.78E-46	3.86E-44	95.123108
ZBTB25	-1.347738811	4.760207805	2.83E-46	3.90E-44	95.1057897
GALNT12	-1.650395884	3.885572686	2.87E-46	3.93E-44	95.0920709
ABHD17C	-1.518051978	3.739745403	3.89E-46	5.27E-44	94.7873238
FAM73A	-1.875611939	3.103493772	3.99E-46	5.37E-44	94.7609308
WDR91	-1.043284173	4.651145267	4.65E-46	6.17E-44	94.6100138
NUBPL	-1.795778314	3.311975442	5.14E-46	6.78E-44	94.5100697
CNOT4	-1.072485791	4.159295454	5.40E-46	7.09E-44	94.4599438
RNH1	-1.012314881	6.135293258	5.65E-46	7.37E-44	94.4146163
LPAR5	-1.639086024	4.761289699	6.64E-46	8.61E-44	94.2535765
N4BP2L2-IT2	-1.812378292	4.221038727	7.00E-46	9.03E-44	94.2001642
FAM168A	-1.621345891	4.988997833	7.57E-46	9.65E-44	94.1222756
LSM11	-1.024008898	3.79427263	8.28E-46	1.05E-43	94.0328264
IL18	-1.771099308	3.270724366	8.88E-46	1.12E-43	93.9628137
STT3A	-1.698031774	5.081178901	8.92E-46	1.12E-43	93.9586032
NOM1	-1.494546134	4.112157172	1.66E-45	2.01E-43	93.3411899
CHD6	-1.144007501	3.765949225	2.69E-45	3.26E-43	92.8547082
WDR20	-1.762504964	3.812879175	2.72E-45	3.27E-43	92.8464776
ZNF792	-1.279772014	2.742676553	3.12E-45	3.74E-43	92.7073376
ERCC6L2	-1.191732615	3.721951668	3.35E-45	3.96E-43	92.6382914
ZNF548	-1.082052001	4.357412847	3.41E-45	4.01E-43	92.620298
LOC100506603	-1.369102698	4.375347517	3.55E-45	4.16E-43	92.5784075
SPRTN	-1.600086981	2.821339039	4.33E-45	5.04E-43	92.3798525
RP11-379F4.6	-2.281170416	4.176369341	4.35E-45	5.04E-43	92.3760671
YWHAE	-1.763461462	6.165295131	4.86E-45	5.60E-43	92.2646481
RP11-53O19.3	-1.707293124	3.441395356	5.40E-45	6.19E-43	92.1594199
KDELC2	-1.743905239	3.977500206	5.58E-45	6.36E-43	92.1265893
FW340027	-2.366760472	4.253216847	6.00E-45	6.80E-43	92.0548554
CYP2R1	-1.251612272	3.750881346	6.90E-45	7.78E-43	91.9152911
SMKR1	-1.245789483	2.824664587	7.86E-45	8.82E-43	91.7851757
ZNF382	-1.029513448	2.765204932	8.37E-45	9.32E-43	91.7223735
LINC00865	-1.165141561	3.736261378	8.39E-45	9.32E-43	91.7194021
DDX19B	-1.532079928	4.735331523	9.66E-45	1.07E-42	91.5788316
MAPKAPK2	-1.138391507	6.208804841	9.98E-45	1.09E-42	91.5469292
USP54	-1.130552022	4.242449454	1.04E-44	1.13E-42	91.5027868

MRPS25	-1.050250462	5.243548315	1.14E-44	1.23E-42	91.4099309
GOPC	-1.296998774	4.223324317	1.32E-44	1.41E-42	91.26976
ZNF507	-1.102512748	3.890802642	1.39E-44	1.47E-42	91.2170318
MCM3AP-AS1	-1.223911399	3.17306646	1.44E-44	1.52E-42	91.1783751
RP11-410C4.5	-1.419369887	4.664321823	1.49E-44	1.57E-42	91.1444354
IPPK	-1.229073406	5.170494756	1.52E-44	1.59E-42	91.1278355
LEAP2	-1.696173615	4.9767518	1.62E-44	1.69E-42	91.0629192
CCDC66	-1.594972536	3.396659563	1.74E-44	1.80E-42	90.9896226
DKFZp564H213	-1.36587367	3.658834792	1.75E-44	1.80E-42	90.9838592
DHCR7	-1.116466662	4.568657872	1.96E-44	2.00E-42	90.8718112
SLC35F2	-1.266231125	4.130804153	2.27E-44	2.29E-42	90.7250834
TBC1D24	-1.452547108	3.890774997	3.53E-44	3.52E-42	90.2838758
LINC00342	-1.671681253	4.654371312	4.02E-44	3.99E-42	90.1547935
FMO5	-1.13567492	3.718407423	4.38E-44	4.33E-42	90.0685013
RP11-121C2.2	-1.742799793	3.956897538	4.56E-44	4.49E-42	90.0288584
SIGMAR1	-1.28415658	5.491016148	4.78E-44	4.69E-42	89.9805121
TMEM14A	-2.422154881	4.526964405	6.39E-44	6.18E-42	89.6906852
POLG2	-1.26177455	5.52813604	9.25E-44	8.90E-42	89.322034
SPDL1	-1.305158025	3.380445253	1.05E-43	1.00E-41	89.1967499
PITPNM2	-1.039059687	4.87159549	1.09E-43	1.04E-41	89.1549082
ERLIN2	-1.551736491	3.826311937	1.18E-43	1.12E-41	89.0815139
POLR1B	-1.531953903	5.004697308	1.38E-43	1.29E-41	88.9220499
ITGA10	-1.092152306	4.959412845	1.66E-43	1.54E-41	88.7360399
ZSCAN9	-1.106327765	4.093349715	1.73E-43	1.60E-41	88.6974563
ZNF284	-1.009568409	5.226661748	1.79E-43	1.65E-41	88.6592386
TNFRSF10D	-1.015116221	3.87029131	1.96E-43	1.80E-41	88.5727403
CHIC1	-1.045788095	3.427464515	1.98E-43	1.81E-41	88.5604376
ARHGEF9	-1.416310713	4.002472293	1.99E-43	1.81E-41	88.5552521
VPS13A	-1.583674055	3.867003499	2.07E-43	1.88E-41	88.5147624
WDYHV1	-1.889905259	3.643406975	2.35E-43	2.11E-41	88.3886751
MSANTD3	-1.154952768	5.079516624	2.89E-43	2.54E-41	88.183587
RNF8	-1.027613194	4.516675845	2.94E-43	2.58E-41	88.1662959
POLR3F	-1.435064041	3.808560707	3.25E-43	2.84E-41	88.0642548
ACTR5	-1.284983084	5.346830801	3.43E-43	2.97E-41	88.0129997
SS18	-1.160183538	3.936206834	3.43E-43	2.97E-41	88.0112625
YIPF4	-1.613945965	3.728111569	3.46E-43	2.98E-41	88.0041488
USP30	-1.512477073	4.19710164	4.41E-43	3.77E-41	87.7612481
FBXL12	-1.032606355	5.581195389	4.63E-43	3.95E-41	87.7109001
IQCE	-1.227627718	4.60952685	4.72E-43	4.00E-41	87.6934073
UBTD2	-1.993247381	4.124970382	5.10E-43	4.29E-41	87.6159511
CDK13	-1.196638704	5.586428545	5.69E-43	4.77E-41	87.5064635
PEX1	-1.841017378	3.538952976	5.83E-43	4.86E-41	87.4814243
MED14	-1.094806381	3.876264281	6.29E-43	5.22E-41	87.4058944
BAP1	-1.389174796	6.064936001	6.56E-43	5.42E-41	87.3633731
ALDH18A1	-1.512630024	4.938138778	7.06E-43	5.81E-41	87.290383
APIP	-1.487912921	3.230929431	7.24E-43	5.93E-41	87.2655523
NASP	-1.271332936	5.909882349	7.34E-43	5.98E-41	87.2505131
NHLRC3	-1.618543243	3.506885776	7.35E-43	5.98E-41	87.2499153
TADA1	-1.436340534	3.567028661	7.50E-43	6.08E-41	87.229843
ACTR1B	-1.184009693	7.108416182	7.92E-43	6.40E-41	87.1755913
IPMK	-1.335145239	3.218511048	9.12E-43	7.34E-41	87.0342644
RAD51D	-1.359892308	4.459931456	9.15E-43	7.34E-41	87.0307859
GGCX	-1.061886537	4.561848463	9.58E-43	7.63E-41	86.9848096
CTPS2	-1.065611025	3.653080554	1.05E-42	8.30E-41	86.8929773
RAB30	-1.40357588	4.071559743	1.14E-42	9.02E-41	86.8067314
RSPH3	-1.238602599	5.388636593	1.17E-42	9.15E-41	86.7878663
ODF2	-1.373571413	4.4023336	1.19E-42	9.27E-41	86.7677889
CCP110	-1.493766899	4.369038266	1.32E-42	1.02E-40	86.6676494

FAM173B	-1.223742907	4.143949257	1.33E-42	1.03E-40	86.6592412
TIMM23B	-2.008782446	4.087490673	1.68E-42	1.29E-40	86.4215023
SEC24D	-1.487482673	3.699958919	1.93E-42	1.47E-40	86.286464
ABCC1	-1.509110297	5.627044079	1.99E-42	1.51E-40	86.2545995
CWC22	-1.870889326	4.010821082	2.32E-42	1.75E-40	86.1011229
GAB3	-1.181095297	5.610391261	2.34E-42	1.76E-40	86.0917017
CSRNP2	-1.326948827	4.921889002	2.41E-42	1.81E-40	86.0627969
METTL21B	-1.364360717	3.929145962	2.67E-42	1.99E-40	85.9604446
SDF4	-1.331455287	7.570098249	2.82E-42	2.10E-40	85.9058602
CHORDC1	-1.869212532	3.637271297	2.93E-42	2.16E-40	85.8678889
ZNF701	-1.261409571	4.213815072	3.02E-42	2.21E-40	85.8383172
PDSS1	-1.422969263	4.890154371	3.19E-42	2.33E-40	85.7836003
ZNF623	-1.792534809	3.43973131	3.24E-42	2.36E-40	85.7673664
SETBP1	-1.375390122	4.678449581	3.28E-42	2.38E-40	85.7538514
RP11-252E2.1	-1.185769222	4.351390926	3.50E-42	2.53E-40	85.6880608
UBASH3B	-1.142975034	4.454253008	3.51E-42	2.53E-40	85.6870723
THUMPD2	-1.442743133	3.818879406	4.00E-42	2.86E-40	85.5554144
NFS1	-1.128107159	4.446631177	4.11E-42	2.92E-40	85.5298635
ARMCX1	-1.196513367	2.649682328	4.15E-42	2.94E-40	85.5182975
LOC100505564	-1.488245494	3.58067903	4.17E-42	2.94E-40	85.5150658
FDXACB1	-1.292436803	4.268826247	4.28E-42	3.00E-40	85.4885084
CCDC64	-1.111001496	4.542709881	4.48E-42	3.13E-40	85.4414683
LOC101928963	-1.589804302	3.006297396	5.13E-42	3.57E-40	85.3066551
CYP27B1	-1.485716419	4.290348782	5.31E-42	3.68E-40	85.2731094
FAM84B	-1.297110852	4.839086177	5.52E-42	3.81E-40	85.2330364
C1orf216	-1.691947372	5.412186734	5.99E-42	4.10E-40	85.1522169
CARNS1	-1.450465238	5.322029617	6.46E-42	4.41E-40	85.0765035
TMEM206	-1.257223135	3.922746928	6.55E-42	4.46E-40	85.0630282
KCTD13	-1.301988308	5.363902442	7.30E-42	4.96E-40	84.9537231
MTM1	-1.635502823	3.056927632	9.08E-42	6.01E-40	84.7359653
NNT	-1.397304785	4.332426338	9.93E-42	6.56E-40	84.6466736
FAM208B	-1.511317147	4.418332912	9.99E-42	6.57E-40	84.6410162
LOC221272	-1.193231055	3.155686428	1.08E-41	7.09E-40	84.5621585
DBP	-1.130723008	5.53725404	1.22E-41	7.99E-40	84.4390758
PIAS3	-1.474225652	4.735615402	1.35E-41	8.77E-40	84.3409899
LOC101927416	-1.055834346	3.46190763	1.37E-41	8.89E-40	84.3242885
LOC100130428	-1.217243702	3.183161987	1.39E-41	9.01E-40	84.3077064
EIF1AD	-1.145622588	5.599161016	1.45E-41	9.34E-40	84.2689679
CCDC77	-1.332993297	3.525517859	1.58E-41	1.01E-39	84.1846384
KLC2	-1.672680651	5.184477713	1.60E-41	1.02E-39	84.1708561
LMAN1	-1.321684956	4.524663892	1.70E-41	1.08E-39	84.1102497
NCL	-1.246459411	6.108296113	1.78E-41	1.13E-39	84.0604767
C6orf203	-1.690353521	3.643898609	2.05E-41	1.28E-39	83.9238397
AKAP8	-1.158988658	5.829870023	2.07E-41	1.29E-39	83.9119481
CPSF2	-1.891600079	4.47984068	2.61E-41	1.61E-39	83.6818912
GDI1	-1.548870805	8.170110232	2.71E-41	1.67E-39	83.6431382
TMTC2	-1.130659825	3.70878514	2.74E-41	1.68E-39	83.6319435
BTBD17	1.423864987	5.046577906	2.79E-41	1.71E-39	83.6122038
RP11-157B13.7	-1.173225419	4.050997389	2.91E-41	1.77E-39	83.5728978
VPS45	-1.039744324	5.619822997	3.09E-41	1.88E-39	83.5105408
PRR3	-1.463360462	4.892611113	3.21E-41	1.94E-39	83.472749
ZNF230	-1.313093465	3.841970341	3.24E-41	1.96E-39	83.4626392
COQ10A	-1.423333877	5.891678328	3.45E-41	2.08E-39	83.4004006
KIAA1407	-1.416001952	3.980174322	3.60E-41	2.15E-39	83.3585717
ARMC2	-1.259847106	3.200390138	3.94E-41	2.35E-39	83.268395
SASS6	-1.296520037	2.932208815	3.95E-41	2.35E-39	83.2646218
CHKA	-1.010111898	4.833028462	4.06E-41	2.41E-39	83.2373319
CIAO1	-1.323612531	5.480389975	4.08E-41	2.42E-39	83.2320744

FAM178A	-1.212392676	4.625011535	4.27E-41	2.52E-39	83.1879316
MRPL42	-1.652487345	4.078591809	4.61E-41	2.70E-39	83.1108577
RELA	-1.350728478	6.776522164	4.64E-41	2.71E-39	83.103836
ZCCHC9	-2.120691344	5.043248703	4.66E-41	2.72E-39	83.0993996
CYP26A1	1.252620272	4.448143641	4.97E-41	2.89E-39	83.0355531
PHAX	-1.809245539	4.5202657	5.08E-41	2.93E-39	83.0136433
USP53	-1.330395901	3.020412158	5.17E-41	2.98E-39	82.9961002
LOC101928483	-1.311810557	4.22980574	5.44E-41	3.13E-39	82.9451553
HOMER1	-1.127859056	3.777475728	5.52E-41	3.16E-39	82.9309341
MTMR2	-1.107534231	4.337672467	5.61E-41	3.20E-39	82.9153775
PSRC1	-1.326699676	5.189358563	5.64E-41	3.21E-39	82.9092708
RPE	-1.245644062	3.60071558	5.76E-41	3.27E-39	82.8891506
UBAP2L	-1.035382143	6.06976449	6.15E-41	3.48E-39	82.823357
ELP4	-1.345225651	5.322212815	6.19E-41	3.49E-39	82.8166909
TTC8	-1.0642592	3.163202051	6.39E-41	3.59E-39	82.784395
ZNF512B	-1.215301764	3.662319606	6.86E-41	3.85E-39	82.714108
TRAF1	-1.096910495	4.732428003	7.08E-41	3.96E-39	82.6817819
RP11-5C23.1	-1.76296299	4.863308528	7.35E-41	4.08E-39	82.6440704
LCT	-1.141342287	3.960888731	7.40E-41	4.10E-39	82.6372562
LOC101930097	-1.290396004	5.274461586	7.83E-41	4.33E-39	82.5809402
DOCK9	-1.02274113	4.10671154	8.78E-41	4.82E-39	82.4671623
FAM63B	-1.787900982	4.099237496	9.68E-41	5.29E-39	82.3691507
AX747405	-1.224294891	3.63789129	1.02E-40	5.56E-39	82.3177453
COX17	-1.839282855	3.872109097	1.23E-40	6.67E-39	82.1307711
NDRG3	-1.463800331	5.82019739	1.49E-40	8.02E-39	81.9407915
CCDC134	-1.079740698	4.579534763	1.51E-40	8.14E-39	81.9230518
PEX14	-1.190821343	4.825936052	1.55E-40	8.33E-39	81.8973646
WDR5B	-1.204442531	3.710704282	1.57E-40	8.44E-39	81.8826225
RP11-326I11.5	-1.351710242	3.268315722	1.68E-40	8.97E-39	81.8190926
LOC101927620	-1.539304161	4.306675963	2.84E-40	1.49E-38	81.292152
FAM86C1	-1.325846375	3.615333297	2.93E-40	1.53E-38	81.2608759
FKSG29	-1.093894219	3.448032604	2.95E-40	1.54E-38	81.2552569
ATP10B	-1.204789092	4.243016706	3.17E-40	1.65E-38	81.1816264
ZNF827	-1.248986567	4.579456448	3.35E-40	1.74E-38	81.1267918
C1QTNF3	-1.042210783	4.141706198	4.49E-40	2.31E-38	80.8349698
STK36	-1.052099514	5.125466277	5.67E-40	2.91E-38	80.6008573
VAPB	-1.290737292	5.196934298	5.96E-40	3.05E-38	80.5513323
STRIP1	-1.604295469	6.203787786	6.09E-40	3.11E-38	80.5284587
NUP160	-1.666418175	3.678292255	6.51E-40	3.31E-38	80.462693
FDX1	-1.679463324	4.120103212	6.77E-40	3.43E-38	80.422674
ZNF486	-1.089691576	3.59907827	6.93E-40	3.50E-38	80.3996818
CTD-2528L19.6	-1.583176659	2.86626152	7.38E-40	3.70E-38	80.3362283
ZNF25	-1.132430666	3.64661965	7.42E-40	3.70E-38	80.3306351
NCAPD3	-1.407454126	5.354008883	7.43E-40	3.70E-38	80.3304397
CMSS1	-1.607142403	4.428720515	7.49E-40	3.72E-38	80.3216733
RP5-935K16.1	-1.879544142	5.101951385	7.58E-40	3.75E-38	80.3102278
ZNF831	-1.595320143	4.08997235	7.96E-40	3.93E-38	80.2608149
DSTYK	-1.100916214	4.920833903	8.22E-40	4.05E-38	80.2289696
FCRL1	-2.02309561	4.183971396	8.38E-40	4.12E-38	80.2091234
SPINT2	-1.514986127	6.786777817	8.47E-40	4.15E-38	80.1985907
DCAF17	-1.251447806	3.014908341	8.49E-40	4.15E-38	80.1966442
NELFB	-1.814820736	5.288437307	9.07E-40	4.42E-38	80.1303838
ZNF879	-1.401884485	3.891643448	9.09E-40	4.42E-38	80.1279228
ZNF490	-1.337951291	4.518756882	9.38E-40	4.55E-38	80.0969767
ZNF510	-1.3511926	4.481488753	9.57E-40	4.64E-38	80.0768314
RRN3P1	-1.290036656	5.226010986	9.69E-40	4.69E-38	80.063851
SRFBP1	-2.046969267	3.545248904	9.98E-40	4.81E-38	80.0351429
EGF	-1.584091448	3.13264796	1.02E-39	4.88E-38	80.0159055

ING3	-1.397334116	4.389200219	1.06E-39	5.07E-38	79.9751508
MYBBP1A	-1.077762253	4.876951828	1.12E-39	5.34E-38	79.9212578
MREG	-1.192236206	3.669902298	1.27E-39	6.00E-38	79.7936284
SPTY2D1	-1.942039672	4.621466084	1.35E-39	6.34E-38	79.7353978
TMEM18	-1.167434271	4.937754843	1.41E-39	6.61E-38	79.6911318
MAPKAPK5-AS1	-1.091124778	5.153216763	1.41E-39	6.61E-38	79.6886975
C5orf30	-1.270715359	3.847354768	1.45E-39	6.77E-38	79.6619137
PRMT9	-1.701752663	4.144341896	1.50E-39	7.00E-38	79.6254932
TSEN54	-1.237675767	6.583143648	1.61E-39	7.46E-38	79.5589368
TRAF6	-1.446513451	5.604118061	1.66E-39	7.69E-38	79.5268908
AGGF1	-1.760585362	4.555530197	1.69E-39	7.82E-38	79.5072844
FNDC3B	-1.957545545	3.322340716	1.70E-39	7.85E-38	79.5013453
APCDD1L	1.379697198	5.836515626	1.74E-39	7.96E-38	79.4807142
LOC338620	-1.324531902	3.75711707	1.75E-39	8.03E-38	79.4705257
ZSCAN26	-1.983243917	4.491798299	1.84E-39	8.37E-38	79.4243068
ZC3H14	-1.250656669	4.706184994	1.94E-39	8.78E-38	79.3701019
RNGTT	-1.162501769	4.591749258	1.94E-39	8.79E-38	79.3670227
TMEM64	-1.688353142	3.67804888	2.11E-39	9.51E-38	79.2839757
LINC00526	-1.315163355	3.546554087	2.16E-39	9.70E-38	79.2622241
PLXDC1	-1.038452477	4.870389089	2.44E-39	1.09E-37	79.1392208
CHAD	-1.029604959	4.665721221	2.52E-39	1.12E-37	79.107386
ZNF33B	-1.193208742	3.625260725	2.60E-39	1.15E-37	79.075043
DDX46	-1.749859954	5.92095485	2.62E-39	1.16E-37	79.0693857
SEC23IP	-1.385513737	4.381307778	2.63E-39	1.16E-37	79.0633006
KIAA1654	-1.16288908	4.035815247	2.77E-39	1.22E-37	79.0127244
DHFR	-1.102104552	4.264795386	3.24E-39	1.42E-37	78.8573906
PPHLN1	-1.155959718	4.766346458	3.41E-39	1.49E-37	78.8058939
HIVEP1	-1.05541918	3.710864226	3.90E-39	1.70E-37	78.6700018
IQCF5-AS1	-1.240728864	3.881327456	3.94E-39	1.70E-37	78.6611026
RP11-391M1.4	-1.702707736	4.087821936	3.94E-39	1.70E-37	78.6602878
GMPS	-1.042540262	4.702935748	4.13E-39	1.78E-37	78.6131044
ZNF765	-1.547439279	4.205417593	4.36E-39	1.87E-37	78.5588732
DSN1	-1.857681568	4.133054479	4.55E-39	1.95E-37	78.5168755
ZNF502	-1.17267051	4.302167926	4.56E-39	1.95E-37	78.51325
KLHL36	-1.128906382	6.533105588	4.79E-39	2.04E-37	78.4646499
LOC101929465	-1.069882468	4.179024503	5.13E-39	2.18E-37	78.3970846
LGMN	-1.256492548	3.498008925	5.38E-39	2.28E-37	78.3495006
CYB5B	-1.007699397	4.276212797	6.27E-39	2.66E-37	78.1955102
ZNF585A	-1.061933503	3.974274673	6.72E-39	2.84E-37	78.1257018
ALKBH1	-1.146870562	4.81576248	7.20E-39	3.03E-37	78.0577776
KRIT1	-1.562787223	4.801984167	7.46E-39	3.14E-37	78.0211401
FXN	-1.126016009	4.296439067	7.93E-39	3.33E-37	77.9605949
PIGS	-1.108249221	7.085001958	8.00E-39	3.35E-37	77.9516293
NR2C1	-1.134286612	3.283771736	8.21E-39	3.43E-37	77.9263459
ZNF251	-1.076875918	4.035782697	8.42E-39	3.51E-37	77.899938
ZNF542P	-1.607050577	3.852468574	8.82E-39	3.67E-37	77.8535794
LOC100128108	-1.470868716	3.302706589	8.93E-39	3.70E-37	77.8419387
ZNF778	-1.034741311	3.359858392	9.77E-39	4.02E-37	77.7521056
MSS51	-1.580303842	3.516196351	9.81E-39	4.03E-37	77.7473256
ZFX	-1.354802263	3.641951668	1.06E-38	4.32E-37	77.6710486
KDM5C	-1.269086187	5.354577626	1.07E-38	4.37E-37	77.6563731
KIF16B	-1.074053976	3.251713204	1.11E-38	4.53E-37	77.6199772
MOCS2	-1.170523824	3.20562772	1.18E-38	4.78E-37	77.5642721
RP11-416I2.1	-1.154411501	3.371284365	1.49E-38	6.02E-37	77.3277326
HMBOX1	-1.024450771	5.787037329	1.65E-38	6.65E-37	77.2245246
INPP5E	-1.332979183	5.938737092	1.77E-38	7.09E-37	77.1565574
PLGLB2	-2.149836551	3.345046691	1.86E-38	7.39E-37	77.109411
EFTUD2	-1.029647799	6.173611169	1.91E-38	7.58E-37	77.0815714

KLHL18	-1.018690594	5.484426998	2.09E-38	8.29E-37	76.9907386
NAP1L2	-1.394269546	2.852825618	2.26E-38	8.90E-37	76.9138349
GOLGA1	-1.333564206	5.563826397	2.28E-38	8.96E-37	76.9051006
MFSD6L	-1.082588664	5.830627952	2.51E-38	9.86E-37	76.8074468
ETV3	-1.421352206	5.139307907	2.64E-38	1.04E-36	76.7570568
NUDCD2	-1.191410345	4.076312339	2.69E-38	1.05E-36	76.7369188
TMEM53	-1.121198134	5.029930607	2.69E-38	1.05E-36	76.7365022
C5orf51	-1.110424407	4.529722973	2.74E-38	1.07E-36	76.7193613
XKRX	-1.150785835	3.530420702	2.83E-38	1.10E-36	76.6871693
LOC284513	-1.438828885	3.958106021	2.86E-38	1.11E-36	76.6759523
WHAMM	-1.622969135	5.238905894	3.00E-38	1.16E-36	76.6277966
ARF5	-1.340650779	7.680710386	3.15E-38	1.22E-36	76.5803476
LOC100293211	-1.0077484	3.487104793	3.39E-38	1.30E-36	76.5076782
EXTL2	-1.32696451	3.99787373	3.39E-38	1.30E-36	76.507399
SHQ1	-1.717687872	4.640454359	3.79E-38	1.45E-36	76.394183
USP44	-1.236414982	2.970832139	3.81E-38	1.45E-36	76.3906228
SOCS2	-1.184844448	3.632786819	4.23E-38	1.61E-36	76.2836463
RP5-1092A3.4	-1.521544513	3.123517548	4.29E-38	1.63E-36	76.2697203
RBM26	-1.20352531	4.359567642	4.36E-38	1.65E-36	76.2533996
PRUNE	-1.308550905	5.088043279	4.41E-38	1.67E-36	76.2427211
UBE2NL	-1.171393018	7.044432714	5.11E-38	1.92E-36	76.0947609
C10orf85	-1.435573888	4.247049266	5.30E-38	1.98E-36	76.0585144
LOC102723479	-1.046386366	3.315992043	5.46E-38	2.03E-36	76.0301961
LOC101930114	-1.131865851	3.899961358	5.54E-38	2.06E-36	76.0152244
CCDC104	-1.238533275	3.549327347	5.57E-38	2.06E-36	76.0095994
ZNF468	-2.48983419	3.774732544	5.63E-38	2.08E-36	75.9986944
RFC3	-1.034735999	3.304512721	5.74E-38	2.12E-36	75.9786957
RNASEH1	-1.302893781	5.761008918	6.13E-38	2.26E-36	75.9127514
PLCXD2	-1.136477727	4.122074786	6.18E-38	2.27E-36	75.9059155
FCF1	-1.279400725	3.806703401	6.24E-38	2.29E-36	75.896233
AKTIP	-1.366818185	5.647463111	6.44E-38	2.36E-36	75.8636792
MRPL30	-1.110092503	5.09224937	6.55E-38	2.40E-36	75.8466268
TBCCD1	-1.593795725	3.794471044	6.69E-38	2.44E-36	75.8264575
ANKRD33	-1.209902776	3.7306681	6.73E-38	2.45E-36	75.8196028
LINC00674	-1.146485916	4.840077022	6.79E-38	2.47E-36	75.8105242
TPT1-AS1	-1.024962378	4.313439691	7.07E-38	2.57E-36	75.7710111
USP24	-1.585720803	5.475157979	7.18E-38	2.60E-36	75.7553936
GPR19	-1.448726658	3.922199787	7.65E-38	2.77E-36	75.6914091
ZIC5	1.38691783	3.806607098	8.15E-38	2.93E-36	75.6277929
FAM122A	-1.19192369	5.174572382	8.25E-38	2.96E-36	75.6165714
SFXN1	-1.632574305	4.821450613	8.57E-38	3.06E-36	75.5780259
ATP6V0A2	-1.112135299	4.519681776	9.33E-38	3.31E-36	75.4926309
RNF167	-1.197028691	7.390265597	9.38E-38	3.33E-36	75.4872122
SMPDL3A	-1.174070053	3.504184541	9.51E-38	3.35E-36	75.4741105
PLEKHA8P1	-1.268817452	5.016233957	1.06E-37	3.71E-36	75.3670578
CENPH	-1.435684403	3.081599255	1.13E-37	3.95E-36	75.2984352
PRICKLE3	-1.373540929	4.39505611	1.25E-37	4.35E-36	75.197466
LOC101929684	-1.2248585	3.65899998	1.30E-37	4.51E-36	75.1590684
SCAPER	-1.332953497	4.424682367	1.42E-37	4.91E-36	75.0702346
LACTB2	-1.693838233	3.658179731	1.42E-37	4.91E-36	75.0689548
RFXAP	-1.336024541	3.830549879	1.43E-37	4.91E-36	75.068104
MPHOSPH9	-1.653310658	3.916280217	1.45E-37	4.95E-36	75.0542835
MYO9B	-1.075668389	6.697832919	1.46E-37	5.00E-36	75.044204
TAF4B	-1.268437229	3.751003793	1.47E-37	5.03E-36	75.0360124
SGMS2	-1.452670095	3.689520475	1.50E-37	5.10E-36	75.0189984
CBLN2	-1.145518211	3.761004645	1.50E-37	5.10E-36	75.0188647
AP1B1	1.141013031	6.541551123	1.50E-37	5.10E-36	75.0158349
IL1RAP	-1.732589274	4.620055411	1.53E-37	5.20E-36	74.9949902

MYZAP	-1.562514293	4.517246305	1.59E-37	5.39E-36	74.9562258
FAM124B	-1.172897044	3.660271204	1.61E-37	5.43E-36	74.946393
TRPS1	-1.00181525	4.952558469	1.67E-37	5.63E-36	74.9097815
EP300	-2.486411628	5.333328378	1.69E-37	5.69E-36	74.8968042
TRRAP	-1.76419314	5.097815919	1.88E-37	6.28E-36	74.7908241
INPP5B	-1.222924945	4.632915352	1.90E-37	6.33E-36	74.7813824
LOC285147	-1.818059662	3.664489975	1.92E-37	6.40E-36	74.7684666
RP11-109G23.3	-1.179193383	4.297204161	1.96E-37	6.51E-36	74.7478653
WDR53	-1.409743174	3.784607074	2.03E-37	6.72E-36	74.7146827
RIC8B	-1.013297105	4.862891483	2.16E-37	7.13E-36	74.6547175
RNF170	-1.35200183	4.68888035	2.18E-37	7.19E-36	74.6421602
STRN	-1.283550171	4.85524012	2.42E-37	7.95E-36	74.5386231
NAPEPLD	-1.148894321	4.291845831	2.44E-37	8.01E-36	74.5302264
C15orf54	-1.295565262	3.032588362	2.47E-37	8.10E-36	74.5173054
FUT11	-1.514539676	5.431420444	2.55E-37	8.35E-36	74.4858604
SLC35B4	-1.111097299	4.739318645	2.59E-37	8.45E-36	74.4714055
PAICS	-1.162333244	4.200830183	2.85E-37	9.27E-36	74.3751437
PAN3-AS1	-1.726891933	4.385930737	2.93E-37	9.51E-36	74.3462573
C20orf194	-1.074833982	5.743525855	2.96E-37	9.59E-36	74.3362982
MRPS11	-1.308855167	5.657212702	2.98E-37	9.64E-36	74.3293564
PROSER1	-2.089529848	4.518139295	3.15E-37	1.02E-35	74.2760457
PPP2R2B	-1.097300024	3.493716147	3.16E-37	1.02E-35	74.2728685
TNRC6A	-1.077674146	5.615936068	3.42E-37	1.10E-35	74.1913369
ZMAT4	-1.134194486	5.011375169	3.62E-37	1.15E-35	74.1353087
PPP3CB-AS1	-1.213047235	4.885991181	3.69E-37	1.17E-35	74.1171013
REST	-1.4256304	5.791704841	3.73E-37	1.18E-35	74.1054702
GK5	-1.111241211	2.743685456	3.77E-37	1.19E-35	74.0958072
PATL1	-1.042588651	5.367659851	3.89E-37	1.23E-35	74.0640625
INPP5A	-1.283483621	5.757581782	3.91E-37	1.23E-35	74.0588176
ZNF57	-1.32587314	3.573030449	3.92E-37	1.23E-35	74.0566439
VPS41	-1.38842716	4.735232874	3.92E-37	1.24E-35	74.0548819
RP11-73M18.8	-1.348474286	6.808531107	3.98E-37	1.25E-35	74.041612
ZW10	-1.44315096	5.321358995	4.00E-37	1.25E-35	74.0364136
TPD52	-1.822252092	3.777205247	4.15E-37	1.30E-35	73.9976072
CTBP1	-1.465976402	7.008781573	4.19E-37	1.31E-35	73.9882341
RMDN1	-1.448354549	4.252727313	4.22E-37	1.32E-35	73.9817523
C12orf66	-1.488488553	3.642947612	4.29E-37	1.34E-35	73.9655506
MOGAT1	-1.159740674	4.209931217	4.29E-37	1.34E-35	73.9651319
OSGEPL1	-1.71220642	4.596429371	4.38E-37	1.36E-35	73.9439021
SMS	-2.653231056	4.463611066	4.39E-37	1.36E-35	73.9434698
NUP133	-1.376944113	5.295979305	4.41E-37	1.37E-35	73.9383554
HSF2	-1.430854151	2.877671154	4.55E-37	1.41E-35	73.9056159
SYTL2	-2.05805143	4.933476765	4.64E-37	1.43E-35	73.8867392
ESYT2	-1.847115515	4.766086166	4.69E-37	1.44E-35	73.8766587
C2orf43	-1.319278145	4.969377728	4.80E-37	1.47E-35	73.8529782
PEX12	-1.353264954	5.172954888	5.40E-37	1.65E-35	73.7351013
ADPGK	-1.545184383	6.581428952	5.43E-37	1.66E-35	73.7290399
SNAPC3	-1.474822874	3.921798216	5.59E-37	1.70E-35	73.7003843
RP11-355B11.2	-1.434662414	3.638667594	5.80E-37	1.77E-35	73.6628096
PCYT1A	-1.094007505	5.64041857	5.97E-37	1.81E-35	73.6343715
RP1-178F10.1	-1.263064528	3.739489145	6.10E-37	1.85E-35	73.6128668
C6orf57	-1.23407946	3.438762971	6.17E-37	1.87E-35	73.6014811
RP11-804H8.6	-1.102042522	4.855998397	7.20E-37	2.16E-35	73.4468784
MBTD1	-2.033596426	5.054903496	7.34E-37	2.20E-35	73.4278055
ZNF253	-1.195874497	3.997277498	7.36E-37	2.20E-35	73.4256278
ZNF189	-1.927979841	5.138421536	7.74E-37	2.31E-35	73.3750522
AGPAT4	-1.096332639	4.156562479	8.18E-37	2.44E-35	73.3194415
ITCH	-1.331436941	4.447100499	8.76E-37	2.60E-35	73.2509128

CCDC127	-1.126473398	4.844360125	8.77E-37	2.60E-35	73.2496459
TTC13	-1.914083846	6.194315304	8.82E-37	2.62E-35	73.2437499
TARBP1	-1.234595464	6.562687902	9.44E-37	2.80E-35	73.1760826
TMEM68	-1.028333284	3.233704521	1.09E-36	3.21E-35	73.0332443
MRPL19	-1.247697025	2.919953489	1.14E-36	3.36E-35	72.9862138
ASUN	-2.844363868	4.188514619	1.19E-36	3.49E-35	72.9452004
MAZ	-1.078727025	6.359393864	1.21E-36	3.56E-35	72.9246679
DIDO1	-1.14119777	5.714076636	1.23E-36	3.61E-35	72.9094883
STAT5A	-1.272122924	7.719538925	1.29E-36	3.75E-35	72.8661547
DUSP14	-1.089037953	4.286203432	1.30E-36	3.78E-35	72.8576795
DLAT	-1.735463129	3.881425135	1.32E-36	3.84E-35	72.8385222
CDCA7	-1.213786232	3.471851114	1.32E-36	3.84E-35	72.8382147
LOC151657	-1.47249218	5.478767089	1.52E-36	4.39E-35	72.6986957
ZNF26	-1.152719074	3.724776229	1.61E-36	4.64E-35	72.641109
LOC100129361	-1.121271109	4.220191931	1.72E-36	4.92E-35	72.5775339
FAM64A	-1.139023647	4.423841218	1.75E-36	5.01E-35	72.5575887
CRKL	-1.161536439	5.895435953	1.75E-36	5.01E-35	72.5571836
SH2B3	-2.021480064	6.77096542	1.83E-36	5.23E-35	72.5122929
TTC27	-1.132172308	5.119276381	1.83E-36	5.23E-35	72.5117787
PDCD2L	-1.194490891	4.572102024	1.92E-36	5.46E-35	72.4647486
LOC101928370	-1.432406352	3.392570081	2.15E-36	6.06E-35	72.3538227
MYPN	-1.12367438	4.153331224	2.15E-36	6.06E-35	72.3519137
STAM2	-1.375571266	4.426733337	2.21E-36	6.21E-35	72.325624
ADAD2	1.538688697	5.757645104	2.25E-36	6.33E-35	72.306361
MC4R	-1.025627372	3.227251611	2.30E-36	6.44E-35	72.28532
ZNF814	-1.554988055	3.788610579	2.31E-36	6.48E-35	72.2783866
SMCR8	-1.058484531	5.513096688	2.33E-36	6.52E-35	72.2712653
FAM213A	-1.176049755	4.949000274	2.40E-36	6.71E-35	72.2405727
RNFT1	-2.162912144	4.124352859	2.49E-36	6.94E-35	72.2052458
SEC14L1P1	-1.229342271	3.368657945	2.63E-36	7.33E-35	72.1492343
TCEAL1	-1.4017831	3.6247124	2.65E-36	7.38E-35	72.1423596
BOLA1	-1.250120121	3.929695766	2.78E-36	7.70E-35	72.0960185
MEST	-1.8463567	3.575806897	2.95E-36	8.16E-35	72.0347749
GTF3C3	-1.301388259	3.669254555	3.07E-36	8.47E-35	71.9965218
AGPAT5	-1.886288432	4.026051151	3.26E-36	8.99E-35	71.9344058
LRRC57	-1.40763294	4.201746334	3.28E-36	9.03E-35	71.9277764
KLHL20	-1.099406421	4.326595927	3.30E-36	9.07E-35	71.9221129
PRMT6	-1.638694753	3.255183285	3.44E-36	9.43E-35	71.8813862
LIMS1	-1.441587483	4.245645009	3.46E-36	9.47E-35	71.8758915
RBM28	-1.531039012	5.36956043	3.58E-36	9.78E-35	71.8419254
CCT6B	-1.030907142	3.585046883	3.74E-36	1.02E-34	71.7974306
DCUN1D4	-1.424196186	3.7610798	4.30E-36	1.17E-34	71.6583311
GLUD1	-1.943656014	5.689223964	4.31E-36	1.17E-34	71.6557117
C1orf204	-1.063250908	4.610571222	4.67E-36	1.26E-34	71.5748551
PRKCI	-1.201947338	4.296399085	4.95E-36	1.34E-34	71.517538
STK25	-1.600679007	5.791593251	5.10E-36	1.37E-34	71.4872089
RACGAP1	-2.091305842	4.034123829	5.19E-36	1.40E-34	71.4701387
ARL13B	-1.233403695	2.643703267	5.34E-36	1.44E-34	71.441397
UBE2Q1	-1.02817917	5.239185772	5.60E-36	1.50E-34	71.3932847
ZNF320	-1.512709623	3.613615258	5.80E-36	1.55E-34	71.3582706
HIST1H4E	-1.381384746	4.346194369	5.87E-36	1.57E-34	71.3457307
ZCCHC11	-1.485539444	4.643080456	6.10E-36	1.62E-34	71.3075042
SREK1	-1.159878556	3.90571058	6.17E-36	1.64E-34	71.2964298
ASXL2	-1.084730727	5.645214733	6.52E-36	1.73E-34	71.2413439
FAM89B	-1.124093991	7.756153873	6.60E-36	1.75E-34	71.2292785
CPT2	-1.353798877	5.062733448	6.68E-36	1.77E-34	71.2169892
C15orf57	-1.190451213	4.927738619	6.85E-36	1.81E-34	71.1912418
MUC13	1.039025141	4.564794838	7.24E-36	1.91E-34	71.1354179

F2R	-1.480666208	5.250823981	7.63E-36	2.01E-34	71.0828132
ARSG	-1.296257832	5.166708538	7.66E-36	2.01E-34	71.0797046
CLASP2	-1.21792016	4.360122519	8.37E-36	2.19E-34	70.9909134
CHN2	-1.003252255	3.884687803	8.64E-36	2.26E-34	70.9587858
TCF12	-2.053550916	4.281270742	8.70E-36	2.27E-34	70.9521243
KRTAP1-1	-1.064862533	3.535555116	8.84E-36	2.31E-34	70.9357838
DLG1	-1.283108517	4.306067743	8.86E-36	2.31E-34	70.9338364
ARL6IP6	-1.641549112	4.587813475	8.88E-36	2.31E-34	70.930962
PTBP2	-1.239603803	2.767480207	9.25E-36	2.40E-34	70.8907558
TXLNG	-1.336026868	4.453350814	9.34E-36	2.43E-34	70.8803128
GLCE	-1.164555733	2.881171543	9.50E-36	2.46E-34	70.8633488
TMEM44-AS1	-1.066822696	4.284397997	9.51E-36	2.46E-34	70.8629366
FAM204A	-1.131549649	4.375880064	1.00E-35	2.60E-34	70.8078309
CTPS1	-1.2417256	4.47745555	1.04E-35	2.70E-34	70.7685661
MARS2	-1.181670949	4.336640591	1.08E-35	2.78E-34	70.7359578
LOC101929668	-1.372810779	5.806663464	1.13E-35	2.91E-34	70.6874847
GMIP	-1.246532193	6.531442052	1.14E-35	2.92E-34	70.683368
LOC102606465	-1.873503298	3.855165897	1.15E-35	2.94E-34	70.6741452
KIAA1324L	-1.049803795	3.827698338	1.20E-35	3.06E-34	70.6332693
TMEM209	-1.436506871	3.666698677	1.34E-35	3.42E-34	70.516529
PXN	-1.387686618	6.747614716	1.47E-35	3.74E-34	70.4232477
DZIP3	-1.107826398	4.306767653	1.49E-35	3.78E-34	70.4122116
ZNF526	-1.378017028	4.73484775	1.52E-35	3.84E-34	70.3928704
FCHSD1	-1.342908616	5.395570324	1.53E-35	3.87E-34	70.3852515
SEMA4B	-1.348748806	7.828562049	1.58E-35	3.99E-34	70.3517545
RP11-416N2.4	-1.044641863	3.674473251	1.59E-35	4.01E-34	70.3471206
NEK4	-1.351072737	3.698710506	1.65E-35	4.15E-34	70.3081398
C2orf44	-1.06327618	3.624706701	1.67E-35	4.19E-34	70.2970446
ZNF84	-1.442037461	4.60831097	1.74E-35	4.33E-34	70.2594705
RCN1	-1.27128433	3.39298474	1.74E-35	4.34E-34	70.2550963
TTC39B	-1.035541138	3.40615252	1.79E-35	4.45E-34	70.2292715
LOC283861	-1.122289438	3.655843452	2.01E-35	4.96E-34	70.1142782
GABPB1	-1.372788224	4.497131069	2.08E-35	5.13E-34	70.0795232
ENO1-AS1	-1.196853075	3.957018856	2.09E-35	5.15E-34	70.0723296
PDSS2	-1.164254098	4.776568762	2.14E-35	5.25E-34	70.0514503
PER2	-1.12893211	4.555068905	2.18E-35	5.34E-34	70.0340527
HDDC2	-1.868224931	4.594118312	2.29E-35	5.60E-34	69.9827213
ABCG8	-1.209289398	4.013832743	2.31E-35	5.65E-34	69.9729291
RP11-470M17.2	-1.03896422	3.776433799	2.39E-35	5.82E-34	69.9414752
DEFA5	-1.118328757	3.42484318	2.40E-35	5.85E-34	69.9337892
DPY19L4	-1.087020855	2.678451386	2.50E-35	6.08E-34	69.8931407
COX5A	-1.202010822	6.315125248	2.60E-35	6.31E-34	69.8539296
ZNF738	-1.00057062	2.805242138	2.61E-35	6.31E-34	69.8517064
TAF1L	-1.229652577	4.918515798	2.61E-35	6.31E-34	69.85127
ARRDC1	-1.093885689	5.78747353	2.72E-35	6.56E-34	69.8110154
STARD9	-1.144075456	3.63365033	2.81E-35	6.79E-34	69.7761216
SGSH	-1.07976664	6.436250474	2.84E-35	6.83E-34	69.7672441
SLFNL1	-1.038307535	4.187433269	2.85E-35	6.84E-34	69.7647915
ARFGEF2	-1.337247536	4.547148098	2.93E-35	7.03E-34	69.7359093
CALB2	1.144421232	5.448086552	3.01E-35	7.22E-34	69.7091847
TSPYL2	-1.145787385	7.27459118	3.03E-35	7.25E-34	69.7031666
ZNF512	-1.468071898	7.010931424	3.10E-35	7.40E-34	69.6801106
ZNF317	-1.208661453	4.85872491	3.12E-35	7.44E-34	69.6724772
GFM1	-1.501906003	4.096028099	3.14E-35	7.47E-34	69.66614
AP1G1	-1.434129422	6.179932448	3.25E-35	7.71E-34	69.6322772
LOC101929027	-1.18696754	3.587441317	3.35E-35	7.93E-34	69.602
DDX20	-1.38702158	4.108770186	3.50E-35	8.27E-34	69.5571093
C1orf159	-1.432233002	5.474987434	3.64E-35	8.60E-34	69.5172054

BHLHB9	-1.111668463	3.129004518	3.77E-35	8.88E-34	69.4829779
EAF1	-2.311077743	4.395739096	3.86E-35	9.07E-34	69.4593473
CCDC50	-1.245802783	4.087843607	4.05E-35	9.50E-34	69.4107992
UPF3A	-1.512535891	4.872535978	4.12E-35	9.65E-34	69.3941837
FBXO3	-1.075411082	3.164193066	4.55E-35	1.06E-33	69.2943016
ONECUT1	-1.134997172	3.64067389	4.77E-35	1.11E-33	69.2478072
TESK2	-1.15731821	6.400622728	5.21E-35	1.21E-33	69.1586365
MYSM1	-1.872152427	3.649713619	5.31E-35	1.23E-33	69.140235
RAB26	-1.051495193	4.617793264	5.35E-35	1.24E-33	69.1323913
TAF5	-1.861594528	4.120879589	5.49E-35	1.27E-33	69.1062087
TFCP2	-1.302916018	5.49915892	5.99E-35	1.38E-33	69.0194122
IFFO1	-1.286039795	6.96139793	6.31E-35	1.45E-33	68.9680667
CWC25	-1.114723184	6.018210928	6.54E-35	1.50E-33	68.931565
SLC7A6	-1.340353023	5.23623318	6.56E-35	1.50E-33	68.9281455
SAE1	-1.204948977	5.456743859	6.82E-35	1.56E-33	68.8892182
PHTF1	-1.103148065	4.060822307	7.10E-35	1.61E-33	68.8500137
NFX1	-1.213145111	5.024995236	7.14E-35	1.62E-33	68.8432371
ZWINT	-1.643365056	3.969474981	8.37E-35	1.90E-33	68.684179
RINT1	-2.323448737	3.652551214	8.58E-35	1.94E-33	68.6593566
HCG18	-1.00301433	5.33553579	8.66E-35	1.95E-33	68.6504797
DDX59	-1.037806351	5.174367851	9.27E-35	2.07E-33	68.5820062
RAD17	-1.387238605	5.279132424	9.61E-35	2.14E-33	68.5457118
PEX19	-1.045394427	5.826675262	9.87E-35	2.20E-33	68.5191297
LINC00909	-2.360662864	4.83750149	9.98E-35	2.22E-33	68.5083753
PPRC1	-1.550574076	5.25243495	1.02E-34	2.26E-33	68.4882454
AAR2	-1.163009695	6.513341768	1.03E-34	2.29E-33	68.4741364
PHF23	-1.241614444	6.898592249	1.04E-34	2.30E-33	68.4692457
TRIT1	-1.570871037	5.01424511	1.20E-34	2.65E-33	68.3227461
ATP6V1G2	-1.178145138	4.225237677	1.21E-34	2.66E-33	68.3191979
BCL7B	-1.074825959	6.034476204	1.21E-34	2.68E-33	68.3118276
ICOS	-1.407111183	4.191205427	1.24E-34	2.73E-33	68.2904372
AIFM1	-1.337837929	5.73928099	1.34E-34	2.94E-33	68.2146485
MIRLET7D	-1.603564685	4.81787672	1.35E-34	2.96E-33	68.2080469
COPB2	-1.118827844	5.011458266	1.37E-34	3.01E-33	68.1912106
HCFC2	-1.287294817	3.522260777	1.38E-34	3.02E-33	68.1862405
ADD1	-1.058036714	7.072337636	1.43E-34	3.12E-33	68.1495221
PAFAH2	-1.22344046	5.197467102	1.46E-34	3.18E-33	68.129455
CERS6	-1.126668817	4.07339195	1.56E-34	3.40E-33	68.0596068
SPINK5	-1.171420555	3.571120692	1.61E-34	3.50E-33	68.0318103
FNBP4	-1.443724446	5.606138838	1.66E-34	3.60E-33	68.0002791
RP4-773N10.4	-1.625430144	4.468898408	1.68E-34	3.65E-33	67.9857744
AKIRIN2	-1.566394414	5.934018002	1.73E-34	3.75E-33	67.9562095
CD28	-1.448996055	4.297997697	1.79E-34	3.88E-33	67.9214426
IL21R	-1.200700275	5.416619939	1.83E-34	3.96E-33	67.9000799
RP11-315F22.1	1.03635424	4.355736793	1.83E-34	3.96E-33	67.8987523
LCMT2	-1.251875974	4.439582943	1.84E-34	3.98E-33	67.8932771
RP11-285J16.1	-1.306971558	5.149443367	1.90E-34	4.09E-33	67.8638526
FAM86A	-1.355093066	5.026364344	1.90E-34	4.09E-33	67.8624432
SNRK-AS1	-1.010197795	3.899117031	1.91E-34	4.11E-33	67.85873
SSR3	-1.279698234	4.547161371	1.92E-34	4.13E-33	67.8509324
G3BP1	-1.115459006	5.47488742	1.95E-34	4.19E-33	67.835858
CLIP4	-1.739463075	4.079601383	2.20E-34	4.68E-33	67.7150996
UBA5	-1.312111582	3.986993207	2.22E-34	4.71E-33	67.7079758
ZSWIM5	-1.033612637	3.604627002	2.30E-34	4.87E-33	67.6717981
GOSR1	-1.111928319	5.123836598	2.32E-34	4.90E-33	67.663682
PRKG2	-1.131048745	3.891779006	2.34E-34	4.95E-33	67.6532068
PSME3	-1.218513658	6.39534866	2.50E-34	5.27E-33	67.5871793
C11orf21	-1.225797194	6.249988553	2.63E-34	5.52E-33	67.5383674

CACNG7	-1.018547439	3.959964754	2.69E-34	5.63E-33	67.5147666
PURA	-1.421126276	4.898223982	2.72E-34	5.68E-33	67.5032229
AK055981	-1.410727075	4.777020921	2.76E-34	5.76E-33	67.4893453
BHLHE40	-1.566511067	5.566967665	2.81E-34	5.85E-33	67.4715299
LILRA1	-1.380196957	6.506080465	2.81E-34	5.85E-33	67.471422
TPM4	-1.044542489	5.394433251	2.86E-34	5.93E-33	67.4545289
ACPP	-1.021827246	4.09906814	3.11E-34	6.43E-33	67.3705818
RP1-39G22.7	-1.062736174	4.266283581	3.24E-34	6.68E-33	67.3299911
DGKQ	-1.311042099	5.869371226	3.29E-34	6.78E-33	67.3140647
GPC3	-1.01375063	3.704340576	3.48E-34	7.16E-33	67.2562732
TMEM19	-1.310996079	4.680102903	3.61E-34	7.42E-33	67.2197549
PHF2	-1.216179324	4.815119067	3.64E-34	7.46E-33	67.2126567
ZNF439	-1.577842809	3.065057344	3.82E-34	7.82E-33	67.164483
FIRRE	-1.167983414	4.352701926	3.89E-34	7.97E-33	67.1451824
CEP76	-1.175846086	3.034184171	3.97E-34	8.12E-33	67.1256486
ZNF304	-1.723377319	3.075497061	4.00E-34	8.17E-33	67.1184316
MEF2A	-1.934167507	4.696206766	4.14E-34	8.42E-33	67.0844898
POLE	-1.084315178	5.602639192	4.14E-34	8.43E-33	67.0822793
SLC44A1	-1.071305935	4.404906269	4.51E-34	9.17E-33	66.9966732
REPS1	-1.288891599	4.876493994	4.65E-34	9.43E-33	66.9679359
APH1A	-1.057529583	5.407302142	5.02E-34	1.02E-32	66.8900029
PIK3IP1	-1.56567622	7.136529754	5.04E-34	1.02E-32	66.8858746
CANT1	-1.240974011	7.319198691	5.16E-34	1.04E-32	66.8627494
ARHGDIA	-1.131609741	6.222095244	5.19E-34	1.05E-32	66.8565686
TBC1D19	-1.057948479	3.990140157	5.24E-34	1.05E-32	66.8482026
MORC4	-1.346294389	3.673333505	5.24E-34	1.05E-32	66.8472749
RMND1	-1.137817864	4.414190474	5.38E-34	1.08E-32	66.821013
OS9	-1.295437372	8.469558326	5.40E-34	1.08E-32	66.8178823
TAAR8	-1.042694239	3.871761334	5.96E-34	1.19E-32	66.7190189
ZMYM5	-1.062515587	3.540028721	6.88E-34	1.37E-32	66.5750389
GPALPP1	-1.703208263	4.387193097	7.16E-34	1.42E-32	66.5347425
C6orf136	-1.446162917	5.747715968	7.35E-34	1.46E-32	66.5087461
FUBP3	-2.147032993	5.15618527	7.77E-34	1.53E-32	66.4529866
MYO9A	-1.142096037	4.92339607	7.83E-34	1.55E-32	66.4443473
BUB1B	-1.05208508	2.996136344	8.13E-34	1.60E-32	66.4074415
CKAP5	-1.094298092	5.924186861	8.46E-34	1.67E-32	66.3673292
CIPC	-1.725610094	3.558574256	8.69E-34	1.71E-32	66.3408384
KLF11	-1.163370071	4.431091252	8.90E-34	1.74E-32	66.3168361
SMC2	-1.117722485	3.041564262	9.01E-34	1.76E-32	66.3042449
SAAL1	-1.200531111	5.057395018	9.28E-34	1.80E-32	66.2743322
CD226	-1.406391274	3.702036576	9.33E-34	1.81E-32	66.269034
RP11-127B20.2	-1.936558554	3.697948209	9.46E-34	1.83E-32	66.2550298
ZNF567	-1.108533854	3.695055339	9.48E-34	1.84E-32	66.2531274
ZNF529	-1.407340853	4.222154524	9.57E-34	1.85E-32	66.2438585
ZNHIT6	-1.759647161	3.693961908	9.73E-34	1.88E-32	66.2274938
TUBGCP3	-1.563279676	4.410803206	9.92E-34	1.91E-32	66.2084358
C3orf17	-1.43902456	5.221861518	1.03E-33	1.98E-32	66.1712478
RFC1	-1.630721329	5.183983369	1.03E-33	1.98E-32	66.1697678
STYX	-1.848942944	4.295618426	1.07E-33	2.06E-32	66.1297667
CCT5	-1.281329853	6.036567583	1.09E-33	2.09E-32	66.1108095
CKMT2-AS1	-1.392758882	4.277398176	1.23E-33	2.35E-32	65.9906352
AK2	-1.071941772	5.966601358	1.25E-33	2.37E-32	65.980273
ARPC4	-1.036250237	7.774826367	1.26E-33	2.41E-32	65.9653573
METTL13	-1.080745361	5.98411069	1.28E-33	2.44E-32	65.9493355
PMS2P3	-1.084894492	5.526592895	1.35E-33	2.56E-32	65.901583
LEF1-AS1	-1.448998356	3.705997478	1.35E-33	2.57E-32	65.8967704
ABCB4	-1.120261176	3.511446997	1.46E-33	2.77E-32	65.8210324
IRF2	-2.212624336	7.496206923	1.50E-33	2.83E-32	65.7969057

TRIM68	-1.568226367	5.422406467	1.62E-33	3.06E-32	65.7151525
SLC17A5	-1.326753683	5.889846596	1.77E-33	3.32E-32	65.6287113
CTD-2035E11.5	-1.356805174	3.258691349	1.79E-33	3.35E-32	65.6172941
ZC3H13	-1.248861926	5.52579815	1.79E-33	3.36E-32	65.6149968
FLJ45513	-1.338753927	5.204839665	1.86E-33	3.47E-32	65.5799261
KDELR1	-1.125509041	6.90905325	1.88E-33	3.50E-32	65.5695229
SUMF2	-1.336949744	6.197268997	1.95E-33	3.62E-32	65.5306459
FAM159A	-1.144496388	5.229119005	1.97E-33	3.65E-32	65.5210847
ERMN	-1.603847742	3.318939684	1.98E-33	3.67E-32	65.5154115
GTPBP4	-1.340220363	5.204820216	2.00E-33	3.70E-32	65.5062641
RCHY1	-1.632382848	3.668585807	2.01E-33	3.72E-32	65.4991652
TNFRSF10B	-1.06246541	5.552091944	2.05E-33	3.79E-32	65.4785849
TMEM161B	-1.234022283	4.539132948	2.06E-33	3.80E-32	65.4753796
STRN3	-1.529565759	3.789017469	2.09E-33	3.85E-32	65.4604506
NPC1	-1.09109028	4.478989465	2.10E-33	3.86E-32	65.4565694
ZNF763	-2.049267987	4.154623903	2.16E-33	3.98E-32	65.4266376
ATP2C1	-1.146635282	4.46568307	2.28E-33	4.17E-32	65.376081
KCTD6	-1.387495713	4.28477198	2.30E-33	4.20E-32	65.3677489
NCSTN	-1.234693809	5.624303278	2.34E-33	4.28E-32	65.3465341
DQ570835	-2.095833474	3.925039825	2.37E-33	4.32E-32	65.3368493
CUL2	-1.332382024	4.049954274	2.44E-33	4.45E-32	65.3057926
DDX19A	-1.095993348	4.789468621	2.45E-33	4.47E-32	65.3004792
MED15	-1.170084275	7.461758036	2.73E-33	4.96E-32	65.1938974
NDUFAF4	-1.171886105	4.402546267	2.88E-33	5.22E-32	65.1389683
KLHL12	-1.658117055	5.263780474	2.92E-33	5.27E-32	65.1275669
CD44	-1.132597358	6.335833919	3.32E-33	5.98E-32	64.9992501
CALCOCO1	-1.073504407	7.774171534	3.32E-33	5.99E-32	64.9969071
CERS2	-1.481941069	8.214535192	3.51E-33	6.31E-32	64.9417977
FAM193B	-1.05237363	5.894955349	3.53E-33	6.33E-32	64.9377557
POLK	-1.947516562	4.625128971	3.56E-33	6.38E-32	64.9286492
ADNP2	-1.089020222	6.255442151	3.58E-33	6.40E-32	64.923517
BRCC3	-1.456582869	3.926186564	3.62E-33	6.48E-32	64.9117179
ABHD15	-1.238251858	4.524961129	3.65E-33	6.53E-32	64.9030256
EPB41L4A-AS1	-1.35286741	5.084340303	3.81E-33	6.80E-32	64.8589865
GLIDR	-1.004659018	5.555481128	3.92E-33	6.99E-32	64.830759
MFSD8	-1.671321569	3.893024516	4.00E-33	7.12E-32	64.8107316
DDHD1	-1.283237248	4.382134391	4.09E-33	7.26E-32	64.7883921
LOC389199	1.13885588	6.433670398	4.21E-33	7.47E-32	64.7590641
ZNF2	-1.095163674	4.414848619	4.24E-33	7.50E-32	64.7536147
MESDC1	-1.238510114	6.441209603	4.24E-33	7.50E-32	64.7531555
MTFMT	-1.332264448	3.702139417	4.25E-33	7.51E-32	64.7502883
METTL14	-1.336120913	4.589235619	4.75E-33	8.36E-32	64.6386827
CCDC88C	-1.567079656	6.003000928	4.80E-33	8.43E-32	64.6289997
PNRC1	-2.005321484	8.74551689	4.86E-33	8.54E-32	64.6152491
LOC100505920	-1.126558595	3.501428721	5.04E-33	8.85E-32	64.5788002
BAG5	-1.284191613	5.244061121	5.13E-33	8.98E-32	64.5626694
GIMAP8	-1.433544092	5.592058375	5.14E-33	8.99E-32	64.5604403
GPATCH2L	-1.169591036	4.97255507	5.14E-33	8.99E-32	64.5596169
PPP2R1A	-1.008093677	6.977077241	5.22E-33	9.12E-32	64.543735
C22orf39	-1.467504306	4.927165676	5.35E-33	9.33E-32	64.5206041
C1orf131	-2.282631436	5.065346549	5.51E-33	9.61E-32	64.4900501
C2orf54	-1.139761827	4.911824571	5.59E-33	9.74E-32	64.4762427
SCIMP	-1.132614886	3.817367621	5.64E-33	9.82E-32	64.4668718
C9orf69	-1.389852594	6.82618186	5.75E-33	9.99E-32	64.4484818
CCDC117	-1.517198695	4.802343456	5.86E-33	1.02E-31	64.4294552
THOC2	-1.514692585	5.071172029	5.86E-33	1.02E-31	64.4288414
SMC6	-1.27830568	3.720888163	5.88E-33	1.02E-31	64.4250544
DFFB	-1.593520915	3.700609416	5.91E-33	1.02E-31	64.4209711

LHX5	1.199939033	5.295665003	6.07E-33	1.05E-31	64.3929662
RP3-327A19.5	-1.34518322	3.689594737	6.08E-33	1.05E-31	64.391671
TRNT1	-1.718882348	3.916884081	6.18E-33	1.06E-31	64.375153
PHF6	-1.395788427	3.72824376	6.22E-33	1.07E-31	64.3693779
DGKZ	-1.400347027	6.834532866	6.36E-33	1.09E-31	64.34679
HTR1D	-1.113088202	3.512570467	6.65E-33	1.14E-31	64.3026329
RP11-199F11.2	-1.59339793	4.488166941	6.66E-33	1.14E-31	64.3007858
MCOLN2	-1.269732094	4.895942653	6.69E-33	1.15E-31	64.296352
SGPP1	-2.950245478	3.665807503	6.69E-33	1.15E-31	64.2953552
TSPAN14	-1.135335558	7.481455143	6.84E-33	1.17E-31	64.2735169
DDX50	-1.123133739	5.569484088	6.92E-33	1.18E-31	64.2624469
GUF1	-1.177195246	3.499767089	6.99E-33	1.19E-31	64.252636
HNRNPU	-1.085296198	5.75445759	7.01E-33	1.20E-31	64.2493145
IMPACT	-1.344135974	2.87482128	7.12E-33	1.21E-31	64.233609
OGFOD1	-1.095229451	5.463576353	7.32E-33	1.25E-31	64.2053986
DBR1	-1.965042206	3.957747439	7.39E-33	1.26E-31	64.1968719
PJA1	-1.423907553	6.863588009	7.78E-33	1.32E-31	64.1442464
SOWAHC	-1.151636101	3.823739012	7.86E-33	1.33E-31	64.1350546
RBM4B	-1.394643943	6.046534874	8.06E-33	1.36E-31	64.1094713
LOC101927420	-1.005202426	4.591638695	8.20E-33	1.39E-31	64.0921719
TSPYL5	-1.008173595	3.600786016	8.31E-33	1.40E-31	64.079311
ALDH1A1	-2.081713112	4.424307084	8.35E-33	1.41E-31	64.0745474
UBXN8	-1.246482357	3.351609635	8.40E-33	1.42E-31	64.0681013
ZC3HC1	-1.066471511	5.93390253	8.56E-33	1.44E-31	64.0492922
TMCO6	-1.44499413	5.724507308	8.70E-33	1.46E-31	64.0326293
THAP11	-1.410029157	5.549190285	8.96E-33	1.50E-31	64.0029298
B3GNT2	-1.554506803	3.711877017	8.96E-33	1.50E-31	64.0028301
TUBD1	-1.145284463	4.522732046	8.97E-33	1.50E-31	64.0025287
EDEM2	-1.147407235	6.068487474	8.97E-33	1.50E-31	64.002182
ITGA6	-1.966311972	4.807491378	8.98E-33	1.50E-31	64.0013645
SNHG4	-1.038170374	2.911706644	9.02E-33	1.51E-31	63.9970899
CCDC58	-1.446090902	3.727692846	9.02E-33	1.51E-31	63.9960887
CD300LB	-1.061479681	6.577662351	9.32E-33	1.56E-31	63.9637355
LINC01165	1.000234504	3.89210556	9.53E-33	1.59E-31	63.9415384
ZBTB18	-1.996619607	5.580915283	9.74E-33	1.62E-31	63.919881
VCPIP1	-1.136598841	4.356616021	9.85E-33	1.64E-31	63.9082674
HDAC2	-1.416725938	4.702848529	1.06E-32	1.76E-31	63.8345489
RIOK2	-1.345223877	3.595539105	1.08E-32	1.79E-31	63.8164679
IREB2	-1.582379005	4.413808379	1.10E-32	1.82E-31	63.8012062
GXYLT1	-1.322959874	3.711002366	1.10E-32	1.82E-31	63.796514
CD81	-2.055276844	8.114371887	1.10E-32	1.82E-31	63.7953206
S1PR5	-1.228372045	5.538466266	1.10E-32	1.82E-31	63.7946467
ZXDC	-1.220808514	5.458642095	1.13E-32	1.86E-31	63.775219
RP11-1024P17.1	-1.140141352	3.166503317	1.17E-32	1.93E-31	63.7324353
AKAP1	-1.108946802	5.213099751	1.23E-32	2.02E-31	63.6852538
LOC157562	-1.193514847	5.160587805	1.26E-32	2.06E-31	63.6652912
SUPT16H	-1.368193085	6.579071582	1.33E-32	2.19E-31	63.6044965
FCRL3	-1.827408183	5.70562596	1.34E-32	2.19E-31	63.5993445
HCCAT5	-1.119984905	4.357182231	1.44E-32	2.36E-31	63.5261482
USP37	-1.101382355	4.011348882	1.45E-32	2.38E-31	63.518853
5-Mar	-1.243654164	4.713654436	1.45E-32	2.38E-31	63.5179238
TRPM6	-1.009249137	5.037556349	1.46E-32	2.38E-31	63.5155524
CMAS	-1.013944675	4.481946159	1.49E-32	2.43E-31	63.4925089
DAPK1	-1.427702899	4.968254549	1.50E-32	2.43E-31	63.4895566
TCERG1	-1.805526563	3.939542391	1.50E-32	2.44E-31	63.4862793
ZNF100	-1.150921663	3.17604759	1.54E-32	2.50E-31	63.4619014
TOX	-1.358758342	3.274093789	1.55E-32	2.51E-31	63.4570984
ITPR2	-1.235710879	4.527452345	1.72E-32	2.79E-31	63.3489362

METTL3	-1.301921117	6.10315396	1.73E-32	2.79E-31	63.3459735
LINC00202-1	1.332173516	5.696526247	1.77E-32	2.85E-31	63.3237844
LOC100289230	-1.485552172	4.725406182	1.90E-32	3.06E-31	63.2486943
LOC644794	-1.014650074	4.562393802	1.96E-32	3.16E-31	63.218099
ACLY	-1.035441702	6.290991735	2.08E-32	3.35E-31	63.1573427
ZNF516	-1.905035962	5.473641016	2.20E-32	3.53E-31	63.1043208
UBE2E2	-1.65855039	5.145598246	2.34E-32	3.74E-31	63.0421253
ALKBH2	-1.138771407	4.319639157	2.46E-32	3.93E-31	62.9920826
RRP36	-1.032412087	5.250569213	2.53E-32	4.04E-31	62.9630416
MIF4GD	-1.164490625	6.741331346	2.55E-32	4.07E-31	62.9564188
SUV39H1	-1.0632824	5.373410606	2.60E-32	4.15E-31	62.934348
FKTN	-1.665458801	4.306479398	2.61E-32	4.15E-31	62.9325559
APPBP2	-1.465761653	4.364909189	2.63E-32	4.19E-31	62.9242886
ZNF645	-1.082834859	3.702630275	2.67E-32	4.25E-31	62.9085009
ADK	-1.19718034	4.53282439	2.68E-32	4.25E-31	62.9074922
INTS6-AS1	-1.089509829	3.869453537	2.69E-32	4.27E-31	62.9028684
OSBPL3	-1.743477476	4.504362013	2.84E-32	4.50E-31	62.8482496
ANKRD10	-1.190169713	4.404658813	2.89E-32	4.57E-31	62.8311436
ZEB1-AS1	-1.344807626	3.110858381	2.94E-32	4.64E-31	62.8144821
ZNF639	-1.562705527	3.691629692	2.96E-32	4.68E-31	62.8047072
TRAPPC11	-1.612848973	3.995656814	3.01E-32	4.75E-31	62.7885994
UPF1	-1.206637542	6.266507297	3.18E-32	5.00E-31	62.7354909
C2orf49	-1.398401717	4.160012971	3.25E-32	5.10E-31	62.7119554
VPS37A	-1.87511278	4.17168315	3.52E-32	5.51E-31	62.6328856
AF090939	-1.313949725	4.57513702	3.61E-32	5.63E-31	62.6069548
JDP2	-1.01782455	5.041233053	3.72E-32	5.79E-31	62.5782592
ANGEL2	-1.661536837	4.187635283	3.79E-32	5.90E-31	62.5583089
C5orf24	-1.071272866	4.163785757	3.97E-32	6.16E-31	62.5127036
API5	-1.352653857	4.607887815	4.47E-32	6.92E-31	62.3934784
HPS3	-1.091339986	4.500967648	4.56E-32	7.06E-31	62.3721403
TRIM59	-1.632073695	3.135630625	4.65E-32	7.18E-31	62.3541873
PARN	-1.306430041	6.559136365	4.78E-32	7.36E-31	62.3261209
SUPT7L	-1.160176183	4.778205683	4.80E-32	7.38E-31	62.3223028
ZXDA	-1.083761362	3.521408943	4.97E-32	7.64E-31	62.2869699
TNIK	-1.022821934	5.07155739	5.11E-32	7.85E-31	62.2596243
MSH2	-1.795433635	3.730549582	5.17E-32	7.94E-31	62.2473631
R3HDM2	-1.133054646	6.883637918	5.28E-32	8.09E-31	62.2267085
PEX3	-1.27016937	4.176819146	5.35E-32	8.18E-31	62.2130719
AKAP17A	-1.237076566	6.110364755	5.35E-32	8.18E-31	62.2130699
METTL25	-1.439804431	5.420099782	5.44E-32	8.31E-31	62.1965839
RP11-33O4.1	-1.145494652	4.915958752	5.46E-32	8.33E-31	62.1935801
FNIP2	-1.559543099	5.227508894	5.51E-32	8.40E-31	62.1841867
PROSER3	-1.133027589	4.966301074	5.91E-32	9.00E-31	62.113059
LPIN1	-1.291369914	5.640843587	6.32E-32	9.59E-31	62.0455871
MTMR1	-1.431475598	5.652348321	6.34E-32	9.61E-31	62.0422545
FAM46A	-1.154593132	5.26387744	6.46E-32	9.77E-31	62.0238999
TCEAL8	-2.125780802	4.167005391	6.60E-32	9.95E-31	62.0031413
MAPKAPK5	-1.313187895	4.070939715	6.84E-32	1.03E-30	61.9667105
RP11-4M23.7	1.280707661	4.852415756	6.96E-32	1.04E-30	61.9502413
C1orf109	-1.929098333	3.1376422	7.08E-32	1.06E-30	61.9324081
NUP85	-1.486262355	5.856158342	7.12E-32	1.07E-30	61.92671
PALB2	-1.548368854	4.652708116	7.27E-32	1.09E-30	61.905833
KIZ	-1.328478204	5.147312444	7.29E-32	1.09E-30	61.9036923
TMEM39B	-1.161454212	5.470903625	7.30E-32	1.09E-30	61.9016218
SLC30A5	-1.507400798	4.157533805	7.36E-32	1.10E-30	61.8932272
PLEKHG1	-1.002848446	3.074453903	7.59E-32	1.13E-30	61.8628293
KLHL42	-1.157477042	4.652944441	7.62E-32	1.13E-30	61.8587832
SPRY4	-1.093633431	5.156624795	7.80E-32	1.16E-30	61.8353052

TMEM87A	-1.207088151	5.026915809	8.24E-32	1.22E-30	61.7802897
LDOC1	-1.340866068	5.014367379	8.51E-32	1.25E-30	61.7484497
S1PR1	-2.202375701	5.4746144	8.60E-32	1.27E-30	61.7378251
ATP6	-1.128527785	8.483743447	8.65E-32	1.27E-30	61.7316863
SIN3B	-1.042410812	5.97046024	8.79E-32	1.29E-30	61.715426
PANK4	-1.173301307	6.306783983	8.82E-32	1.30E-30	61.7128206
LOC286367	-1.024379098	3.474872639	8.96E-32	1.32E-30	61.6966674
SMARCC1	-1.2061216	6.012403514	9.36E-32	1.37E-30	61.6525924
ATF2	-1.503280678	3.407996766	9.86E-32	1.44E-30	61.6009016
BACH1	-1.381197079	5.211775596	9.95E-32	1.45E-30	61.591644
G2E3	-1.633602751	4.091230219	1.01E-31	1.47E-30	61.5794816
TFIP11	-1.00745478	5.662453113	1.02E-31	1.49E-30	61.5668499
ATAD2B	-1.448298821	3.882835637	1.02E-31	1.49E-30	61.5655756
CAND1	-1.389614352	4.553851032	1.06E-31	1.54E-30	61.530437
RFX5	-1.032838752	6.826701956	1.13E-31	1.65E-30	61.4604507
EXOC2	-1.458390028	4.889228996	1.14E-31	1.65E-30	61.4568946
SNX20	-1.204627708	5.890788041	1.21E-31	1.76E-30	61.3923811
MIR3682	-1.334443096	3.05002542	1.24E-31	1.80E-30	61.3696506
TC2N	-1.658924575	3.509008415	1.26E-31	1.82E-30	61.3581261
GLCCI1	-1.507511366	4.658446259	1.26E-31	1.83E-30	61.3526232
SRPRB	-1.025992406	5.538960349	1.29E-31	1.87E-30	61.3285472
CCNT2	-1.86892788	3.555929163	1.29E-31	1.87E-30	61.327806
AKNA	-1.258290629	6.038933559	1.30E-31	1.87E-30	61.3259841
C7orf49	-1.091799219	6.33624489	1.31E-31	1.89E-30	61.3164224
C11orf80	-1.284604819	4.562158546	1.47E-31	2.11E-30	61.2003922
NDUFS4	-1.173960712	4.664419388	1.58E-31	2.27E-30	61.1259238
LARS2	-1.061634212	4.746320995	1.62E-31	2.31E-30	61.1061413
RASSF1	-1.093978098	6.733909599	1.63E-31	2.32E-30	61.0992106
C19orf12	-1.354798507	5.818097577	1.65E-31	2.36E-30	61.0844661
ZNF75A	-1.072164069	5.701566061	1.68E-31	2.40E-30	61.065381
MFAP3	-1.089038297	4.650325934	1.71E-31	2.43E-30	61.0502883
VPS26A	-1.489391978	5.201579424	1.73E-31	2.46E-30	61.0397393
MAPK9	-1.259952421	4.900463299	1.79E-31	2.54E-30	61.0050781
SRSF1	-1.251268268	5.57440342	1.83E-31	2.60E-30	60.9810066
URI1	-1.209497785	4.26988465	1.88E-31	2.67E-30	60.9521748
HLA-J	-1.400859767	9.551544459	1.89E-31	2.68E-30	60.9467342
RHNO1	-1.246024439	4.000300118	1.90E-31	2.68E-30	60.944803
SLC25A33	-1.601074634	4.291451925	1.92E-31	2.71E-30	60.9338808
DNMBP	-1.069145433	5.263774226	1.92E-31	2.71E-30	60.9336294
LOC285957	-1.531297328	3.759682842	1.98E-31	2.80E-30	60.8997937
IRX3	-1.127722965	2.70735553	2.03E-31	2.86E-30	60.8788593
PRKAA1	-1.280941999	4.100589142	2.04E-31	2.88E-30	60.8700125
NAA40	-1.853133018	5.382509972	2.05E-31	2.88E-30	60.8689307
RALGAPB	-1.156043551	5.304321567	2.13E-31	2.99E-30	60.8296153
RGS14	-1.137883549	6.286046202	2.13E-31	2.99E-30	60.8286395
PRPF40B	-1.078825099	4.667670412	2.15E-31	3.02E-30	60.8180989
XAGE2	1.203106476	5.751648904	2.18E-31	3.06E-30	60.8053596
KANSL1L	-1.006519373	3.827883964	2.19E-31	3.07E-30	60.7995743
DDX55	-1.08247883	3.961704008	2.28E-31	3.19E-30	60.7592386
NSRP1	-1.523642571	4.919490793	2.34E-31	3.27E-30	60.7361548
SCAP	-1.252039433	7.562637981	2.35E-31	3.28E-30	60.7293813
ETAA1	-1.169007094	2.888338978	2.38E-31	3.32E-30	60.7163313
BC022047	-1.422948499	5.911436423	2.39E-31	3.33E-30	60.714876
AP000347.2	-1.512715156	4.300735102	2.52E-31	3.50E-30	60.659152
SYNRG	-1.287993938	6.172438736	2.66E-31	3.68E-30	60.6058863
PTPN18	-1.425147822	6.158804326	2.87E-31	3.97E-30	60.5290285
GCLM	-1.40766914	4.342830309	2.92E-31	4.03E-30	60.5115176
THTPA	-1.201139118	6.552409856	2.98E-31	4.10E-30	60.4935619

SMYD4	-1.141017989	5.841630665	2.98E-31	4.10E-30	60.4917441
LOC93622	-2.430996212	5.113669428	3.03E-31	4.16E-30	60.4764397
RUSC1	-1.274740608	6.193855765	3.06E-31	4.19E-30	60.467058
FAM193A	-1.583706621	6.317326861	3.11E-31	4.26E-30	60.450533
BC079832	-1.129658238	3.816930524	3.12E-31	4.27E-30	60.4467817
MED23	-1.362037963	4.835352005	3.14E-31	4.30E-30	60.4402799
ZADH2	-1.339116254	4.704889469	3.15E-31	4.31E-30	60.4358259
CRYL1	-1.036070801	5.879082193	3.20E-31	4.37E-30	60.419847
MSL3	-1.079705369	5.224380834	3.20E-31	4.37E-30	60.4197057
RB1	-1.959657051	4.202231227	3.28E-31	4.47E-30	60.3953081
AC100830.4	-1.099771713	4.238524527	3.36E-31	4.57E-30	60.37319
HNRNPAB	-1.464600123	7.311257158	3.38E-31	4.59E-30	60.3665375
RP11-394I13.2	-1.489880251	3.626239689	3.42E-31	4.64E-30	60.355125
GFPT1	-1.096665626	3.935560869	3.43E-31	4.65E-30	60.3513406
PLEKHA3	-1.144240461	4.488359354	3.49E-31	4.72E-30	60.335598
VAMP4	-1.346429312	4.065033754	3.49E-31	4.73E-30	60.3341518
METTL4	-1.552543247	4.249366769	3.61E-31	4.88E-30	60.3008265
PSMG1	-1.692086659	3.785990932	3.68E-31	4.98E-30	60.2800531
CHTOP	-1.091211381	6.866076425	3.69E-31	4.98E-30	60.2790359
LOC283588	-2.141141826	3.943306985	3.78E-31	5.10E-30	60.2548737
EIF4E	-1.088186853	4.087418887	3.80E-31	5.13E-30	60.2481198
ZNF665	-1.646471669	4.622004284	3.92E-31	5.28E-30	60.2180157
PAXBP1	-1.534355929	4.592584206	4.00E-31	5.38E-30	60.1983863
SLAIN1	-1.505801105	3.014102282	4.09E-31	5.49E-30	60.174894
CTDSPL2	-1.60980562	4.149504233	4.19E-31	5.62E-30	60.1520382
TRIM5	-1.369925051	4.259461398	4.46E-31	5.96E-30	60.089074
TM2D1	-1.560452772	4.756217771	4.48E-31	5.99E-30	60.0842287
PARP11	-1.08349258	4.129831436	4.63E-31	6.18E-30	60.0508493
TNF	-1.184772808	5.08549117	4.69E-31	6.26E-30	60.0373168
C14orf93	-1.154825485	4.14763276	4.72E-31	6.29E-30	60.0322188
PRDX3	-1.383318663	5.474635664	4.79E-31	6.37E-30	60.0177938
LOC102723692	-1.067636869	4.190320637	4.87E-31	6.48E-30	60.0007669
PAPOLG	-1.162453597	4.843663938	4.92E-31	6.54E-30	59.9895419
TWSG1	-1.136499199	3.192256561	4.93E-31	6.55E-30	59.9880811
EPHA5-AS1	1.254488683	5.348778841	5.13E-31	6.82E-30	59.9476588
TGDS	-1.188477466	4.389723836	5.18E-31	6.87E-30	59.9386104
FAM127A	-1.096945183	7.932051107	5.40E-31	7.14E-30	59.8976853
CIAPIN1	-1.176737115	5.437722226	5.70E-31	7.54E-30	59.84205
DUSP5	-1.505339367	4.349905297	5.78E-31	7.63E-30	59.8284494
C18orf21	-1.050774589	4.269939206	5.80E-31	7.65E-30	59.8256784
CLN5	-1.487086942	4.400078906	5.96E-31	7.86E-30	59.797835
TMEM184C	-1.129456385	6.491401013	5.98E-31	7.88E-30	59.7938873
BCORP1	-1.003346469	3.057936336	6.02E-31	7.93E-30	59.7873619
ACVR2A	-1.081892733	4.163168763	6.27E-31	8.24E-30	59.7477217
GATAD2B	-1.325209559	6.798326528	6.31E-31	8.29E-30	59.7411519
AGK	-1.013976505	4.068712872	6.42E-31	8.43E-30	59.723856
SYT11	-1.025285723	4.757024156	6.50E-31	8.52E-30	59.71171
NIP7	-1.464689949	5.242742747	6.56E-31	8.59E-30	59.7022974
CBLL1	-2.259969284	3.809905908	6.68E-31	8.74E-30	59.6836717
PEF1	-1.182425416	7.086990914	6.74E-31	8.81E-30	59.6750985
LINC-PINT	-1.081641209	4.713156938	7.41E-31	9.67E-30	59.5803479
FANCF	-1.25561511	4.954062089	7.51E-31	9.80E-30	59.5665976
WDR83OS	-1.093980753	8.420754081	7.59E-31	9.90E-30	59.5556962
RRS1	-1.519747221	6.080735618	7.70E-31	1.00E-29	59.5419384
PDE3B	-1.73909201	5.263661538	7.76E-31	1.01E-29	59.5333554
PM20D1	-1.047838189	3.353627199	7.79E-31	1.01E-29	59.5298714
FAM102B	-2.611017468	3.924479311	8.31E-31	1.08E-29	59.4652752
QDPR	-1.023097479	5.334743419	8.49E-31	1.10E-29	59.4439996

MCRS1	-1.112791295	6.862232986	8.58E-31	1.11E-29	59.4325159
ASB16-AS1	-1.111282613	4.826557236	8.81E-31	1.14E-29	59.4064071
PPP2CA	-1.626745294	5.184187914	8.90E-31	1.15E-29	59.3963022
TCEB1	-1.582894618	4.191804453	9.12E-31	1.18E-29	59.3719534
CXCL5	-1.288511517	2.953245351	9.27E-31	1.20E-29	59.3555243
KDM1B	-1.316582529	4.041399547	9.34E-31	1.21E-29	59.3480667
PREPL	-1.216948996	3.606315949	9.68E-31	1.24E-29	59.3125897
CELF6	-1.124856961	3.69006115	9.79E-31	1.26E-29	59.301038
LPPR2	-1.039353125	7.299692708	9.82E-31	1.26E-29	59.2980862
SLC35B3	-1.866794463	4.098619749	1.01E-30	1.29E-29	59.271128
LINC00266-1	-1.630638771	3.792674519	1.03E-30	1.32E-29	59.2524289
WBP11	-1.374643115	5.466420801	1.07E-30	1.37E-29	59.2079054
MAP3K14	-1.152852922	5.950618429	1.08E-30	1.38E-29	59.2003413
SLC41A1	-1.230979769	5.640744418	1.27E-30	1.62E-29	59.0403198
ATG2B	-1.143169197	4.176018715	1.28E-30	1.62E-29	59.0348936
ATP13A3	-1.627598133	4.4284366	1.30E-30	1.65E-29	59.0190909
RNF44	-2.043899144	7.958928052	1.35E-30	1.71E-29	58.9809583
CACYBP	-1.417372424	5.17979798	1.35E-30	1.72E-29	58.9759407
HACE1	-1.503772943	3.550661834	1.36E-30	1.72E-29	58.9723315
STX16	-1.208258662	6.944845864	1.37E-30	1.74E-29	58.9605735
SRSF5	-1.542915749	6.828943795	1.41E-30	1.78E-29	58.9386898
FAM199X	-1.590129459	4.551826446	1.53E-30	1.93E-29	58.8551635
C9orf64	-1.276162187	6.398518307	1.58E-30	1.99E-29	58.8203552
PSMA5	-1.21320626	5.303504702	1.61E-30	2.03E-29	58.8031573
TANGO6	-1.033277571	5.269686788	1.73E-30	2.17E-29	58.7298154
IDS	-1.020178472	5.705386188	1.75E-30	2.20E-29	58.7177056
CHMP1B	-1.523124361	6.490409268	1.77E-30	2.22E-29	58.7082171
HOOK3	-1.185798979	4.086207416	1.78E-30	2.23E-29	58.7025653
KLF7	-1.188219355	5.797872517	1.78E-30	2.23E-29	58.7006563
SCYL3	-1.716993859	4.353787138	1.81E-30	2.26E-29	58.6856087
MASTL	-1.14099665	2.889797055	1.94E-30	2.42E-29	58.6146803
RDH11	-1.064293713	5.173284572	1.96E-30	2.45E-29	58.6046063
HSPA14	-1.162758873	4.575933237	1.99E-30	2.49E-29	58.5888706
BTBD3	-1.290990322	3.265274396	2.03E-30	2.53E-29	58.5688151
RP11-112J3.16	-1.379747748	3.276803491	2.05E-30	2.55E-29	58.561409
ZNF556	-1.091529743	3.912894045	2.05E-30	2.55E-29	58.5601741
MON2	-1.502339042	4.440773659	2.10E-30	2.61E-29	58.5376534
GRAMD1C	-1.822217649	2.998732123	2.12E-30	2.63E-29	58.5258309
MCM3	-1.082400505	6.290417188	2.39E-30	2.95E-29	58.4076821
RFNG	-1.201865721	5.863261395	2.48E-30	3.06E-29	58.3686356
SETD1B	-1.219343741	8.305651513	2.56E-30	3.15E-29	58.3395314
WASF3	-1.431299481	3.187002853	2.69E-30	3.31E-29	58.2874817
DET1	-1.115674264	5.307523822	2.77E-30	3.41E-29	58.2570472
EML4	-1.168843813	6.102174208	2.99E-30	3.67E-29	58.1836774
RAB39B	-1.377429162	3.178366826	3.05E-30	3.74E-29	58.1631265
PM20D2	-1.581409049	3.580778331	3.12E-30	3.81E-29	58.1393091
BTF3L4	-1.069823119	4.438108711	3.13E-30	3.82E-29	58.1363223
MSANTD2	-2.20447631	4.757006445	3.18E-30	3.88E-29	58.1191385
SFT2D2	-1.292658014	6.149999481	3.28E-30	3.98E-29	58.089261
ZDHHC20	-1.427010393	4.549571001	3.30E-30	4.00E-29	58.0838474
RPS6KC1	-1.447168088	4.155654941	3.30E-30	4.01E-29	58.0819913
C12orf10	-1.266707959	6.883933592	3.46E-30	4.18E-29	58.037321
RPRD1A	-1.328806521	4.046923037	3.46E-30	4.18E-29	58.0372599
SLC43A3	-1.192112944	6.10358907	3.51E-30	4.24E-29	58.0216205
FAM105A	-1.132144143	4.649240669	3.65E-30	4.38E-29	57.9829478
ZNF780A	-1.320726904	3.134131773	3.73E-30	4.46E-29	57.9619431
TXNDC15	-1.24259497	6.026494037	3.88E-30	4.65E-29	57.9202945
EHBP1	-1.309951597	4.178267502	3.92E-30	4.69E-29	57.9096201

PHF10	-1.519702392	5.285542887	3.93E-30	4.70E-29	57.9082727
BRAT1	-1.00650996	6.549519431	3.97E-30	4.74E-29	57.8993268
XXYLT1	-1.009303171	4.944146629	4.02E-30	4.80E-29	57.8854287
FAM114A2	-1.39858433	5.005614444	4.02E-30	4.80E-29	57.8850883
TNFRSF8	-1.182720585	5.420039242	4.04E-30	4.81E-29	57.8810382
ADORA2B	-1.104847397	4.102118281	4.08E-30	4.86E-29	57.8699495
PIM3	-1.510326182	7.722933358	4.14E-30	4.92E-29	57.856882
SH2D1B	-2.053420498	4.213868335	4.19E-30	4.98E-29	57.8450048
PRR14	-1.201993547	6.534692599	4.24E-30	5.03E-29	57.8332519
SLC35E3	-1.06902746	5.632665319	4.25E-30	5.05E-29	57.8303493
UPRT	-1.689278759	4.055672367	4.28E-30	5.08E-29	57.8222962
SOCS5	-1.284972098	3.214056627	4.32E-30	5.12E-29	57.813204
ARID1A	-1.156220943	7.16999935	4.38E-30	5.18E-29	57.8003121
CBLB	-1.313581646	4.470837298	4.52E-30	5.35E-29	57.7676243
C15orf40	-1.210569169	3.727375663	4.53E-30	5.35E-29	57.7654269
TSEN15	-1.105737173	5.088526939	4.59E-30	5.41E-29	57.7537236
HERC4	-1.232270554	3.807079169	4.70E-30	5.54E-29	57.7295681
NSUN4	-1.025188591	4.784309189	4.79E-30	5.64E-29	57.7099264
TDRD12	-1.015825287	3.770410475	4.86E-30	5.72E-29	57.6962782
TM9SF1	-1.127164584	5.064096191	5.18E-30	6.09E-29	57.6316933
YIPF3	-1.131936522	7.367593552	5.21E-30	6.13E-29	57.6257622
KLHL28	-1.626991925	6.04152617	5.24E-30	6.16E-29	57.6195879
AK097370	-1.103932885	4.686587096	5.34E-30	6.27E-29	57.6003696
CD300C	-1.047112874	6.646303928	5.38E-30	6.30E-29	57.5940564
SLC38A6	-1.304575435	3.266301427	5.44E-30	6.35E-29	57.5829847
BMS1P5	-1.605079384	4.423257664	5.47E-30	6.39E-29	57.5766773
RNF2	-1.189500273	3.687503847	5.53E-30	6.45E-29	57.5664145
RP11-649A18.7	-1.077508527	3.837156409	5.58E-30	6.49E-29	57.5575742
DNAJA3	-1.231210407	6.732632613	5.62E-30	6.54E-29	57.5499995
PSMD11	-1.176058472	6.250119525	5.72E-30	6.65E-29	57.5314747
GYS1	-1.119354317	6.609354903	6.00E-30	6.96E-29	57.4838593
CLCN6	-1.075758623	5.485144861	6.23E-30	7.21E-29	57.4459281
LINC01125	-1.147755519	4.130661896	6.32E-30	7.31E-29	57.4323989
TMEM55B	-1.293792775	6.830017996	6.33E-30	7.32E-29	57.4304274
DNPEP	-1.204835581	5.990955918	6.36E-30	7.35E-29	57.4253108
LINC01029	-1.029548404	3.915851819	6.46E-30	7.45E-29	57.4103557
PDS5A	-1.617639882	4.786753637	6.58E-30	7.58E-29	57.3926475
SLC13A4	1.045306949	5.580870383	6.62E-30	7.62E-29	57.3860373
CD200	-1.567636653	3.75401041	7.16E-30	8.21E-29	57.3077934
RP11-680G24.5	-1.261818866	4.012710287	7.31E-30	8.38E-29	57.2860163
HAPLN4	-1.084040989	4.384833273	7.34E-30	8.40E-29	57.2826266
ZNF786	-1.1831795	4.314095894	7.43E-30	8.50E-29	57.2705381
PDIK1L	-1.357106249	3.190940397	7.71E-30	8.78E-29	57.2336209
DDOST	-1.497857823	8.226656676	7.90E-30	8.99E-29	57.2089178
NUS1	-1.578640878	4.807592229	7.91E-30	9.00E-29	57.2074049
NUP35	-1.651699374	4.379898529	8.28E-30	9.40E-29	57.1621328
BIRC3	-2.038448966	5.227362782	8.32E-30	9.44E-29	57.1566631
SMAGP	-1.07107981	5.419798035	8.43E-30	9.56E-29	57.1439156
TTN-AS1	-1.323436849	4.744210531	8.46E-30	9.59E-29	57.1399764
PIK3C2A	-1.143783226	4.62871374	9.30E-30	1.05E-28	57.0452787
RBM48	-1.029073663	4.151452209	9.40E-30	1.06E-28	57.0344097
TAF2	-1.279156215	5.337014642	9.47E-30	1.07E-28	57.0268072
RAD54L	-1.09047081	4.458666021	9.89E-30	1.11E-28	56.9836963
CSE1L	-1.359100951	5.153228388	1.00E-29	1.12E-28	56.9718939
MTMR3	-1.020331856	6.579028763	1.04E-29	1.17E-28	56.9344678
CBX4	-1.15727513	6.42953464	1.06E-29	1.18E-28	56.9176642
GATAD1	-1.207146197	5.572430123	1.06E-29	1.19E-28	56.9127835
USP48	-1.103242807	5.535924452	1.08E-29	1.21E-28	56.8962739

TMOD3	-1.399797503	4.413208391	1.09E-29	1.22E-28	56.8834391
RP1-102H19.8	-1.059562033	2.884141624	1.10E-29	1.23E-28	56.877068
RRN3	-2.337432207	4.16244241	1.12E-29	1.25E-28	56.8548801
CEP68	-1.272811326	5.749718166	1.16E-29	1.29E-28	56.8246515
PGM2	-1.955742795	5.151985706	1.21E-29	1.35E-28	56.7785324
LOC100130357	-1.101259352	4.215780674	1.24E-29	1.38E-28	56.759321
EXOC6	-1.281681466	5.00506083	1.28E-29	1.42E-28	56.7244015
RYK	-1.045949828	4.974206069	1.31E-29	1.45E-28	56.7049406
SAR1B	-1.014997257	4.501493827	1.33E-29	1.48E-28	56.6856968
IKBKAP	-1.258458104	4.214738868	1.34E-29	1.48E-28	56.6791079
C8orf33	-1.101046031	4.592665515	1.34E-29	1.49E-28	56.6777974
HACL1	-1.384311797	5.59698798	1.42E-29	1.57E-28	56.6186299
ACYP1	-1.259810207	3.708010198	1.44E-29	1.59E-28	56.6064106
NUP54	-1.051361745	3.285929808	1.52E-29	1.67E-28	56.5552944
SERPINB2	-1.604062671	4.311151996	1.54E-29	1.69E-28	56.5395841
ATP6V1E2	-1.07035443	4.967418619	1.55E-29	1.71E-28	56.5303008
MGRN1	-1.575655767	7.484252947	1.57E-29	1.73E-28	56.5183976
TMEM168	-2.131574186	4.337753846	1.65E-29	1.81E-28	56.4684943
MAGEH1	-1.584778763	4.987653342	1.71E-29	1.87E-28	56.4359188
BRICD5	-1.21541876	5.797372595	1.72E-29	1.87E-28	56.4318058
TMED2	-2.14973642	5.358378492	1.78E-29	1.94E-28	56.3972225
HAVCR1	-1.083077493	3.001103725	1.79E-29	1.95E-28	56.3909864
ALG9	-1.036352403	4.390470288	1.79E-29	1.95E-28	56.3895954
SNRPB2	-1.058201783	4.863048073	1.82E-29	1.98E-28	56.3720328
PRKCH	-1.069325599	6.071546718	1.83E-29	1.99E-28	56.3690656
PHTF2	-1.104998773	3.715414538	1.83E-29	1.99E-28	56.3662742
MOB1B	-1.975019776	3.107583509	1.85E-29	2.01E-28	56.3580787
ARHGAP12	-1.947974034	3.381172083	1.90E-29	2.06E-28	56.3295272
ENY2	-1.143668836	4.426352305	1.94E-29	2.11E-28	56.3063941
LTV1	-1.124048887	4.440085735	1.97E-29	2.13E-28	56.2945348
SOX8	-1.025948997	3.609480947	2.08E-29	2.24E-28	56.2389618
CD38	-1.309285004	5.240851441	2.14E-29	2.31E-28	56.2098747
RP11-196G18.24	-2.084204351	3.93399048	2.19E-29	2.36E-28	56.1881087
AC006026.13	-1.092348655	3.754814781	2.21E-29	2.38E-28	56.1772449
INPP4A	-1.321674699	5.805855284	2.26E-29	2.43E-28	56.153558
ZDHHC13	-1.088465241	4.707923528	2.29E-29	2.45E-28	56.144402
PTPN22	-1.315447009	3.60237328	2.39E-29	2.56E-28	56.1000043
TSPAN11	-1.014055032	5.076298437	2.39E-29	2.56E-28	56.0979353
TTC9C	-1.409616962	5.571240368	2.43E-29	2.59E-28	56.0849018
MEIS1	-1.092076879	3.67667829	2.47E-29	2.63E-28	56.0686099
HIATL2	-1.033115225	6.638389984	2.49E-29	2.65E-28	56.0596164
TRIM35	-1.111033132	4.74158237	2.57E-29	2.74E-28	56.0266119
PIGA	-1.254016837	2.781761136	2.59E-29	2.76E-28	56.0177825
METTL21A	-1.425585023	3.861409836	2.64E-29	2.81E-28	55.9984906
ZNF37BP	-1.284987923	4.190222242	2.65E-29	2.81E-28	55.9961201
TAF1A	-1.13103523	2.814013519	2.65E-29	2.81E-28	55.9955801
ZNF45	-1.376116956	3.756327179	2.66E-29	2.82E-28	55.991723
N4BP2	-1.939920496	3.978179721	2.76E-29	2.93E-28	55.9554158
F8	-1.431021721	4.622317858	2.78E-29	2.94E-28	55.949068
MED17	-1.381469723	5.002874824	2.89E-29	3.06E-28	55.9079689
RNF19B	-1.775165973	6.82954753	2.91E-29	3.08E-28	55.9017156
PKD2	-1.685085643	3.12444266	2.94E-29	3.10E-28	55.8928999
PLOD3	-1.014692975	6.014374017	3.02E-29	3.18E-28	55.8658905
GFM2	-1.024317636	3.503524869	3.02E-29	3.19E-28	55.8649261
TTTY15	-1.141274196	3.862066452	3.05E-29	3.21E-28	55.8560176
CENPC	-1.782963707	3.945514376	3.09E-29	3.25E-28	55.842443
PCID2	-1.35182866	5.842760046	3.40E-29	3.56E-28	55.7472601
SLC25A43	-1.937323024	3.350185476	3.60E-29	3.77E-28	55.6893918

BCS1L	-1.087038522	5.939442416	3.76E-29	3.92E-28	55.6457894
ERN1	-1.283142263	5.441338789	3.80E-29	3.96E-28	55.6337567
ZC3H8	-1.01655954	4.287838051	3.83E-29	3.99E-28	55.6261912
GSR	-1.001644234	6.05386268	4.03E-29	4.19E-28	55.5755771
PIKFYVE	-1.335200071	4.838434093	4.07E-29	4.23E-28	55.5659577
TGIF1	-1.054810221	5.110681826	4.12E-29	4.27E-28	55.5544306
EFHD1	-1.086743576	4.309404877	4.18E-29	4.33E-28	55.5404084
NSMCE4A	-1.036811683	4.635886704	4.19E-29	4.34E-28	55.5367256
RP2	-1.881117454	3.740736463	4.45E-29	4.60E-28	55.4772634
RP11-548H18.2	-1.431251253	4.448428838	4.49E-29	4.64E-28	55.4676217
RADIL	-1.065162001	5.337732906	4.56E-29	4.70E-28	55.4527829
ZMAT3	-1.171675778	5.498007212	4.61E-29	4.74E-28	55.4424429
NARS2	-1.485047641	5.047604331	4.62E-29	4.75E-28	55.4401715
LOC100133131	-1.333716544	3.547743607	4.71E-29	4.84E-28	55.4198092
MLLT3	-1.388105297	4.518982529	5.02E-29	5.14E-28	55.3558167
CREBZF	-1.03754772	3.700769578	5.23E-29	5.34E-28	55.3158795
RNF34	-1.20816891	5.778586973	5.26E-29	5.36E-28	55.3100483
CYCS	-1.528436074	4.744189575	5.41E-29	5.52E-28	55.2813029
CHN1	-1.09094966	5.069038872	5.46E-29	5.56E-28	55.2716997
ASF1B	-1.305767013	5.903740261	5.50E-29	5.60E-28	55.2650898
RNF166	-2.187503801	6.7609142	5.81E-29	5.91E-28	55.2101377
GGH	-1.118679564	3.325461624	5.95E-29	6.05E-28	55.1853092
CD8B	-1.105757523	3.82178934	6.33E-29	6.43E-28	55.1243302
SMIM14	-1.245272492	5.230092393	6.55E-29	6.65E-28	55.0896428
NOG	-2.269426366	4.116807741	7.01E-29	7.10E-28	55.0210881
CDC37	-1.082469585	7.760815617	7.09E-29	7.17E-28	55.0094968
LOC100129461	-1.119994742	4.066556254	7.14E-29	7.22E-28	55.0025063
HNRNPD	-1.104088778	5.689353568	7.24E-29	7.31E-28	54.9888298
ABCD3	-1.755854312	3.968861204	7.32E-29	7.39E-28	54.9785981
VEZT	-1.029002495	3.115390241	7.34E-29	7.40E-28	54.9756722
RP11-288L9.1	1.279104874	5.569957118	7.42E-29	7.49E-28	54.9641693
ZNF224	-1.128505664	4.383630864	7.63E-29	7.69E-28	54.9361694
CCDC90B	-1.00422352	4.900785039	7.63E-29	7.69E-28	54.9359447
NR3C2	-1.515067402	3.97617347	7.88E-29	7.92E-28	54.904417
HYOU1	-1.20888645	7.529358885	8.38E-29	8.41E-28	54.8428365
CHST7	-1.569735436	6.000900057	8.41E-29	8.43E-28	54.8394167
FAM212B-AS1	-1.17736088	5.288290531	8.45E-29	8.47E-28	54.8340202
PPP1R21	-1.86029921	5.796326606	8.56E-29	8.57E-28	54.8213699
FAM171A1	-1.34302141	5.173263615	8.91E-29	8.92E-28	54.7812237
HS2ST1	-1.234892133	3.212961192	8.94E-29	8.95E-28	54.7774122
BZRAP1-AS1	-1.738643978	6.560622211	9.17E-29	9.17E-28	54.7519922
FBXO8	-1.646120506	4.016242793	9.34E-29	9.33E-28	54.7340742
ERLEC1	-1.220938623	4.97331777	9.88E-29	9.84E-28	54.6774717
CCDC186	-1.682295195	3.740780075	1.04E-28	1.03E-27	54.6298852
LETMD1	-1.268872285	6.825814659	1.08E-28	1.07E-27	54.5872072
SLC30A9	-1.217881844	3.179300318	1.15E-28	1.14E-27	54.5258315
SLC39A6	-1.655413404	4.756144132	1.17E-28	1.16E-27	54.5052982
FLI1	-2.10670237	5.561597559	1.19E-28	1.17E-27	54.4940812
ACRC	-1.383319252	3.856844216	1.20E-28	1.18E-27	54.4849575
LINC00282	-1.54301184	4.826689694	1.26E-28	1.24E-27	54.4347355
FRYL	-1.012188288	4.835333473	1.31E-28	1.29E-27	54.3988374
UBA2	-1.547162382	5.644715368	1.32E-28	1.30E-27	54.3874957
MTRR	-1.484243266	4.81940625	1.32E-28	1.30E-27	54.384065
ARIH1	-1.102410163	5.47166434	1.34E-28	1.32E-27	54.3702303
TATDN3	-1.042190921	4.957541618	1.35E-28	1.32E-27	54.3661291
C10orf32	-2.539772941	4.414133029	1.35E-28	1.33E-27	54.3653135
TNFSF4	-1.685497724	4.581950141	1.35E-28	1.33E-27	54.3627299
YPEL1	-1.058853675	5.260908719	1.38E-28	1.35E-27	54.3451035

ZNF137P	-1.778504793	4.643577661	1.40E-28	1.37E-27	54.32906
SEL1L3	-1.162256117	4.816642031	1.43E-28	1.40E-27	54.3080502
DAGLB	-1.007990683	5.338421795	1.44E-28	1.41E-27	54.3016281
C12orf43	-1.279492994	5.030113676	1.46E-28	1.43E-27	54.2858644
SCAMP1	-1.005560015	4.276082074	1.48E-28	1.45E-27	54.2719023
ELMO2	-1.010665279	6.083572012	1.49E-28	1.46E-27	54.2632351
DIEXF	-1.261967497	4.668640534	1.52E-28	1.48E-27	54.2470631
ITPR1	-1.227604566	5.403413474	1.53E-28	1.49E-27	54.2408991
CCNY	-1.243640312	5.890933274	1.54E-28	1.50E-27	54.2341266
TMEM125	-1.264725031	4.664490481	1.56E-28	1.52E-27	54.2221231
CEP78	-1.394658602	4.996523159	1.57E-28	1.53E-27	54.2108865
XPOT	-2.139752243	4.672000511	1.59E-28	1.55E-27	54.1981384
RRAS2	-1.36104723	3.758987479	1.68E-28	1.64E-27	54.1432321
MSLN	1.145923098	3.587418272	1.71E-28	1.66E-27	54.1256961
RP1-30M3.5	-2.049970557	4.791389293	1.78E-28	1.72E-27	54.0898344
BET1	-1.971719547	4.642529753	1.81E-28	1.76E-27	54.0687473
SNHG17	-1.395967101	5.934621306	1.85E-28	1.79E-27	54.0471929
RBM43	-1.298756488	4.075805862	1.90E-28	1.83E-27	54.0239475
CTA-246H3.12	-1.015163189	4.068548001	1.94E-28	1.87E-27	54.0034477
NELFCD	-1.066061346	6.805759418	1.94E-28	1.87E-27	54.0015599
KIDINS220	-1.148904301	4.893426855	2.00E-28	1.93E-27	53.969477
IST1	-1.168242921	5.90523401	2.03E-28	1.96E-27	53.9574945
TTC31	-1.449433309	6.641369321	2.05E-28	1.98E-27	53.9439662
KHDRBS1	-1.25874211	7.090652158	2.20E-28	2.12E-27	53.8771803
YTHDC2	-1.316053541	4.228832564	2.44E-28	2.34E-27	53.7717097
ARV1	-1.204166464	4.967764201	2.47E-28	2.37E-27	53.7598857
RHBDD2	-1.041062969	6.563449227	2.48E-28	2.38E-27	53.7558032
SEC24A	-1.18129712	4.043996289	2.58E-28	2.48E-27	53.7142054
EPB41L3	-1.768025891	4.252843164	2.59E-28	2.48E-27	53.7137031
MKLN1	-1.185190777	4.802751631	2.66E-28	2.54E-27	53.6855109
ZNF227	-1.394293867	3.771849532	2.71E-28	2.59E-27	53.6657242
CEP95	-1.131688934	4.163674568	2.72E-28	2.60E-27	53.6617089
MTFR1L	-1.124738414	6.523092956	2.75E-28	2.63E-27	53.6521827
ZNF367	-1.098759684	2.694694918	2.78E-28	2.65E-27	53.6418746
CDK5R1	-1.26212842	4.049424315	2.95E-28	2.81E-27	53.583096
HIBCH	-1.514631134	4.257696076	3.04E-28	2.89E-27	53.5531421
BBS2	-1.648176848	4.968819274	3.09E-28	2.94E-27	53.534682
SF3A1	-1.026645529	7.231706865	3.19E-28	3.03E-27	53.5042149
SPAST	-2.397782564	4.625253357	3.22E-28	3.06E-27	53.4931021
AUH	-1.377490351	4.409988351	3.26E-28	3.09E-27	53.4825045
PDP2	-1.172906844	4.272467313	3.27E-28	3.10E-27	53.4775877
RP4-635E18.8	-1.606540178	5.321607471	3.28E-28	3.11E-27	53.4761144
APC	-1.281387448	4.829657093	3.34E-28	3.16E-27	53.4571125
MRPS14	-1.114196697	5.167390117	3.38E-28	3.20E-27	53.4458531
SSBP1	-1.037250553	5.906892471	3.45E-28	3.26E-27	53.4245317
LARS	-1.185226652	6.703740821	3.50E-28	3.30E-27	53.411563
CTNNB1	-1.08546823	5.337965985	3.58E-28	3.37E-27	53.3881947
RBM7	-1.818877348	5.174304689	3.85E-28	3.62E-27	53.3142953
ERO1L	-1.590133803	5.916640733	3.96E-28	3.72E-27	53.2858089
LOC101927204	-1.0378466	4.309342604	4.07E-28	3.81E-27	53.2591686
MIA3	-1.000916947	5.171630047	4.12E-28	3.85E-27	53.2459088
BRPF1	-1.171417992	6.115641829	4.20E-28	3.92E-27	53.2289702
CNTRL	-1.55740768	5.025495062	4.27E-28	3.98E-27	53.210804
SMARCC2	-1.513204904	6.306063583	4.41E-28	4.10E-27	53.1795694
NIPA2	-1.032682438	6.698852359	4.44E-28	4.13E-27	53.1730554
RUFY3	-1.002378868	4.643355189	4.49E-28	4.18E-27	53.1599462
PDGFD	-1.174455672	4.694282805	4.82E-28	4.48E-27	53.089003
TRMT10B	-1.048302013	5.079040988	4.83E-28	4.48E-27	53.0878228

METTL2B	-1.340120091	4.171070736	4.84E-28	4.49E-27	53.0857956
PRPF38A	-1.136633262	5.159388139	4.87E-28	4.52E-27	53.0787056
CEP135	-1.739152995	3.841271064	4.89E-28	4.54E-27	53.0748499
NUP62	-1.336338999	5.197279623	5.66E-28	5.23E-27	52.9288967
LINC00959	-1.245374569	3.768889068	5.71E-28	5.28E-27	52.9195261
ZNF818P	-1.352419861	3.959211441	5.80E-28	5.36E-27	52.9044603
SEC63	-1.443277568	4.455881076	5.85E-28	5.40E-27	52.8964355
MLF2	-1.094555821	8.437638172	6.07E-28	5.60E-27	52.8594976
SLAIN2	-1.144659198	4.869642964	6.18E-28	5.70E-27	52.8410102
TAS2R39	-1.034012924	3.928132463	6.97E-28	6.41E-27	52.7209034
ZNF611	-1.109247695	4.297304502	7.18E-28	6.60E-27	52.6899722
CASP8AP2	-1.62759286	4.067975467	7.35E-28	6.74E-27	52.6674325
CAPZB	-1.014294712	7.962056998	7.69E-28	7.04E-27	52.6212335
ALDH9A1	-1.21670474	8.352350478	7.83E-28	7.15E-27	52.6040262
GLTSCR2	1.554264412	5.871226742	7.85E-28	7.17E-27	52.6015254
CD300LF	-1.375321415	8.404518512	7.88E-28	7.19E-27	52.5979628
S100PBP	-1.67438488	5.440317075	8.30E-28	7.56E-27	52.5454889
SRPK2	-1.668068173	5.466568072	8.35E-28	7.60E-27	52.539383
CBX7	-1.192382798	8.353401236	8.62E-28	7.84E-27	52.5072517
GRPEL2	-1.087327134	3.390449749	8.64E-28	7.86E-27	52.5047194
LRRC58	-1.200342963	3.503989343	8.70E-28	7.91E-27	52.4977217
AMMECR1	-1.196447464	4.320820196	8.97E-28	8.14E-27	52.4677967
ATP8B2	-1.203956212	6.084822218	9.04E-28	8.21E-27	52.4592564
PTP4A1	-1.595597265	4.631310484	9.19E-28	8.32E-27	52.4435961
UBE2V2	-1.017578894	3.378152021	9.47E-28	8.56E-27	52.4134225
SUCLG2	-2.327190192	5.193181444	9.72E-28	8.78E-27	52.3870653
PFAS	-1.57522597	4.806755062	9.83E-28	8.88E-27	52.3756023
ZNF33A	-1.033700945	5.034693781	9.91E-28	8.94E-27	52.3677861
SLC30A1	-1.299386276	4.201323297	1.05E-27	9.42E-27	52.313131
MTERF3	-1.875322718	3.745046601	1.11E-27	9.93E-27	52.2584947
RSPRY1	-1.244854059	5.858809788	1.11E-27	9.93E-27	52.2580316
NUDT16L1	-1.252118485	5.240199818	1.11E-27	9.94E-27	52.2562913
FBXO28	-1.258773517	4.664567502	1.12E-27	1.00E-26	52.2480564
NPHP3	-1.732060275	4.192184973	1.19E-27	1.06E-26	52.1881541
SARS	-1.064322808	5.443901808	1.19E-27	1.06E-26	52.1879825
BYSL	-1.049562923	5.538695588	1.20E-27	1.07E-26	52.1775025
LINC00277	-1.001464593	4.839423749	1.28E-27	1.14E-26	52.1123239
RNF38	-1.59443947	5.702547526	1.31E-27	1.17E-26	52.0869553
CBR4	-1.253994944	5.628577	1.37E-27	1.22E-26	52.0400327
PLEKHM3	-1.34864741	5.314240395	1.38E-27	1.23E-26	52.0340376
DNAJB11	-1.206758706	6.376263279	1.42E-27	1.26E-26	52.010004
NUDT13	-1.073120488	3.522424243	1.42E-27	1.26E-26	52.006636
YARS2	-1.447923758	5.148391649	1.43E-27	1.27E-26	52.0034885
NOA1	-1.32520576	6.840930715	1.44E-27	1.28E-26	51.9937819
GPATCH8	-1.083789756	6.897380992	1.46E-27	1.29E-26	51.9800927
SMARCAD1	-1.840215733	4.814876499	1.47E-27	1.30E-26	51.9739985
STAM	-2.560043345	4.772471859	1.47E-27	1.31E-26	51.9697721
ACAD8	-1.813003268	5.050145709	1.50E-27	1.32E-26	51.9546826
USP32P2	-2.692277916	4.923318501	1.50E-27	1.32E-26	51.9545503
ZNF184	-1.343757402	3.845591911	1.51E-27	1.34E-26	51.9436771
RBM12B	-1.920961748	4.153864578	1.51E-27	1.34E-26	51.9435353
RBM12	-1.737255327	5.018960693	1.52E-27	1.34E-26	51.9422955
ITFG1	-1.191046013	4.019909275	1.55E-27	1.37E-26	51.9199154
SRSF6	-1.375517926	6.129786053	1.58E-27	1.39E-26	51.9025355
ADCY10P1	-1.064736817	4.540682027	1.59E-27	1.40E-26	51.8963108
CAST	-1.146638314	6.11046155	1.75E-27	1.53E-26	51.797904
IFT80	-1.087330667	3.165853678	1.76E-27	1.54E-26	51.793749
PRIMPOL	-1.313526076	4.78188038	1.84E-27	1.60E-26	51.7504251

IKBKB	-1.305682038	5.93218508	1.91E-27	1.66E-26	51.7113638
NEDD1	-1.525810299	4.081174923	1.94E-27	1.70E-26	51.6922389
MFN1	-1.607010875	4.004846411	1.96E-27	1.71E-26	51.6862552
LPXN	-1.235549959	6.506184148	1.97E-27	1.72E-26	51.6796828
CD47	-1.573875501	6.463046504	2.02E-27	1.76E-26	51.656116
METAP2	-1.325705598	4.338087346	2.07E-27	1.80E-26	51.6289629
SRSF4	-1.300061059	5.425182405	2.10E-27	1.83E-26	51.6156951
RP11-532F12.5	-1.006144431	5.213837012	2.10E-27	1.83E-26	51.6147509
RDH13	-1.042460609	5.159124627	2.14E-27	1.86E-26	51.5950092
LOC101928054	-1.70491302	4.57924099	2.29E-27	1.99E-26	51.5271597
ZNF143	-1.736182103	5.960352727	2.34E-27	2.02E-26	51.5080267
NEU4	1.010023242	6.418825527	2.36E-27	2.04E-26	51.5000626
MORC2	-1.075695896	5.900655996	2.46E-27	2.12E-26	51.4588423
CDC73	-1.568489818	4.147165661	2.52E-27	2.18E-26	51.4321042
RFK	-1.701865286	4.254198417	2.69E-27	2.32E-26	51.3665568
TOR1AIP1	-1.413478054	5.505437381	2.69E-27	2.32E-26	51.3664829
MRPS10	-1.070657901	5.807903705	2.76E-27	2.37E-26	51.3427995
MDM4	-1.179640138	6.233238016	2.83E-27	2.43E-26	51.3169086
CUZD1	-1.065020944	3.400321916	2.86E-27	2.45E-26	51.3074631
HEATR3	-1.644275393	3.935028784	2.87E-27	2.46E-26	51.3037874
CELA3B	-1.01077675	5.601529866	2.87E-27	2.46E-26	51.3020646
C14orf169	-1.092166374	6.217474945	2.95E-27	2.53E-26	51.2762381
RP5-894A10.6	-1.003963393	4.482559848	2.96E-27	2.54E-26	51.2720186
MISP	1.391051351	7.301084192	3.01E-27	2.58E-26	51.2555743
PPP1R3E	-1.158089856	5.20724405	3.05E-27	2.61E-26	51.242956
STK38	-1.022721268	6.40204432	3.42E-27	2.92E-26	51.1266771
AC009120.6	-1.119260641	5.055984984	3.48E-27	2.97E-26	51.108981
DDX18	-1.333782049	6.118049298	3.50E-27	2.99E-26	51.1022441
SCML1	-1.610845186	3.848280054	3.55E-27	3.03E-26	51.0882852
LOC101927365	-1.453154122	4.176243676	3.59E-27	3.05E-26	51.0789144
ERAP1	-1.215389357	4.530055957	3.62E-27	3.08E-26	51.0693208
TRIM27	-1.169245282	7.14750029	3.69E-27	3.13E-26	51.0514208
CHD9	-1.208470446	4.176890108	3.74E-27	3.17E-26	51.0367168
APBA2	-1.201447865	6.810169194	3.78E-27	3.20E-26	51.0260283
LRCH3	-1.139439042	4.856562751	3.88E-27	3.28E-26	51.001075
SNX1	-1.080072586	5.850646658	4.22E-27	3.57E-26	50.9151236
LOC646626	-1.011887113	3.890765773	4.28E-27	3.61E-26	50.9016168
PPM1D	-1.162612828	4.782135894	4.29E-27	3.62E-26	50.9006637
ENOPH1	-2.197662947	4.965554575	4.45E-27	3.75E-26	50.8627089
PDE7A	-1.21942389	5.198926312	4.52E-27	3.80E-26	50.84791
ARF6	-1.455842748	7.175004609	4.61E-27	3.88E-26	50.8267088
CEACAM4	-1.118189847	6.978127563	4.66E-27	3.92E-26	50.8162318
SKAP1	-1.202684157	6.720684425	4.67E-27	3.93E-26	50.8140133
NDC1	-1.120460862	4.387764985	4.85E-27	4.07E-26	50.7771713
SRD5A1	-1.23191161	5.088255823	4.88E-27	4.09E-26	50.7707614
LEO1	-1.076935046	4.91394264	4.92E-27	4.13E-26	50.7622093
HTR1B	-1.272188708	3.846395178	4.96E-27	4.16E-26	50.7542777
SPATA5L1	-1.54450092	5.146447961	4.97E-27	4.16E-26	50.7531278
PUS7	-1.406367422	3.623268621	5.29E-27	4.42E-26	50.6900636
DFNA5	-1.249066599	3.767829963	5.32E-27	4.45E-26	50.6838423
SIDT1	-1.891701908	6.061544755	5.56E-27	4.63E-26	50.6406508
POLD1	-1.030011459	6.685720598	5.89E-27	4.91E-26	50.5814092
PAN2	-1.077471397	5.690986514	6.01E-27	5.00E-26	50.5621805
PRKDC	-1.275245335	5.066118034	6.03E-27	5.01E-26	50.5589543
MSL2	-1.971444266	5.782248439	6.08E-27	5.05E-26	50.5510692
RUNDC1	-1.184124193	5.370598131	6.21E-27	5.15E-26	50.529344
DUSP12	-1.411417253	5.788736831	6.45E-27	5.34E-26	50.4907776
SYNCRIP	-1.216825151	5.284320835	6.62E-27	5.47E-26	50.4655049

BUD13	-1.085591579	5.770577468	6.67E-27	5.52E-26	50.456907
MGEA5	-1.574345417	6.879261201	6.69E-27	5.53E-26	50.4541288
VAV3	-1.280618803	4.553362344	6.77E-27	5.59E-26	50.4423362
DENR	-1.318721432	4.954755495	6.90E-27	5.69E-26	50.4241128
IL12RB2	-1.254104796	4.059757759	6.90E-27	5.69E-26	50.4239084
POLR2G	-1.196925418	8.154658387	7.09E-27	5.84E-26	50.3962599
NEMF	-1.624529862	4.798876266	7.15E-27	5.88E-26	50.387729
TNRC6C-AS1	-1.254892586	6.323871547	7.33E-27	6.03E-26	50.3626488
ANKRD13C	-1.058884432	2.937576894	7.62E-27	6.25E-26	50.3247951
VPS54	-1.729415623	4.534596754	7.67E-27	6.29E-26	50.3175672
MTMR11	-1.035311629	5.831727565	7.82E-27	6.40E-26	50.2986619
RBM18	-1.462106006	4.656905586	8.28E-27	6.77E-26	50.2413764
SMARCE1	-1.021861618	4.878287782	8.33E-27	6.81E-26	50.2352503
HDAC1	-1.393490526	7.560205411	8.59E-27	7.03E-26	50.2036099
KIF2A	-2.110665564	4.484684703	8.74E-27	7.14E-26	50.18674
ADI1	-1.026320324	6.111046212	9.08E-27	7.40E-26	50.1489649
LPCAT1	-1.175310537	7.894890957	9.22E-27	7.51E-26	50.1337088
SPTLC1	-1.340523262	4.599455936	9.23E-27	7.52E-26	50.1320284
SLC25A12	-1.011782296	4.437840979	9.25E-27	7.53E-26	50.1300262
SRSF11	-1.650785289	5.017145406	9.63E-27	7.82E-26	50.0893026
UBR3	-1.385805856	3.620149815	9.78E-27	7.93E-26	50.0746569
OPN3	-1.592168634	4.732124125	9.92E-27	8.04E-26	50.0602607
FAM162A	-1.026756097	5.410750635	1.04E-26	8.44E-26	50.0114676
MON1B	-1.023027911	6.443422419	1.05E-26	8.48E-26	50.0063302
CIRBP	-1.093782756	7.236571813	1.05E-26	8.54E-26	49.9983437
RIN2	-1.328507531	4.708120861	1.07E-26	8.63E-26	49.9873178
TYW3	-1.275106955	4.385411145	1.08E-26	8.71E-26	49.9770182
RCOR1	-1.023070183	6.338870039	1.09E-26	8.81E-26	49.9650345
TMC6	-1.339646681	7.59715018	1.14E-26	9.21E-26	49.9205699
SMAD4	-1.129871935	4.397015996	1.18E-26	9.50E-26	49.8873917
TTC4	-1.014597088	5.620861509	1.19E-26	9.59E-26	49.8774474
ALYREF	-1.090181226	4.263855127	1.19E-26	9.60E-26	49.8764764
LOC100505501	-1.443728397	4.294290472	1.28E-26	1.03E-25	49.8071644
PTCD2	-1.084990903	5.155359425	1.28E-26	1.03E-25	49.804876
GP5	-1.029238961	3.212468425	1.38E-26	1.11E-25	49.7256817
ASH1L	-1.362371127	6.216979309	1.39E-26	1.12E-25	49.7217042
PAK2	-1.347281928	5.696515015	1.44E-26	1.16E-25	49.6853213
ST6GAL1	-1.038703053	4.94693671	1.45E-26	1.16E-25	49.6825137
CCDC146	-1.11473146	4.609755306	1.55E-26	1.24E-25	49.6099541
UGDH	-1.272126652	3.921471326	1.59E-26	1.27E-25	49.5882382
FTSJ1	-1.013168384	5.733605372	1.60E-26	1.27E-25	49.5834658
PSMD7	-1.316030685	7.601063571	1.60E-26	1.28E-25	49.5782322
GALNT3	-1.173520322	4.671682866	1.72E-26	1.37E-25	49.507213
MAP7D1	-1.339898271	7.360859647	1.80E-26	1.42E-25	49.4645656
PHYH	-1.144928683	3.923830646	1.83E-26	1.45E-25	49.4484202
CBFB	-1.336488979	6.350215137	1.84E-26	1.46E-25	49.4402577
ZSCAN16	-1.257651557	4.298579962	1.86E-26	1.47E-25	49.4305001
ZKSCAN4	-1.005122499	5.151165094	1.86E-26	1.47E-25	49.4282353
SDE2	-1.649353038	4.988003388	1.87E-26	1.48E-25	49.4243584
NXT2	-1.626331347	4.197153172	1.87E-26	1.48E-25	49.4235047
ZSCAN18	-1.225769033	5.954642146	1.91E-26	1.50E-25	49.4058994
NCK1	-1.38944008	4.133457561	1.91E-26	1.51E-25	49.4021448
DIS3	-1.307256783	4.409717655	1.94E-26	1.52E-25	49.3904416
ERMP1	-1.067861975	4.904419897	1.94E-26	1.52E-25	49.3891401
DBF4	-1.804029158	3.951731191	1.95E-26	1.53E-25	49.3847577
PDE4B	-1.880020048	5.126165718	1.98E-26	1.56E-25	49.3682026
COMMD2	-1.746199136	4.579424911	2.03E-26	1.60E-25	49.3419612
CRY1	-2.516160215	3.678987594	2.15E-26	1.69E-25	49.2854911

MAT2A	-1.060227613	6.208513675	2.16E-26	1.69E-25	49.2824999
MED1	-1.137282548	5.834967817	2.17E-26	1.70E-25	49.2737165
SART3	-1.012944709	5.604434006	2.25E-26	1.76E-25	49.2388105
HMG20A	-1.864114048	4.19026696	2.31E-26	1.81E-25	49.2129528
PIK3C2B	-1.157113755	6.87959419	2.34E-26	1.83E-25	49.201189
NAT2	-1.019207174	3.732845291	2.37E-26	1.85E-25	49.1891328
DHX36	-1.090098958	6.281444671	2.37E-26	1.85E-25	49.1889731
PRKAB2	-1.087005554	4.68061634	2.50E-26	1.95E-25	49.1338814
KIAA1147	-1.077250695	6.129473177	2.54E-26	1.98E-25	49.1196481
MGAT2	-1.447265228	5.363591659	2.59E-26	2.02E-25	49.0985576
TSHZ1	-1.875008424	5.641841842	2.67E-26	2.07E-25	49.0691412
TMF1	-1.372713839	5.144880411	2.67E-26	2.08E-25	49.066852
ENTPD4	-1.055661117	6.125322163	2.88E-26	2.23E-25	48.9928548
ACVR1C	-1.1716765	3.696838298	2.90E-26	2.25E-25	48.9853265
RP11-452L6.1	-1.003878141	4.644000347	3.00E-26	2.32E-25	48.9514098
ACADSB	-1.00105958	3.169059131	3.09E-26	2.38E-25	48.9228405
RP11-747H7.3	-1.452837902	5.572191861	3.10E-26	2.40E-25	48.9173761
KLF12	-1.043272303	4.996131653	3.12E-26	2.41E-25	48.9127992
UBE2Z	-1.11294787	7.401931004	3.14E-26	2.42E-25	48.9059622
CRTAM	-2.156836711	4.254618475	3.21E-26	2.48E-25	48.8832855
PTCD3	-1.566170745	5.547231415	3.27E-26	2.52E-25	48.8638581
COPA	-1.102919752	5.448381569	3.33E-26	2.56E-25	48.8473374
LGALS8	-1.346384172	5.44260174	3.40E-26	2.61E-25	48.8246766
ZBTB14	-1.033141145	4.408346033	3.41E-26	2.62E-25	48.8239278
SCML4	-1.081203501	5.743513049	3.52E-26	2.70E-25	48.7920921
BOD1	-1.409351018	5.008717323	3.61E-26	2.77E-25	48.7661937
LOC374443	-1.44878169	4.303877951	3.69E-26	2.82E-25	48.7444841
CSK	-1.162050497	9.149324777	3.76E-26	2.88E-25	48.7242403
ANK3	-1.029479006	3.93980911	4.03E-26	3.08E-25	48.6551655
KBTBD6	-1.161137291	3.665333095	4.08E-26	3.11E-25	48.6439305
MFF	-1.40127873	5.370261601	4.14E-26	3.16E-25	48.6282783
COX18	-1.150932748	4.828366738	4.24E-26	3.24E-25	48.6038099
PMPCA	-1.182561127	6.443248845	4.32E-26	3.29E-25	48.5860886
MED29	-1.738599285	5.519372343	4.38E-26	3.34E-25	48.5719938
RP11-226L15.5	-1.856040907	3.335269621	4.47E-26	3.40E-25	48.5526891
LIN7C	-1.616817688	4.601690638	4.54E-26	3.45E-25	48.5364456
ZNF430	-1.514627852	4.834220225	4.59E-26	3.48E-25	48.5262427
C20orf144	-1.051646971	3.748948646	4.72E-26	3.59E-25	48.4972659
C8orf76	-1.443778538	3.736162123	4.85E-26	3.68E-25	48.4705111
PADI2	-1.243185352	5.764259936	5.43E-26	4.12E-25	48.3563797
STK39	-2.181385816	4.631175309	5.49E-26	4.16E-25	48.3456645
BCL10	-1.21675731	5.07795736	6.03E-26	4.56E-25	48.2515947
THAP9-AS1	-2.45804613	3.588353194	6.16E-26	4.65E-25	48.2309257
HSDL1	-1.249395364	4.403505624	6.18E-26	4.67E-25	48.2272215
AMZ2	-1.298086473	7.544070037	6.37E-26	4.80E-25	48.1964115
RHOU	-1.109306945	4.068375475	6.46E-26	4.86E-25	48.1837854
TMOD2	-1.096466892	3.371034885	6.59E-26	4.95E-25	48.1633394
COG8	-1.06556223	5.937361446	6.69E-26	5.03E-25	48.1472771
CCDC126	-1.484374455	4.266733335	6.88E-26	5.16E-25	48.1193501
C15orf37	-1.309207712	5.212234368	7.19E-26	5.38E-25	48.0759451
NET1	-1.228071039	3.732798198	7.25E-26	5.43E-25	48.0668132
LOC100129447	-1.072702798	3.953282467	7.35E-26	5.50E-25	48.0534589
C2orf68	-1.108198424	6.286863266	7.44E-26	5.56E-25	48.0409294
RP3-522P13.2	1.485174768	5.316028627	7.47E-26	5.58E-25	48.0380469
DUSP15	1.261681332	4.696971163	7.50E-26	5.60E-25	48.0331304
ERP27	-1.346686575	6.170528793	7.87E-26	5.86E-25	47.9858634
FAM98A	-1.166870039	4.970824736	7.87E-26	5.86E-25	47.9852148
SPCS2	-1.134223774	6.683039163	8.06E-26	5.99E-25	47.9619302

NOXO1	1.049725389	5.452942217	8.10E-26	6.02E-25	47.9570357
SIAH1	-1.476466936	4.15090968	8.12E-26	6.04E-25	47.9539351
PPP1CA	-1.15594382	9.279888384	8.49E-26	6.30E-25	47.90888
PEBP1	-1.240130885	7.1350873	8.51E-26	6.31E-25	47.9067875
PTPRN	-1.19284839	4.141101446	8.82E-26	6.53E-25	47.8708172
ALDH6A1	-1.021073838	4.787446233	8.85E-26	6.55E-25	47.867552
ZNF318	-1.066326727	4.893986485	8.92E-26	6.59E-25	47.8601628
MAN2B2	-1.39407867	5.975100786	9.11E-26	6.73E-25	47.8384945
PPIL4	-1.592348471	2.880145844	9.17E-26	6.77E-25	47.8317075
ELMO1	-1.406002382	6.483726231	9.24E-26	6.82E-25	47.8245728
ZBTB6	-1.122623316	2.411848483	9.64E-26	7.10E-25	47.7823473
SAMD8	-1.575713217	3.82700429	9.90E-26	7.28E-25	47.7551087
CD3E	-1.560475081	8.619728078	9.95E-26	7.31E-25	47.750094
2-Sep	-1.19100058	6.708141995	1.01E-25	7.40E-25	47.737734
BTN2A1	-1.1415619	5.77875935	1.02E-25	7.47E-25	47.7286322
NUP153	-1.696609562	5.315563288	1.03E-25	7.57E-25	47.713674
GEMIN5	-1.181168168	5.420845721	1.03E-25	7.58E-25	47.7121938
ZBTB48	-1.204615184	6.371119891	1.09E-25	7.96E-25	47.6605708
HENMT1	-1.339142997	6.074493696	1.09E-25	7.97E-25	47.6587157
RFC5	-1.327486865	4.523061641	1.09E-25	7.98E-25	47.6563407
SLC35A3	-1.959146943	4.736118581	1.09E-25	7.99E-25	47.654904
HSPA4	-1.092257665	5.23259005	1.10E-25	8.01E-25	47.6519039
CNN2	-1.118672999	9.356730458	1.11E-25	8.11E-25	47.6382923
MLC1	-1.271764094	5.37772506	1.14E-25	8.30E-25	47.6149259
ATP6V0E2	-1.212620291	6.813545833	1.14E-25	8.33E-25	47.6101134
FAM172A	-1.240008341	4.999648972	1.15E-25	8.39E-25	47.6030079
GPR162	1.232126267	5.604410945	1.16E-25	8.41E-25	47.6006323
TMEM55A	-3.028904993	4.020446216	1.16E-25	8.44E-25	47.5970362
HSCB	-1.435407055	4.957086735	1.17E-25	8.50E-25	47.5892656
DPF2	-1.408491903	7.638737866	1.20E-25	8.70E-25	47.5646367
C2orf40	-1.260871105	4.015323528	1.20E-25	8.70E-25	47.564487
ORMDL1	-1.607269818	5.385289461	1.22E-25	8.86E-25	47.5455157
LUC7L2	-1.337627158	6.144194851	1.23E-25	8.89E-25	47.5415221
ACN9	-1.270440274	2.780935878	1.24E-25	8.98E-25	47.5300842
BD495725	-1.605214311	6.059197871	1.26E-25	9.12E-25	47.5134888
ASCC1	-1.138098335	5.064200025	1.26E-25	9.15E-25	47.5099282
YTHDF1	-1.023915664	7.390048999	1.29E-25	9.32E-25	47.4899231
ATG12	-1.101546458	5.814846791	1.30E-25	9.41E-25	47.4803355
PPP1R35	-1.214488873	6.412430547	1.33E-25	9.62E-25	47.4574996
RP1-193H18.2	-1.305601933	4.613828618	1.40E-25	1.01E-24	47.408056
MDH2	-1.101885567	6.832390375	1.42E-25	1.02E-24	47.3971348
CDV3	-1.251259371	6.271860359	1.42E-25	1.02E-24	47.3953671
ZSWIM7	-1.005232501	4.87159383	1.42E-25	1.02E-24	47.391244
MICALL1	-1.259706345	5.918582749	1.44E-25	1.03E-24	47.3808218
THEMIS	-2.443242962	3.780871544	1.48E-25	1.06E-24	47.3542486
RIOK1	-1.825276354	4.628106214	1.48E-25	1.06E-24	47.3532855
MDM1	-1.575746255	3.234387956	1.50E-25	1.07E-24	47.3423816
MFSD6	-1.40298868	4.241931779	1.50E-25	1.07E-24	47.3421956
PRNP	-2.288409036	5.197010936	1.50E-25	1.08E-24	47.3374487
ATXN1L	-1.407032284	6.198055235	1.51E-25	1.08E-24	47.3353559
KIAA0040	-1.053483358	6.71603705	1.53E-25	1.09E-24	47.3211655
ALG13	-1.56886449	4.292540989	1.54E-25	1.10E-24	47.3116323
SNRNP48	-1.162254269	4.03058806	1.63E-25	1.16E-24	47.2565379
ZNF654	-1.476389577	3.586997762	1.63E-25	1.16E-24	47.2547525
NCEH1	-1.193629929	2.775858797	1.64E-25	1.17E-24	47.2525563
SLC9A3R1	-1.004403743	8.141105617	1.69E-25	1.21E-24	47.21822
ABHD16B	1.083026183	4.660426047	1.70E-25	1.21E-24	47.2150939
TBCC	-1.080074601	7.530739752	1.70E-25	1.21E-24	47.2132037

GATA3	-1.260710643	5.375095945	1.78E-25	1.26E-24	47.1687489
REV3L	-1.866356354	5.311050292	1.84E-25	1.31E-24	47.1352944
MVB12A	-1.369697287	5.041117806	1.93E-25	1.36E-24	47.0893154
PPIL1	-1.482811171	4.123758114	1.95E-25	1.38E-24	47.0753825
CD7	-1.628088842	6.171589278	1.96E-25	1.38E-24	47.0737819
PTPN12	-1.559565275	4.206217179	1.99E-25	1.41E-24	47.0563058
MIER1	-1.388305028	4.58300692	2.01E-25	1.42E-24	47.0458892
NXPE3	-1.270764055	4.702677577	2.01E-25	1.42E-24	47.0445546
DIP2B	-1.337026718	5.397427544	2.02E-25	1.43E-24	47.0423742
CRYGC	1.187568141	4.762514065	2.21E-25	1.56E-24	46.9499094
ABHD10	-1.18063699	5.516820271	2.32E-25	1.63E-24	46.9034996
MYBL2	1.053565804	6.825817471	2.42E-25	1.69E-24	46.8619231
TCF7	-1.47789566	7.710960083	2.63E-25	1.84E-24	46.7752693
RNF144B	-1.118835988	5.971442692	2.68E-25	1.87E-24	46.7596397
DNAJC10	-1.342814898	3.772523594	2.68E-25	1.87E-24	46.7571911
SEC23B	-1.50045345	5.910765977	2.69E-25	1.88E-24	46.7544042
FAM117B	-1.177791236	4.917457535	2.70E-25	1.88E-24	46.7518135
IBTK	-1.620507791	3.533021076	2.73E-25	1.90E-24	46.7402824
GSPT2	-1.871214309	4.540595111	2.80E-25	1.95E-24	46.71518
DERL1	-1.152123821	6.177215392	2.83E-25	1.97E-24	46.7049327
LHFPL3-AS2	-1.150899186	5.117916958	2.85E-25	1.98E-24	46.6962896
EIF3M	-1.005835541	5.152549453	2.91E-25	2.02E-24	46.6769426
ZNF805	-1.116520256	4.186525039	3.01E-25	2.09E-24	46.6405641
C1orf174	-1.395411073	5.00107738	3.02E-25	2.10E-24	46.6381553
SNN	-1.097732939	7.430394785	3.04E-25	2.11E-24	46.6331264
ELF2	-1.264741536	5.85007094	3.10E-25	2.15E-24	46.6118257
PKD2L1	1.077705655	6.269787429	3.18E-25	2.20E-24	46.587745
RLF	-1.46290305	4.649073845	3.39E-25	2.34E-24	46.5238486
FERMT3	-1.083189452	8.264972948	3.48E-25	2.40E-24	46.4965583
FAM3C	-1.208781701	2.988268316	3.57E-25	2.46E-24	46.4705968
ATMIN	-1.068980451	5.475202287	3.60E-25	2.48E-24	46.4624066
NONO	-1.357035604	8.007454972	3.62E-25	2.49E-24	46.456553
GTF2E1	-1.548573751	4.781104186	3.74E-25	2.57E-24	46.4253338
ZFYVE26	-1.046650274	5.823544166	3.79E-25	2.61E-24	46.4103555
KBTBD8	-1.323859331	3.211576111	3.79E-25	2.61E-24	46.4101365
C11orf54	-1.351397703	5.534315771	3.80E-25	2.61E-24	46.4073127
RAP1GAP2	-1.400572753	7.815251926	3.88E-25	2.66E-24	46.3867356
ALPL	-2.035847726	5.526250016	4.01E-25	2.75E-24	46.3532066
TRMT1L	-1.61603362	3.688059095	4.15E-25	2.84E-24	46.3199523
DPAGT1	-1.309255633	5.577167571	4.22E-25	2.89E-24	46.3024178
FAM160A2	-1.050159507	6.351357565	4.36E-25	2.98E-24	46.2700798
STRBP	-1.202463899	4.624126727	4.41E-25	3.01E-24	46.2594593
SLC35A4	-1.137442817	7.3390044	4.42E-25	3.01E-24	46.2574031
CELA1	-1.047788192	5.175695173	4.42E-25	3.01E-24	46.2568624
NUCB2	-1.282507467	4.541360009	4.42E-25	3.01E-24	46.2567325
TGIF2	-1.171191077	5.442211792	4.58E-25	3.12E-24	46.2216284
LOC150051	-1.055424305	4.14686874	4.80E-25	3.26E-24	46.1743846
CTC-428G20.3	-1.778146644	4.398231936	4.87E-25	3.31E-24	46.1599218
ZNF689	-1.029250749	5.955544908	4.95E-25	3.36E-24	46.1428481
SMAD7	-1.822523139	4.810129197	4.98E-25	3.38E-24	46.1367623
LOC100506302	-1.079140245	5.327998682	5.00E-25	3.39E-24	46.1336236
RAB22A	-1.518388686	5.282757702	5.09E-25	3.45E-24	46.1164662
DNAJB9	-1.485450092	3.847675534	5.12E-25	3.47E-24	46.1093717
RNF169	-1.127002518	6.257810057	5.37E-25	3.63E-24	46.0618083
SNX30	-1.935570389	4.653389427	5.39E-25	3.65E-24	46.0573213
JAKMIP1	-1.082554022	4.276275309	5.51E-25	3.72E-24	46.0362263
IMP4	-1.112373974	6.834292156	5.54E-25	3.74E-24	46.030746
NKRF	-1.06353335	4.989480631	5.76E-25	3.88E-24	45.9922654

NOV	-1.299499065	5.065186104	6.24E-25	4.20E-24	45.9119155
HIPK3	-1.228206943	4.800354226	6.30E-25	4.24E-24	45.9023153
KLHL5	-1.454740826	4.375139622	6.33E-25	4.25E-24	45.8974077
SLC38A9	-1.321638033	4.61987627	6.34E-25	4.26E-24	45.8959894
PAIP1	-1.371366343	4.244044598	6.40E-25	4.29E-24	45.8868623
KLHL15	-2.137517539	4.328162847	6.41E-25	4.30E-24	45.8847377
EPHX2	-1.178406737	4.659202578	6.56E-25	4.39E-24	45.8620109
KCNK6	-1.265697272	6.809025923	6.61E-25	4.42E-24	45.8537748
C1GALT1C1	-1.331800634	4.169432237	6.91E-25	4.62E-24	45.8096671
PPP1R8	-1.712952323	6.945274391	7.03E-25	4.69E-24	45.7926653
AOC2	-1.176308595	4.885298253	7.30E-25	4.87E-24	45.7539498
AIMP1	-1.562831408	4.391885127	7.34E-25	4.89E-24	45.7489799
STK10	-1.256501041	8.046095973	7.59E-25	5.05E-24	45.7158577
HYLS1	-1.046793912	5.55944172	7.96E-25	5.29E-24	45.6679866
PLRG1	-1.13870703	4.94491554	8.15E-25	5.41E-24	45.6434591
NMRK1	-1.652789477	4.976685882	8.32E-25	5.51E-24	45.6238311
NAPG	-1.282446483	3.858834707	8.41E-25	5.57E-24	45.6131289
JOSD1	-1.019086366	7.090720922	8.80E-25	5.82E-24	45.5671986
GNS	-1.153706648	7.391104978	9.24E-25	6.10E-24	45.5184612
TP53	-1.117344565	5.105600948	9.28E-25	6.13E-24	45.5135917
ASCC3	-1.017224558	3.780549363	9.76E-25	6.43E-24	45.463494
NUPL1	-1.118059157	4.990463255	9.89E-25	6.51E-24	45.4504735
FAM98B	-1.005647779	3.755069568	1.01E-24	6.63E-24	45.4313771
BTBD11	-1.158048694	4.675629573	1.01E-24	6.66E-24	45.4259158
APPL1	-1.771409565	4.766978311	1.02E-24	6.70E-24	45.4198805
SF3B4	-1.0490647	7.866923708	1.04E-24	6.82E-24	45.400329
MRPL49	-1.036556854	7.435682834	1.06E-24	6.93E-24	45.3841583
LOC440434	-1.046788123	6.988263082	1.06E-24	6.94E-24	45.3826466
GRHPR	-1.00585912	6.708829389	1.08E-24	7.06E-24	45.3650063
SAC3D1	-1.337775107	4.998937185	1.11E-24	7.24E-24	45.3378674
FCRLB	-1.02080781	4.208282836	1.17E-24	7.64E-24	45.2824334
PRRC1	-1.368204784	6.00269443	1.17E-24	7.65E-24	45.2808592
DKK4	1.011690761	4.405935735	1.18E-24	7.71E-24	45.2722803
RBBP4	-1.468473169	6.204168382	1.18E-24	7.72E-24	45.2707195
NME8	-1.431576628	4.184115126	1.20E-24	7.83E-24	45.2552534
WDR47	-2.159366006	4.521833105	1.20E-24	7.85E-24	45.2527617
ZNF426	-1.340376634	4.256651887	1.20E-24	7.85E-24	45.2525104
PELI2	-1.119942837	5.437091099	1.22E-24	7.93E-24	45.2425811
STARD4	-1.072606844	3.701186565	1.27E-24	8.25E-24	45.2017664
PRMT3	-1.581555817	3.501139236	1.34E-24	8.71E-24	45.1457401
KCTD18	-1.251737432	5.461919849	1.35E-24	8.75E-24	45.1407317
CPOX	-2.227138686	3.957393469	1.37E-24	8.91E-24	45.1219791
COMMD10	-1.272159503	3.923995018	1.42E-24	9.24E-24	45.0848118
OTUD6B	-2.129875013	3.492372879	1.45E-24	9.40E-24	45.0665959
AEBP2	-1.223754252	2.95214462	1.46E-24	9.47E-24	45.0582038
MAML1	-1.517163017	7.550798073	1.48E-24	9.57E-24	45.0467563
EGFLAM	-1.065144632	4.236491476	1.51E-24	9.77E-24	45.0247238
RAB30-AS1	-1.54521821	4.839685693	1.58E-24	1.02E-23	44.9839174
NT5C2	-1.318315328	8.665305794	1.62E-24	1.04E-23	44.9559152
LOC153577	-1.302219311	3.855038657	1.63E-24	1.05E-23	44.9500923
CTC-444N24.11	-1.836693238	4.60306132	1.64E-24	1.05E-23	44.9459488
DPYD	-1.221284574	4.661234217	1.74E-24	1.11E-23	44.8869374
ZCCHC8	-1.468425975	4.880250677	1.74E-24	1.12E-23	44.8846546
GORAB	-1.376160235	2.902001926	1.84E-24	1.18E-23	44.8305743
SAMD3	-1.005254052	4.601216805	1.85E-24	1.18E-23	44.824548
NOC2L	-1.17838093	5.332185014	1.87E-24	1.20E-23	44.8109889
OVGP1	-1.004069463	5.781277507	1.95E-24	1.25E-23	44.77106
C4orf32	-1.374086905	4.269002169	1.95E-24	1.25E-23	44.7697853

AK3	-1.995788275	5.478767932	2.02E-24	1.29E-23	44.732989
INSIG2	-1.669990028	3.653047994	2.08E-24	1.33E-23	44.7040282
CLEC2D	-1.667977496	4.586752064	2.19E-24	1.40E-23	44.6531244
GNB4	-1.502495912	5.847105466	2.20E-24	1.40E-23	44.6514138
NAA35	-1.17132605	5.022150549	2.30E-24	1.46E-23	44.6053922
TBX21	-1.461473952	6.64220489	2.30E-24	1.46E-23	44.6033229
OMA1	-2.087776878	4.680404523	2.35E-24	1.49E-23	44.5834543
SLC25A32	-1.978408929	4.424555803	2.36E-24	1.50E-23	44.5788293
TACC3	-1.353486767	7.032053341	2.38E-24	1.51E-23	44.5711467
RBM3	-1.490203965	5.488630727	2.39E-24	1.52E-23	44.5657871
SMAD2	-1.127721233	5.50000823	2.50E-24	1.58E-23	44.5235101
SLC35A5	-2.22494673	4.658704931	2.51E-24	1.59E-23	44.5173602
TNFAIP3	-1.336461602	7.498096802	2.63E-24	1.66E-23	44.4692875
WDR45B	-1.823549051	7.371506267	2.68E-24	1.69E-23	44.4530822
MTF2	-1.439428104	5.222283989	2.72E-24	1.72E-23	44.4357445
C2orf42	-1.235835641	5.616675777	2.87E-24	1.81E-23	44.382565
MED4	-1.766582806	3.776959681	2.97E-24	1.86E-23	44.3496404
PPWD1	-1.501075189	3.667364807	2.98E-24	1.87E-23	44.3454312
THUMPD3-AS1	-1.02604415	4.990822936	2.99E-24	1.87E-23	44.3435465
GTF2H3	-1.269716888	5.270037431	3.00E-24	1.88E-23	44.3381684
TOX2	-1.27549662	2.954457803	3.04E-24	1.91E-23	44.3244403
PRPF38B	-1.304923464	7.004627174	3.13E-24	1.96E-23	44.2973155
DANCR	-1.283244788	6.247394223	3.18E-24	1.99E-23	44.2802041
MPHOSPH10	-1.291861673	5.25178192	3.30E-24	2.06E-23	44.2448519
BUB3	-1.189021826	5.519476874	3.30E-24	2.06E-23	44.2435941
NUAK1	-1.017164251	3.982585117	3.35E-24	2.09E-23	44.2299058
ZNF420	-1.306063583	4.069194801	3.38E-24	2.11E-23	44.2187367
CUL4B	-1.422758888	5.525108042	3.46E-24	2.16E-23	44.1972906
UBE2K	-1.104184341	6.238680933	3.46E-24	2.16E-23	44.1960078
PANK3	-1.572930579	4.190674599	3.56E-24	2.22E-23	44.1674644
TMED5	-1.225126696	4.384010827	3.60E-24	2.24E-23	44.1573613
ZBTB44	-1.489657665	5.740758522	3.64E-24	2.26E-23	44.1463659
KIAA0754	-1.323345042	4.78780625	3.67E-24	2.28E-23	44.1380891
NPAT	-1.051516196	4.928842173	3.95E-24	2.45E-23	44.0640888
UAP1	-1.911313932	3.758273859	4.02E-24	2.49E-23	44.0450242
SIGLEC17P	-1.707438193	5.664961041	4.11E-24	2.54E-23	44.0244711
CXorf38	-1.522644145	6.569156623	4.23E-24	2.61E-23	43.9958481
SLC30A7	-1.33127091	5.444409945	4.24E-24	2.62E-23	43.9924938
HPGD	-1.064109283	3.366458642	4.26E-24	2.63E-23	43.9873996
RNF4	-1.581104599	7.993621178	4.47E-24	2.76E-23	43.9391802
KLHL7	-1.40819264	3.984835725	4.49E-24	2.77E-23	43.9361452
ZMYM4	-1.324152793	5.035726356	4.51E-24	2.78E-23	43.9299612
CNOT7	-1.67362913	6.313598953	4.59E-24	2.83E-23	43.9127404
FOS	-2.370205661	6.206789123	4.64E-24	2.86E-23	43.9019866
NHSL2	-1.080059289	5.199760048	4.66E-24	2.87E-23	43.8975614
EED	-1.439421831	4.286761338	4.69E-24	2.89E-23	43.8919404
ATG101	-1.195672304	5.511482785	4.74E-24	2.92E-23	43.8803057
CTCF	-1.586160748	7.030621283	4.82E-24	2.96E-23	43.8646127
PDK4	-1.435087741	4.042817126	4.87E-24	3.00E-23	43.8533667
CASP8	-1.118817544	6.532848829	4.95E-24	3.04E-23	43.8369991
LOC102724967	1.008313235	6.448742732	5.08E-24	3.12E-23	43.8118922
CYB561A3	-1.086512685	7.237403683	5.09E-24	3.12E-23	43.8092724
MTMR12	-1.227140891	5.080688049	5.43E-24	3.33E-23	43.744763
ARFIP1	-1.661429724	4.771541057	5.65E-24	3.46E-23	43.7049862
LACTB	-1.579857992	5.159496676	6.10E-24	3.72E-23	43.6290297
LOC646214	-1.199319409	5.812977336	6.43E-24	3.91E-23	43.5761194
CEP120	-1.146247225	3.874710911	6.45E-24	3.92E-23	43.5721406
SELPLG	-1.019384119	8.487158554	6.51E-24	3.95E-23	43.5635429

FCRLA	-1.336979145	4.284001815	6.56E-24	3.98E-23	43.5559253
RSAD1	-1.056572632	6.799289132	6.63E-24	4.02E-23	43.5451559
CHFR	-1.003506754	6.893686811	6.66E-24	4.04E-23	43.5406677
ACADM	-2.651199612	4.482499203	6.83E-24	4.14E-23	43.5158411
PPIF	-1.32772823	7.009031767	6.95E-24	4.21E-23	43.4976081
ORAI1	-1.188348011	7.681315052	7.32E-24	4.43E-23	43.4456953
SFR1	-1.061175906	3.839118226	7.38E-24	4.46E-23	43.4376884
SREK1IP1	-1.501957693	4.4694214	7.56E-24	4.57E-23	43.4128022
TTC37	-1.553677501	4.481864724	7.66E-24	4.63E-23	43.4001464
LINC01270	-1.05368891	4.128436536	7.68E-24	4.64E-23	43.3970451
NGF	1.018333169	6.136084385	7.97E-24	4.81E-23	43.3600017
NNT-AS1	-1.531233191	3.746732253	8.05E-24	4.86E-23	43.3500216
AC016831.7	-1.014058115	3.689250702	8.10E-24	4.88E-23	43.3440041
SLC25A47	1.060695088	5.205053536	8.18E-24	4.93E-23	43.3346796
PCNX	-1.166201163	4.616825629	8.27E-24	4.98E-23	43.3240305
NUPL2	-1.178316232	5.3422701	8.29E-24	4.99E-23	43.3210446
FAM49A	-1.205418246	6.613804816	8.34E-24	5.01E-23	43.3155675
RP11-389C8.2	-1.023830935	3.686963032	8.40E-24	5.05E-23	43.3079843
RABGAP1	-1.037849298	6.903154549	8.41E-24	5.05E-23	43.3072564
CRTC3	-1.564791665	6.068053428	8.50E-24	5.11E-23	43.2958515
MTCH2	-1.058258493	6.201543392	8.66E-24	5.20E-23	43.2774412
SNX14	-1.64714366	4.159521079	8.96E-24	5.37E-23	43.2430253
GABPB2	-1.355768984	5.656678401	8.99E-24	5.38E-23	43.2405139
DUSP28	-1.152142494	6.690596588	9.08E-24	5.43E-23	43.2304351
TRDV3	-1.486922789	4.568809715	9.12E-24	5.46E-23	43.2253924
DDX60L	-1.734191604	5.012022263	9.33E-24	5.58E-23	43.2026205
ZNF587B	-1.525393477	5.417473765	9.43E-24	5.64E-23	43.1918314
PPP6R3	-1.254857245	6.525051681	9.56E-24	5.71E-23	43.1783499
GPR137B	-1.863550583	4.928701569	9.60E-24	5.73E-23	43.1747274
ADCY7	-1.263945167	6.126574749	9.94E-24	5.93E-23	43.1396754
SLC25A40	-1.857718364	4.042965362	1.05E-23	6.25E-23	43.0863527
CRYZ	-1.752929572	3.868783629	1.06E-23	6.31E-23	43.0770023
BEND2	-1.702624121	4.71207184	1.10E-23	6.55E-23	43.0374266
HERC2	-1.310148407	4.05771655	1.12E-23	6.63E-23	43.0243621
RBM23	-1.361667675	7.96452021	1.14E-23	6.79E-23	43.0005205
CDK4	-1.045981157	6.830124606	1.24E-23	7.38E-23	42.9140952
C16orf80	-1.493948436	6.568661676	1.26E-23	7.48E-23	42.8994672
KLHL6	-1.031632615	4.996068237	1.27E-23	7.53E-23	42.8924596
GPR144	1.474768515	5.365626005	1.28E-23	7.56E-23	42.8884052
ANAPC16	-1.209485762	7.959415318	1.28E-23	7.57E-23	42.8862056
TRAPPC12	-1.131783778	6.359180183	1.32E-23	7.79E-23	42.8561427
USP8	-1.393000727	6.763378655	1.34E-23	7.93E-23	42.8381228
DONSON	-1.06479766	3.539603046	1.39E-23	8.20E-23	42.8038832
TRIM33	-1.233026381	6.456836877	1.43E-23	8.41E-23	42.7771473
CASC4	-1.174631291	5.892791292	1.48E-23	8.73E-23	42.739187
WBP2	-1.272195994	8.424586066	1.58E-23	9.29E-23	42.6755315
TXNDC16	-1.793046025	3.686976848	1.67E-23	9.77E-23	42.6227673
HAUS3	-1.631428437	4.015824221	1.73E-23	1.01E-22	42.5846281
PHKB	-1.300573509	5.200045838	1.75E-23	1.02E-22	42.573434
CCDC6	-1.435588778	5.111861612	1.75E-23	1.03E-22	42.5707157
FLVCR1	-1.92495989	4.746006473	1.76E-23	1.03E-22	42.5689912
GRPEL1	-1.07237286	6.471467394	1.76E-23	1.03E-22	42.568568
SRI	-1.156548506	5.582429834	1.78E-23	1.04E-22	42.5581799
LBH	-1.913647623	7.619307634	1.80E-23	1.05E-22	42.5429816
BEX4	-1.498862319	4.32752251	1.81E-23	1.05E-22	42.5419606
IL15	-1.341978374	3.125346019	1.82E-23	1.06E-22	42.5355071
DCTN4	-1.236809141	4.809050937	1.83E-23	1.07E-22	42.5285588
ZNF566	-1.000246079	2.816597639	1.98E-23	1.15E-22	42.4518027

C12orf5	-1.713204682	4.931326891	1.98E-23	1.15E-22	42.4503662
RP11-554J4.1	-1.47511983	4.268511526	1.98E-23	1.15E-22	42.448822
RAB33A	-1.114188267	5.139914916	1.98E-23	1.15E-22	42.4478509
RCAN3	-1.647087101	5.241578658	1.98E-23	1.15E-22	42.446882
FAM214A	-1.699713412	4.579164733	2.05E-23	1.19E-22	42.4141826
USP25	-1.146310611	4.448217637	2.12E-23	1.22E-22	42.3829364
SPPL2A	-1.716470239	5.724500321	2.14E-23	1.24E-22	42.3712794
DDB2	-1.009285818	5.950283324	2.15E-23	1.24E-22	42.3655174
CDC123	-1.247294858	6.73225621	2.17E-23	1.25E-22	42.357099
OGT	-1.77597811	5.914009407	2.18E-23	1.26E-22	42.3520075
SNX13	-1.261258879	3.772836274	2.21E-23	1.28E-22	42.3381158
HPSE	-1.403837449	5.640979269	2.34E-23	1.35E-22	42.2810197
GINM1	-1.266328354	6.670239757	2.36E-23	1.36E-22	42.2738829
PCDHB12	1.157203888	4.119394265	2.40E-23	1.38E-22	42.258361
PMS2P8	-1.41631737	5.415321371	2.47E-23	1.42E-22	42.2280181
BTG2	-1.120579907	7.126784217	2.52E-23	1.45E-22	42.2074409
SWAP70	-1.644410693	4.865637884	2.53E-23	1.45E-22	42.2031924
BIRC6	-2.021740437	5.703365323	2.56E-23	1.47E-22	42.1915688
PIGF	-1.620708995	5.002875119	2.57E-23	1.47E-22	42.1866486
EFTUD1	-1.030022399	5.221534052	2.61E-23	1.49E-22	42.1737433
CXorf23	-1.151084835	2.829006079	2.65E-23	1.51E-22	42.1572845
LOC202025	-2.178931233	5.258872601	2.66E-23	1.52E-22	42.1541821
ZNF587	-1.551703838	6.533400295	2.70E-23	1.54E-22	42.1396664
CCDC115	-1.506642084	6.915073199	2.74E-23	1.56E-22	42.1245505
CYLD	-1.183717702	5.655210104	2.76E-23	1.57E-22	42.1175273
ZFP62	-1.838639774	4.023886604	2.84E-23	1.62E-22	42.0892746
CALHM2	-1.090649986	7.133093601	2.88E-23	1.64E-22	42.0742792
SLMO2	-1.364077161	4.976280809	2.98E-23	1.70E-22	42.0397709
RPGR	-1.126025242	3.20233494	3.04E-23	1.73E-22	42.0208639
CRYBG3	-1.138509594	4.874613151	3.06E-23	1.74E-22	42.013153
ILKAP	-1.060852398	5.621478631	3.08E-23	1.75E-22	42.0063464
AC007401.2	-1.416649051	4.194588922	3.17E-23	1.80E-22	41.978263
TYW1	-1.06429251	5.59015873	3.20E-23	1.82E-22	41.9687209
POC5	-1.025503497	5.048620368	3.22E-23	1.82E-22	41.964022
ANP32A	-1.203583726	6.581377355	3.25E-23	1.84E-22	41.9543846
KIAA0196	-1.073195142	5.942692368	3.27E-23	1.85E-22	41.9469117
CHIC2	-1.837207635	6.910648668	3.31E-23	1.87E-22	41.9359597
CCDC47	-1.415808966	4.723797124	3.35E-23	1.90E-22	41.9220585
USP9X	-1.214476078	6.674302043	3.53E-23	1.99E-22	41.8716877
STX12	-1.332168721	6.417069209	3.53E-23	1.99E-22	41.8711211
KPNA1	-1.145666021	5.881235955	3.53E-23	1.99E-22	41.8697589
ZBTB11	-2.178207494	4.471022333	3.53E-23	1.99E-22	41.8693876
PTPN4	-1.136388872	4.562999507	3.75E-23	2.11E-22	41.8086813
TMPO	-1.266293295	4.692652761	3.77E-23	2.12E-22	41.8054049
ZHX1	-1.234883207	3.480905949	3.77E-23	2.12E-22	41.8036071
PPP1R16B	-1.180857531	6.411460431	3.77E-23	2.12E-22	41.803492
CD19	-1.22107592	5.93060905	3.77E-23	2.12E-22	41.8033473
ZNF518A	-1.161131885	5.175376742	4.14E-23	2.32E-22	41.7104089
NECAP1	-1.881898415	5.79246289	4.18E-23	2.35E-22	41.7005393
ITGAL	-1.504735301	8.042396437	4.21E-23	2.36E-22	41.6933372
SLC26A2	-1.182138426	5.372753442	4.41E-23	2.47E-22	41.6468303
DPH7	-1.010557784	5.43703269	4.46E-23	2.50E-22	41.6359287
HOXB2	-1.448203167	6.62161486	4.85E-23	2.71E-22	41.5514507
ARPC1A	-1.032236353	6.572856062	4.95E-23	2.76E-22	41.5321085
MRPL10	-1.292890093	6.959423234	5.05E-23	2.82E-22	41.5112786
ZNF264	-1.560663864	5.819005763	5.12E-23	2.86E-22	41.4974752
LINS	-1.20591778	5.803120531	5.13E-23	2.86E-22	41.4965334
RHBDF2	-1.181771001	6.484996661	5.14E-23	2.86E-22	41.4951637

KLHL21	-1.167083072	6.494465691	5.15E-23	2.87E-22	41.4921537
COA5	-1.445834438	5.09427304	5.19E-23	2.89E-22	41.4846337
KIAA0907	-1.52529023	6.24953223	5.19E-23	2.89E-22	41.4846128
LOC153682	-1.642031981	4.141332972	5.25E-23	2.92E-22	41.4733412
ZNF431	-1.061147662	3.913946993	5.27E-23	2.93E-22	41.4686253
C1orf64	1.173259119	4.922411091	5.41E-23	3.00E-22	41.4438535
PRPF39	-1.624760295	3.660165707	5.41E-23	3.00E-22	41.443467
ATAD1	-2.034487806	4.44428534	5.83E-23	3.22E-22	41.3674413
HEATR5A	-2.250413768	3.928101217	5.84E-23	3.23E-22	41.3666798
NUP43	-1.100960392	5.34747337	5.85E-23	3.23E-22	41.3639394
HAUS6	-1.188900868	2.993909109	5.90E-23	3.26E-22	41.3555489
ANKS1A	-1.148456316	5.987255041	5.95E-23	3.28E-22	41.3485952
PYROXD1	-1.779228048	5.127754651	5.98E-23	3.30E-22	41.3432259
HELB	-1.25806412	5.660057227	6.04E-23	3.33E-22	41.3334126
SIMC1	-1.362989109	5.065433655	6.09E-23	3.36E-22	41.3239287
TAPT1-AS1	-2.022869877	5.679378027	6.14E-23	3.38E-22	41.3160781
TMEM50B	-1.008326931	5.215657095	6.26E-23	3.45E-22	41.2966359
ACD	-1.186969602	5.948102015	6.34E-23	3.49E-22	41.2838978
EIF3A	-1.544714436	6.435655486	6.57E-23	3.61E-22	41.2481118
CCNYL1	-1.279522671	4.154392178	6.60E-23	3.63E-22	41.2437763
YJEFN3	1.14510183	4.942374128	6.62E-23	3.63E-22	41.2412234
PXYLP1	-1.188130445	4.17707435	6.65E-23	3.65E-22	41.236752
C20orf197	-1.268646485	2.754629915	6.82E-23	3.74E-22	41.211435
SLC18A3	1.009923168	4.668944911	7.03E-23	3.85E-22	41.1803273
ENOSF1	-1.159985028	5.413588846	7.62E-23	4.16E-22	41.1000758
NSUN2	-1.10320339	6.841774699	7.68E-23	4.19E-22	41.0921052
PRKRA	-1.35054096	5.362657094	7.79E-23	4.25E-22	41.0779496
ARHGAP18	-1.149517929	4.616725178	8.07E-23	4.40E-22	41.0432462
COG5	-1.314674932	5.270226094	8.24E-23	4.49E-22	41.0214265
ERLIN1	-1.31149001	5.53110066	8.64E-23	4.70E-22	40.9741507
ZBTB2	-1.762034622	3.988136992	8.79E-23	4.78E-22	40.9571986
FAM169A	-1.435176476	3.812879358	8.84E-23	4.80E-22	40.9518413
DIS3L	-1.238831252	4.778186428	8.88E-23	4.82E-22	40.9465638
AGPS	-1.200228339	4.19744234	8.94E-23	4.85E-22	40.9397571
UNG	-1.270396561	4.412670399	9.21E-23	4.99E-22	40.9104898
SLFN5	-1.342557993	5.88397554	9.44E-23	5.11E-22	40.8860367
SLC27A3	-1.408128324	6.254627247	9.45E-23	5.12E-22	40.8852148
KAT7	-1.018178016	7.294776405	9.53E-23	5.16E-22	40.8757747
KCNE3	-1.4213486	6.578310035	9.63E-23	5.22E-22	40.8654605
ARFGEF1	-1.632320168	5.993447594	9.68E-23	5.24E-22	40.8607631
PAFAH1B1	-1.157727732	5.984099622	1.02E-22	5.49E-22	40.8124183
RHOQ	-1.378707209	6.899651115	1.02E-22	5.53E-22	40.804184
LA16c-381G6.1	1.140799533	5.21483304	1.04E-22	5.59E-22	40.7933872
TMEM57	-1.022360393	4.540686369	1.06E-22	5.69E-22	40.7741574
ETNK1	-1.454502337	3.445944575	1.08E-22	5.80E-22	40.7546671
FGR	-1.437274327	8.975653829	1.09E-22	5.87E-22	40.7420482
RBL2	-1.574207759	7.488335325	1.10E-22	5.90E-22	40.7357781
PKIA	-1.034524333	3.876721573	1.10E-22	5.91E-22	40.7347787
RALGPS2	-1.07257389	4.079843333	1.10E-22	5.91E-22	40.7345726
PTPRU	1.113285648	5.965056851	1.11E-22	5.96E-22	40.7249393
PHF11	-1.083342489	7.574822007	1.12E-22	6.03E-22	40.7127999
SLC9A8	-1.051697031	6.33023477	1.13E-22	6.06E-22	40.7079804
GMCL1	-2.451516041	5.295391029	1.18E-22	6.34E-22	40.6609683
HADHB	-1.311117552	8.228644423	1.18E-22	6.35E-22	40.6588439
PCF11	-1.083233792	6.174804108	1.19E-22	6.38E-22	40.6527554
PCYOX1L	-1.051086075	6.389156161	1.21E-22	6.46E-22	40.6404166
TSPYL4	-1.891425527	5.883409114	1.21E-22	6.50E-22	40.6331679
IMP3	-1.092890728	6.789541435	1.25E-22	6.67E-22	40.6066361

TBCK	-1.536239784	5.366709818	1.26E-22	6.73E-22	40.5975056
LRIF1	-2.086535493	3.857872122	1.26E-22	6.74E-22	40.5955169
MITD1	-2.117373859	4.992247551	1.29E-22	6.90E-22	40.5702445
MGAT4A	-1.256972496	4.251445968	1.29E-22	6.91E-22	40.5693543
PCSK7	-1.358791158	6.685515685	1.30E-22	6.91E-22	40.5692701
PTGER2	-1.952013348	5.519449917	1.32E-22	7.04E-22	40.5496711
ZAP70	-1.449559775	7.039625383	1.37E-22	7.30E-22	40.5105786
ZNF260	-1.554056131	3.949485125	1.39E-22	7.37E-22	40.5006389
LOC100287497	-1.290692494	4.516223779	1.39E-22	7.40E-22	40.4969387
GPN3	-1.641274098	4.42192228	1.42E-22	7.56E-22	40.4743172
ZNF160	-1.085237383	5.581574904	1.45E-22	7.71E-22	40.455104
CNIH1	-2.046006438	5.314683868	1.46E-22	7.74E-22	40.4501895
SLAMF6	-1.106704132	7.428229943	1.48E-22	7.82E-22	40.4389983
KIAA1598	-2.063409968	4.656885195	1.49E-22	7.88E-22	40.431362
SMARCA5	-1.936405	3.846043571	1.50E-22	7.93E-22	40.4247978
TIMM22	-1.031510323	5.365621874	1.50E-22	7.97E-22	40.4200756
SRRM1	-1.180858455	7.536381702	1.52E-22	8.03E-22	40.4116469
UFM1	-1.393479181	4.380259641	1.54E-22	8.13E-22	40.3984379
LOC728613	-1.306654864	4.646815665	1.64E-22	8.64E-22	40.3348595
CD1D	-1.946447841	5.883821394	1.64E-22	8.65E-22	40.3333801
HNRNPA1	-1.106077548	6.110004848	1.64E-22	8.65E-22	40.3333429
TUFT1	-1.199385231	4.499840467	1.67E-22	8.79E-22	40.3167516
DAB2	-1.015670258	4.052957998	1.74E-22	9.17E-22	40.274042
ZBTB26	-1.020850512	3.563807415	1.78E-22	9.34E-22	40.2534552
BARX1	1.113514507	4.609825676	1.78E-22	9.34E-22	40.2533591
CHAMP1	-1.174815809	5.92510948	1.83E-22	9.59E-22	40.2254023
LCORL	-1.644487292	4.011983124	1.85E-22	9.69E-22	40.2138913
SRP54	-1.612128647	6.025966096	1.85E-22	9.70E-22	40.213235
MALT1	-1.776916482	4.282638532	1.94E-22	1.01E-21	40.1654375
MTR	-1.500877712	4.605121196	1.94E-22	1.02E-21	40.1629857
GPX3	-1.103766299	4.713978685	1.99E-22	1.04E-21	40.1416404
ERMARD	-1.213284277	5.34985054	2.05E-22	1.07E-21	40.1074021
TOPBP1	-1.776509793	5.609219156	2.17E-22	1.13E-21	40.0522835
CRIPAK	-1.278552282	6.693771391	2.23E-22	1.16E-21	40.0264677
SPTSSA	-1.407310564	4.392206459	2.37E-22	1.23E-21	39.9648873
PWP1	-1.018254471	6.56932103	2.42E-22	1.25E-21	39.9449169
PSMD12	-1.855619061	5.578710286	2.44E-22	1.27E-21	39.9339165
IKZF5	-1.280989729	4.240554471	2.53E-22	1.31E-21	39.9007049
C3orf38	-1.348130836	4.385455099	2.53E-22	1.31E-21	39.8987598
TIA1	-1.430259392	4.951371693	2.53E-22	1.31E-21	39.8979826
CHD2	-1.082977189	5.115040252	2.54E-22	1.31E-21	39.8968406
CAMTA2	-1.001627276	6.924404135	2.56E-22	1.32E-21	39.8885724
PRKD3	-1.10295998	4.546912291	2.56E-22	1.32E-21	39.8879156
NAA16	-1.656405451	4.830130085	2.56E-22	1.33E-21	39.8856944
TAF13	-1.083016049	3.419368771	2.64E-22	1.36E-21	39.8575993
AASDHPPT	-1.43369444	4.152568495	2.69E-22	1.39E-21	39.8373656
LOC100505715	-1.105131954	5.717345672	2.69E-22	1.39E-21	39.8373056
CDK2AP1	-1.838258384	6.829199965	2.72E-22	1.40E-21	39.8271355
EIF2AK3	-1.048460245	3.979452782	2.73E-22	1.40E-21	39.8230211
UBQLN2	-1.806996549	5.731862971	2.94E-22	1.51E-21	39.7494433
DCP1A	-1.033372982	6.735611798	2.95E-22	1.51E-21	39.7462273
SNX29P2	-1.201765475	4.802252404	2.98E-22	1.53E-21	39.734476
EDEM3	-1.624414751	5.755088031	3.01E-22	1.55E-21	39.7233938
FASTKD3	-2.052478046	4.137014784	3.05E-22	1.56E-21	39.7127999
MRPS6	-1.342094811	7.081903871	3.07E-22	1.57E-21	39.7044453
NDUFA5	-1.741084223	3.812802051	3.34E-22	1.71E-21	39.6199384
INSIG1	-1.21917257	5.616653005	3.41E-22	1.74E-21	39.5999549
PFN1	-1.329539328	11.23493794	3.49E-22	1.78E-21	39.5773274

FEM1C	-1.631518755	4.049975816	3.56E-22	1.81E-21	39.5578197
PAXIP1-AS1	-1.340497884	4.30570271	3.66E-22	1.87E-21	39.5297134
PDHB	-1.13287791	6.608783277	3.73E-22	1.90E-21	39.511651
SLFN13	-1.021329809	4.071730448	3.85E-22	1.96E-21	39.4784708
PRPSAP2	-1.149785416	6.42327138	3.90E-22	1.98E-21	39.4653586
FEM1B	-1.067720441	4.16988	4.20E-22	2.14E-21	39.3904558
LIAS	-1.291130361	4.305661746	4.33E-22	2.20E-21	39.3606103
FAM122B	-2.134049947	4.303101696	4.38E-22	2.22E-21	39.3496741
LMTK3	-1.019278641	3.685923398	4.42E-22	2.24E-21	39.3405765
HINT3	-1.164928134	3.440222341	4.47E-22	2.26E-21	39.3300803
APPL2	-1.760815724	4.88263194	4.51E-22	2.29E-21	39.3193672
ZNF655	-1.540159837	5.412389832	4.55E-22	2.30E-21	39.3122005
NAA25	-1.23049221	5.1698936	4.64E-22	2.35E-21	39.2922758
NFU1	-1.560732589	3.99183661	4.81E-22	2.43E-21	39.2564669
TSN	-1.087211054	5.176397298	4.83E-22	2.44E-21	39.2524398
KDM3B	-1.459988974	6.815949087	4.85E-22	2.45E-21	39.2470983
DNAJC15	-1.001761214	4.811465754	5.01E-22	2.53E-21	39.215015
YWHAG	-1.696421122	7.509902371	5.19E-22	2.62E-21	39.1793995
LSP1	-1.456657384	9.642898198	5.20E-22	2.62E-21	39.1785064
RAB21	-1.2823	3.909097067	5.26E-22	2.65E-21	39.1661682
CABIN1	-1.067125105	7.505632859	5.50E-22	2.76E-21	39.1211566
EXOC5	-1.474360959	3.936514625	5.62E-22	2.82E-21	39.1009243
RAB5A	-1.272549956	5.446964975	5.62E-22	2.82E-21	39.1009059
MRPS30	-1.030317325	4.826997626	6.10E-22	3.05E-21	39.0185668
MAP3K8	-1.384741117	5.472648325	6.19E-22	3.09E-21	39.0033401
FYN	-1.273254733	7.949554569	6.25E-22	3.12E-21	38.9930696
ZNF721	-1.087616965	5.681435673	6.55E-22	3.27E-21	38.9464042
GAA	-1.004619927	6.782073903	6.63E-22	3.31E-21	38.9351744
EMB	-1.116778782	5.925185169	6.89E-22	3.43E-21	38.8957649
RNASE4	-1.095909474	3.478185145	6.98E-22	3.48E-21	38.8825591
PPP1R18	-1.159871516	9.829745221	7.12E-22	3.54E-21	38.863851
C5orf28	-1.712343586	4.37016969	7.14E-22	3.56E-21	38.8598393
ITPRIPL1	-1.172953841	3.966805051	7.82E-22	3.88E-21	38.7693185
DDIT4	-1.820861274	6.562150176	8.17E-22	4.05E-21	38.7259642
BC062753	-1.168472189	4.404041704	8.28E-22	4.11E-21	38.7121075
GNE	-1.325217497	4.767771809	8.34E-22	4.14E-21	38.7046829
EID2	-1.266902146	4.493090149	8.51E-22	4.21E-21	38.6851612
UQCRC2	-1.06837011	6.907519765	8.58E-22	4.25E-21	38.6761244
CTD-2292M16.8	-1.574026792	3.673240726	8.64E-22	4.28E-21	38.6700463
PSMD5-AS1	-1.022429032	4.388043021	8.68E-22	4.30E-21	38.6646629
FAM179B	-1.682614985	3.430759071	8.85E-22	4.38E-21	38.6452021
KDELR2	-1.207425297	6.210730168	8.94E-22	4.42E-21	38.6356002
ARMCX2	-1.172672458	3.614044953	9.11E-22	4.50E-21	38.6164147
PRCC	-1.116426219	5.376487005	9.18E-22	4.53E-21	38.6095009
MAN1C1	-1.102384591	5.157831665	9.24E-22	4.56E-21	38.6029212
SPIN1	-1.697118882	5.122666924	9.35E-22	4.61E-21	38.5905395
PGRMC2	-1.018823479	4.902540825	9.48E-22	4.67E-21	38.5764667
UXS1	-1.315616344	5.913632431	9.55E-22	4.70E-21	38.5699269
USP38	-1.864569896	4.535745219	9.74E-22	4.79E-21	38.5497921
SPEN	-1.047317724	6.257476932	9.84E-22	4.84E-21	38.5394993
QKI	-1.358549977	5.806637237	1.01E-21	4.95E-21	38.5164483
FOPNL	-1.486552758	4.507705101	1.01E-21	4.96E-21	38.5146728
LRPPRC	-1.302745105	5.752483758	1.01E-21	4.96E-21	38.5146544
ANKRA2	-2.35933432	4.407653601	1.01E-21	4.98E-21	38.5093238
ALG6	-1.713431807	5.851485013	1.03E-21	5.06E-21	38.4941131
TRMT2B	-1.453461543	5.842622805	1.10E-21	5.37E-21	38.4314803
ARFGAP3	-1.705041641	6.429703727	1.13E-21	5.51E-21	38.4052461
CLEC4C	-1.219070481	3.633857104	1.19E-21	5.82E-21	38.349656

PCNP	-1.560493264	5.493852558	1.22E-21	5.98E-21	38.3206209
RP11-769O8.3	-1.503135433	4.014124538	1.25E-21	6.08E-21	38.303092
EZR	-1.059991225	5.916523354	1.25E-21	6.10E-21	38.2999102
THBD	-1.147507753	5.037613356	1.26E-21	6.13E-21	38.2944334
SULF2	-1.479440808	7.057961173	1.27E-21	6.20E-21	38.2820413
PIK3CA	-1.51054288	4.58600327	1.32E-21	6.41E-21	38.2474184
KBTBD2	-1.478733773	5.664817904	1.34E-21	6.52E-21	38.2299914
ZNF354A	-1.457764045	4.749485905	1.36E-21	6.59E-21	38.2192822
LENG1	1.020217364	5.621860356	1.36E-21	6.63E-21	38.2132704
SERBP1	-1.242763353	6.980171492	1.41E-21	6.85E-21	38.1791355
MIS18BP1	-1.174572478	3.89035499	1.44E-21	7.00E-21	38.1560376
LOC439911	-1.403852263	5.745372231	1.44E-21	7.00E-21	38.1559487
ZCCHC7	-1.147850803	5.558165625	1.46E-21	7.10E-21	38.141246
C12orf75	-1.813140647	5.713676072	1.48E-21	7.16E-21	38.1331732
LOC644656	-1.040907618	5.006070863	1.49E-21	7.23E-21	38.1227235
LCLAT1	-1.655222615	5.492973772	1.56E-21	7.57E-21	38.0753697
AMD1	-1.782429072	6.213742654	1.62E-21	7.84E-21	38.0383324
WDR11	-1.475924559	6.488056998	1.65E-21	7.95E-21	38.0240987
ATM	-1.17495449	6.128707146	1.67E-21	8.04E-21	38.0122465
MAP3K4	-1.257627367	6.486319985	1.68E-21	8.12E-21	38.0013276
WDSUB1	-1.707263526	4.166629783	1.71E-21	8.24E-21	37.9856153
THUMPD1	-1.835538335	5.43114426	1.72E-21	8.29E-21	37.9795365
MTO1	-1.429146933	4.86126046	1.80E-21	8.65E-21	37.9360552
NAPB	-1.316727859	4.686302358	1.82E-21	8.73E-21	37.9257783
CSNK1A1	-1.0807104	5.999256278	1.91E-21	9.18E-21	37.8752636
ISOC1	-2.396394689	4.792039751	1.92E-21	9.22E-21	37.8709086
CCR6	-1.310799834	5.469973775	2.01E-21	9.62E-21	37.8265249
RBM22	-1.295833371	7.264777212	2.03E-21	9.71E-21	37.8166144
ARMCX3	-1.444807795	4.752654734	2.05E-21	9.83E-21	37.8042877
RSL1D1	-1.187200589	5.943715383	2.11E-21	1.01E-20	37.775667
MICB	-1.358954407	7.410252036	2.29E-21	1.09E-20	37.6941578
CERK	-1.318190809	7.226833667	2.39E-21	1.14E-20	37.6520268
VTA1	-1.386275975	4.776701612	2.41E-21	1.15E-20	37.6438272
SETD2	-1.06541889	5.549508314	2.55E-21	1.21E-20	37.5871499
ANXA4	-1.560139574	6.452952711	2.57E-21	1.22E-20	37.579989
TTI1	-1.088634277	6.433490804	2.60E-21	1.23E-20	37.5688347
TPK1	-1.791644917	4.901122813	2.66E-21	1.26E-20	37.5462304
EDEM1	-1.38122417	7.034559749	2.69E-21	1.28E-20	37.5316495
RSBN1L	-1.260239756	5.625852021	2.82E-21	1.34E-20	37.4860231
TRIM28	-1.411691274	7.965994816	2.86E-21	1.35E-20	37.4716861
BDP1	-1.270804015	3.886182568	2.86E-21	1.36E-20	37.4704734
FAM76B	-1.071080187	3.24161615	2.88E-21	1.36E-20	37.4643681
REEP3	-1.17924612	3.190880251	2.95E-21	1.40E-20	37.4404648
RAD51-AS1	-1.269232365	5.674743326	3.08E-21	1.46E-20	37.3964366
CECR5	-1.21916277	4.995928674	3.11E-21	1.47E-20	37.3873152
TAGAP	-1.527072869	7.956161812	3.14E-21	1.48E-20	37.377601
EAPP	-1.556588302	6.399855032	3.28E-21	1.54E-20	37.3343797
PEX2	-1.107491604	4.474065356	3.32E-21	1.56E-20	37.3236896
SHPRH	-2.389647914	3.617172405	3.35E-21	1.57E-20	37.3144603
SPCS1	-1.455776032	8.713361592	3.36E-21	1.58E-20	37.3104597
EIF1AX	-1.798006438	5.46073661	3.49E-21	1.64E-20	37.2713605
RFX7	-1.38374812	4.192654397	3.58E-21	1.68E-20	37.2464643
TMEM173	-1.022839987	7.461015135	3.70E-21	1.73E-20	37.2151229
ZC3H7A	-1.595595577	5.545600469	3.70E-21	1.73E-20	37.2135786
PRPS1	-1.149520541	5.731967376	3.71E-21	1.74E-20	37.2119461
SLC25A11	-1.08537241	6.663134591	3.79E-21	1.77E-20	37.1906257
DENND3	-1.352106177	7.148998675	3.96E-21	1.85E-20	37.1474745
SNX18	-1.186700736	6.147773201	3.96E-21	1.85E-20	37.1468912

ADSS	-1.006830028	4.141279593	4.04E-21	1.88E-20	37.1262253
SRSF10	-1.601058278	4.762049093	4.08E-21	1.90E-20	37.116336
CYP27A1	-1.080116407	7.317451798	4.37E-21	2.03E-20	37.0474197
NIPBL	-1.323867712	6.399179578	4.48E-21	2.08E-20	37.0225047
CTA-250D10.23	-1.046919232	5.219254534	4.50E-21	2.09E-20	37.0174451
MORF4L2	-1.424797444	5.287744662	4.61E-21	2.14E-20	36.9942335
ORC2	-1.237233044	5.414650277	4.64E-21	2.15E-20	36.9876109
RANBP2	-1.647917361	5.306170091	4.69E-21	2.18E-20	36.9776146
SMAD5	-1.120038761	4.33612054	4.75E-21	2.20E-20	36.9642387
MRPS9	-1.071238234	4.949136753	4.84E-21	2.24E-20	36.9459116
KIFAP3	-1.356749665	5.282413931	4.89E-21	2.26E-20	36.9360538
TBCB	-1.002247982	7.456485347	5.05E-21	2.34E-20	36.9023036
AKIRIN1	-1.252417692	6.244317623	5.13E-21	2.37E-20	36.8872939
CDC26	-1.24643439	6.95633588	5.21E-21	2.41E-20	36.8712403
PHOSPHO2	-1.620592228	3.568771366	5.25E-21	2.42E-20	36.864592
KBTBD7	-1.695676121	3.904993599	5.28E-21	2.44E-20	36.8588325
ZFP36L2	-1.342659199	7.159578671	5.31E-21	2.45E-20	36.8530504
BRAF	-1.076965537	4.970019016	5.55E-21	2.56E-20	36.8080561
HSPB11	-1.062034339	4.394862577	5.57E-21	2.56E-20	36.8057393
PDK3	-1.008917706	6.350221032	5.57E-21	2.57E-20	36.8040984
GPR25	-1.043630897	3.90828536	5.82E-21	2.68E-20	36.7613171
BCL9L	-1.036107363	7.095942763	5.89E-21	2.71E-20	36.7495092
THAP1	-1.772460877	4.054570482	5.90E-21	2.71E-20	36.7474875
POT1	-1.395178542	4.302472744	5.91E-21	2.72E-20	36.7454299
RP11-473I1.9	-1.024019976	5.7829859	5.93E-21	2.73E-20	36.7421583
ABAT	-1.25039402	5.452093362	5.99E-21	2.75E-20	36.7329778
LRBA	-1.159545807	5.893587662	6.14E-21	2.82E-20	36.7078593
MRFAP1	-1.875851602	8.734730482	6.18E-21	2.84E-20	36.7002589
LINC00339	-1.257045343	5.301078328	6.41E-21	2.94E-20	36.6646135
LDLRAP1	-1.08252709	8.190874945	6.56E-21	3.00E-20	36.6414209
HEATR5B	-1.490077594	5.690907032	6.57E-21	3.01E-20	36.6394425
LOC389765	-1.418202059	3.943496115	6.69E-21	3.06E-20	36.6223105
ACTL6A	-1.850633257	5.026963086	6.80E-21	3.11E-20	36.6046381
PGGT1B	-1.389452152	3.828373715	6.82E-21	3.12E-20	36.60279
TMED10	-1.110351581	6.881919026	6.82E-21	3.12E-20	36.6019087
GLOD4	-1.685579405	5.077888223	6.91E-21	3.16E-20	36.589851
VWA9	-1.010852129	5.078185322	6.94E-21	3.17E-20	36.5847423
PNISR	-1.408363301	6.975893622	6.98E-21	3.19E-20	36.5795252
RPAP3	-1.529877198	4.010437933	7.16E-21	3.27E-20	36.5532721
PRF1	-1.631852771	8.908477231	7.19E-21	3.28E-20	36.5501054
EMC2	-1.757005073	3.162974462	7.44E-21	3.39E-20	36.5146562
CORO1C	-1.382495092	7.371817063	7.48E-21	3.41E-20	36.5101597
KLRC3	-1.771740802	3.607373566	7.50E-21	3.41E-20	36.5073862
SPAG9	-1.396950841	6.010800557	7.58E-21	3.45E-20	36.497088
PIK3AP1	-1.737618574	6.278401419	7.66E-21	3.48E-20	36.4866792
ARHGEF18	-1.293310952	9.207858588	7.70E-21	3.50E-20	36.4805906
ATP8B4	-1.32857403	3.086189545	7.76E-21	3.53E-20	36.4726112
ERBB2IP	-1.968879836	5.174306095	7.80E-21	3.54E-20	36.4681391
ANKMY2	-1.387951867	5.007247054	7.92E-21	3.60E-20	36.4527184
DENND2D	-1.483289407	6.842730863	8.00E-21	3.63E-20	36.4426412
SRP72	-1.239012939	5.282947697	8.11E-21	3.68E-20	36.4291883
MAK16	-1.296975391	5.34304127	8.17E-21	3.70E-20	36.4221652
XRN1	-1.425105035	4.725052451	8.28E-21	3.75E-20	36.4081802
TMEM203	-1.189873952	7.070590806	8.37E-21	3.79E-20	36.3971591
PPM1K	-1.039490582	4.802511517	8.40E-21	3.80E-20	36.3941404
RPS6KB1	-1.453802472	4.961627742	8.63E-21	3.90E-20	36.3672274
GSDMB	-1.071783055	5.488561751	8.73E-21	3.94E-20	36.3553879
AHSA2	-1.27946129	5.205457772	8.81E-21	3.98E-20	36.3459476

EEA1	-1.018138389	2.923317559	8.85E-21	4.00E-20	36.3417
CPSF6	-1.365686103	4.589731441	9.06E-21	4.09E-20	36.3186138
MCUR1	-1.222318567	4.932760943	9.22E-21	4.16E-20	36.3004563
SUCLA2	-2.275941099	3.941655403	9.45E-21	4.25E-20	36.2765425
UBLCP1	-1.844478668	6.621468032	9.63E-21	4.33E-20	36.257655
NOL7	-1.01000298	6.61086173	1.01E-20	4.55E-20	36.2075048
XRCC5	-1.072140718	7.089770614	1.02E-20	4.57E-20	36.2031087
ANKRD36B	-2.005398398	5.991391019	1.04E-20	4.68E-20	36.1780959
FLJ31306	-2.219898751	5.679775069	1.08E-20	4.84E-20	36.1425481
PRRG4	-1.388224261	6.116598992	1.12E-20	5.00E-20	36.1095738
ATR	-1.177035739	4.185154466	1.14E-20	5.09E-20	36.0896253
GLTSCR1L	-1.152949837	7.320276895	1.14E-20	5.11E-20	36.0864716
NLRC4	-1.066560768	4.538504475	1.16E-20	5.18E-20	36.0705359
ITGB3BP	-1.465064229	3.920927545	1.18E-20	5.26E-20	36.056196
MDFIC	-1.300205541	4.61819353	1.20E-20	5.34E-20	36.0398442
BROX	-1.194982799	4.666231469	1.21E-20	5.39E-20	36.0307246
LINC00028	1.09058192	5.275810939	1.21E-20	5.41E-20	36.0262861
RANBP9	-1.170833994	4.802031968	1.24E-20	5.53E-20	36.0035142
PRKY	-1.615789295	4.04330447	1.25E-20	5.56E-20	35.9983004
EBLN3	-1.913216434	6.013182154	1.29E-20	5.74E-20	35.9636867
DLEU2	-1.264573955	5.72462452	1.35E-20	5.99E-20	35.921768
LILRB1	-1.083256285	7.073699202	1.39E-20	6.17E-20	35.8901479
CEBPD	-1.227004603	6.316733086	1.41E-20	6.27E-20	35.8743789
6-Sep	-1.172676733	6.99049169	1.41E-20	6.27E-20	35.8743502
TGFBR1	-1.281731221	6.082977138	1.42E-20	6.29E-20	35.8709543
NR2C2	-1.442308913	4.996788786	1.48E-20	6.55E-20	35.8278004
POLI	-1.314443176	2.825248312	1.49E-20	6.58E-20	35.8232194
TCFL5	-1.166847675	4.490772951	1.51E-20	6.69E-20	35.8049029
FIBP	-1.006544112	7.293932595	1.53E-20	6.78E-20	35.7916128
TAPBP	-1.005937047	7.491288005	1.55E-20	6.85E-20	35.7810492
ATP11C	-1.002474391	3.487186187	1.59E-20	7.04E-20	35.7524682
MIS12	-1.70192376	5.602512118	1.63E-20	7.18E-20	35.7321982
ARRB2	-1.008947877	9.46541808	1.63E-20	7.18E-20	35.7320139
WDFY3	-1.258000926	5.457445054	1.66E-20	7.31E-20	35.7126655
KDM2B	-1.543462196	6.526643341	1.70E-20	7.47E-20	35.6901395
CYP20A1	-1.19814621	4.729190041	1.71E-20	7.52E-20	35.6825377
U2SURP	-1.479087896	4.596965438	1.72E-20	7.57E-20	35.6756252
GNAQ	-1.308264604	6.394382061	1.77E-20	7.77E-20	35.6495551
C1orf85	-1.036727061	5.332713752	1.77E-20	7.78E-20	35.6467824
LDOC1L	-1.036360291	6.061684338	1.81E-20	7.93E-20	35.6268555
ADO	-1.523297802	5.179778034	1.86E-20	8.15E-20	35.598326
RFWD3	-1.312749119	5.727517584	1.90E-20	8.30E-20	35.579788
KB-431C1.5	-1.130459374	3.927962928	1.91E-20	8.38E-20	35.5699377
CYTH4	-1.136805891	6.978892922	1.92E-20	8.43E-20	35.5647429
SMURF2	-1.321590662	5.349880562	1.93E-20	8.44E-20	35.5630981
GMNN	-1.299841054	3.982157533	1.93E-20	8.46E-20	35.5608249
SH3BP5	-1.188639838	7.947783621	1.94E-20	8.48E-20	35.557989
SMIM20	-1.309283707	4.837761419	1.94E-20	8.49E-20	35.556719
SLC12A6	-1.207689024	6.834564636	1.98E-20	8.64E-20	35.5381153
DCAF16	-1.072640835	4.547051418	2.05E-20	8.94E-20	35.5026942
PGK1	-1.145498952	7.058104375	2.06E-20	8.98E-20	35.4980265
RNF141	-1.228394612	4.751848718	2.08E-20	9.06E-20	35.4888432
CNOT8	-1.826015094	6.271705168	2.11E-20	9.19E-20	35.4730111
APOL6	-1.184289618	5.485222338	2.14E-20	9.34E-20	35.4571839
HNRNPR	-1.009100654	6.700244286	2.16E-20	9.40E-20	35.4500279
H2AFV	-1.247313822	6.384981302	2.18E-20	9.48E-20	35.4412933
SEPHS2	-1.115313477	7.979337437	2.27E-20	9.88E-20	35.3982666
CMTR2	-2.65267312	3.907286583	2.32E-20	1.01E-19	35.3788014

INTS12	-1.058380583	5.287663103	2.34E-20	1.02E-19	35.3689761
FUBP1	-1.18453372	4.725046787	2.37E-20	1.03E-19	35.3553342
DNAJB6	-1.380098952	6.2031506	2.47E-20	1.07E-19	35.3132465
FBLN5	-1.264820172	4.76035186	2.58E-20	1.11E-19	35.2725284
RBM4	-1.548193351	6.175631914	2.61E-20	1.13E-19	35.2605742
LRRC69	-1.201295463	4.92179113	2.61E-20	1.13E-19	35.2582709
FYTTD1	-2.010847702	5.929065811	2.64E-20	1.14E-19	35.2500818
GTF3C6	-1.001584254	7.81747926	2.64E-20	1.14E-19	35.2484872
SOCS4	-1.063043503	3.820658345	2.66E-20	1.15E-19	35.2415242
EIF3H	-1.138206541	6.193760818	2.67E-20	1.15E-19	35.2386785
TNFAIP8L1	-1.089703082	5.586730789	2.67E-20	1.15E-19	35.2355765
ABRACL	-1.880519051	6.670166672	2.68E-20	1.15E-19	35.233421
ARNTL	-1.423516375	6.342410183	2.74E-20	1.18E-19	35.2106032
STK38L	-1.735878796	5.191027243	2.83E-20	1.22E-19	35.1784654
AMPD2	-1.228522908	7.799632789	2.84E-20	1.22E-19	35.1759586
LOC157860	1.16960614	4.034104246	2.86E-20	1.23E-19	35.1683024
UBE2N	-1.419287966	6.465210371	2.90E-20	1.24E-19	35.1550434
HAUS1	-1.656087521	4.357028312	2.90E-20	1.25E-19	35.1533247
PHACTR2	-1.158325888	5.32791589	2.91E-20	1.25E-19	35.1504103
UBE2D1	-1.662757654	4.190374364	2.91E-20	1.25E-19	35.1493459
ZEB2	-1.200920886	5.064635274	2.92E-20	1.25E-19	35.1482672
VAMP2	-1.089581552	6.503482183	2.93E-20	1.26E-19	35.1424849
GORASP2	-1.276524295	6.765564756	2.94E-20	1.26E-19	35.1409511
TRIB2	-1.162754008	5.851955772	3.09E-20	1.32E-19	35.091845
P4HB	-1.043002294	7.187390097	3.10E-20	1.33E-19	35.0867582
JKAMP	-1.841800304	4.524949192	3.41E-20	1.45E-19	34.9937257
BLOC1S4	-1.097946625	7.15739754	3.43E-20	1.46E-19	34.9860503
PCYOX1	-1.128857849	4.762357456	3.45E-20	1.47E-19	34.9820143
GIMAP1	-1.218618817	7.187656798	3.49E-20	1.49E-19	34.9692167
7-Mar	-1.292455472	5.275304945	3.59E-20	1.53E-19	34.9410565
FPGT	-1.81183887	3.466719424	3.71E-20	1.58E-19	34.908756
HNRNPK	-1.645483493	9.072510072	3.77E-20	1.60E-19	34.8917038
CAAP1	-1.129356104	3.697441513	3.87E-20	1.64E-19	34.867017
C19orf68	1.150497161	5.569706483	4.00E-20	1.70E-19	34.8328597
CCPG1	-2.139585534	5.255961565	4.00E-20	1.70E-19	34.8316975
PPM1B	-2.010004192	5.325017354	4.13E-20	1.75E-19	34.8008668
MRPS18B	-1.0149461	6.476943405	4.15E-20	1.76E-19	34.7949101
DGCR14	1.048084086	7.106160835	4.20E-20	1.78E-19	34.7846819
SAMHD1	-1.123554217	8.20163425	4.20E-20	1.78E-19	34.7842954
MBD4	-1.401573289	6.854265271	4.23E-20	1.79E-19	34.7760257
IARS2	-2.004504691	6.434171809	4.38E-20	1.85E-19	34.7420547
TERF1	-1.11556263	5.704855989	4.42E-20	1.87E-19	34.7327546
BAG4	-1.020378641	4.489308223	4.44E-20	1.88E-19	34.7278968
LANCL1	-1.121584275	5.624765237	4.54E-20	1.92E-19	34.7059811
DENND4A	-1.346419378	4.529175027	4.73E-20	1.99E-19	34.6655459
ZNF302	-1.344037387	3.799144888	5.01E-20	2.11E-19	34.6071715
PBXIP1	-1.004255876	7.236861097	5.03E-20	2.12E-19	34.6029106
C19orf52	-1.091676719	4.1623398	5.06E-20	2.13E-19	34.5971903
XRCC6	-1.046081271	6.008855844	5.11E-20	2.14E-19	34.5886266
YWHAZ	-1.556637262	7.967045859	5.17E-20	2.17E-19	34.5757178
GBAS	-2.296965059	5.106195283	5.20E-20	2.18E-19	34.5695917
ARID2	-1.079899475	6.074933053	5.24E-20	2.20E-19	34.5634748
PIK3CG	-1.39248436	5.711463736	5.24E-20	2.20E-19	34.5631278
SNRNP27	-1.160510032	5.801572875	5.29E-20	2.22E-19	34.5526192
MAP3K2	-1.126520935	4.618334788	5.47E-20	2.29E-19	34.5199626
PDIA6	-1.383616847	6.701213464	5.53E-20	2.32E-19	34.5079676
LINC01215	-2.110496249	4.235048044	5.71E-20	2.39E-19	34.4764671
PAPD5	-1.312100967	5.704258787	5.83E-20	2.44E-19	34.4563703

TOMM70A	-1.240998529	5.89018044	5.99E-20	2.50E-19	34.4286162
DHX16	-1.032781741	6.159839664	6.19E-20	2.58E-19	34.3956377
SPIN4	-1.25976385	2.669919058	6.30E-20	2.63E-19	34.3783207
PHACTR4	-1.103346841	5.894416872	6.58E-20	2.74E-19	34.334904
DUSP11	-1.432247297	6.320473215	6.62E-20	2.76E-19	34.3291883
MAP2K1	-1.73653415	6.828035641	6.64E-20	2.76E-19	34.3256329
SYTL1	-1.172405145	8.52936234	6.77E-20	2.81E-19	34.3071648
HELZ	-1.076588454	5.460032806	6.85E-20	2.85E-19	34.2943912
CUL3	-1.249049575	5.107669214	6.91E-20	2.87E-19	34.2858159
MRPL18	-1.153809327	7.178696436	6.99E-20	2.90E-19	34.2743796
PPP3CB	-1.242898804	5.579211238	7.21E-20	2.99E-19	34.2432871
SMEK1	-1.158214981	5.792096123	7.34E-20	3.05E-19	34.2257076
RP11-732A19.1	-1.199938549	5.181881191	7.44E-20	3.09E-19	34.2117353
CCAR1	-1.43063439	4.998454768	7.55E-20	3.13E-19	34.1972743
DOCK8	-1.2837087	6.799691511	7.63E-20	3.16E-19	34.187297
GLT8D1	-1.218389255	5.626113506	7.77E-20	3.21E-19	34.1692976
ME2	-1.380079688	6.456263598	7.78E-20	3.22E-19	34.1674391
TAF12	-1.176039546	5.483304004	7.85E-20	3.24E-19	34.1586091
SLC25A45	-1.003239793	4.865588262	7.89E-20	3.26E-19	34.1537911
NR1D2	-1.846713489	5.330912689	7.91E-20	3.27E-19	34.1507601
MFSD5	-1.145091255	6.911997914	8.01E-20	3.30E-19	34.1383446
MTMR4	-1.02433543	6.54753963	8.06E-20	3.32E-19	34.1324031
POLR2E	-1.035935519	7.536359283	8.06E-20	3.32E-19	34.1322364
ETF1	-1.101020327	6.343987496	8.11E-20	3.34E-19	34.1261929
WDR82	-1.265460451	8.501129536	8.14E-20	3.36E-19	34.1220014
DDX39A	-1.209640045	6.858348477	8.22E-20	3.39E-19	34.1125539
RAB28	-1.056939835	3.615935782	8.30E-20	3.42E-19	34.1021308
PUM1	-1.352599411	7.172664956	8.41E-20	3.46E-19	34.0900341
UBE2W	-1.145917342	4.105269856	8.54E-20	3.51E-19	34.0746069
LOC100291323	-1.141800973	3.984284015	8.90E-20	3.66E-19	34.033061
DSTN	-1.062465239	5.520587104	8.92E-20	3.67E-19	34.0306601
PPCS	-1.167601798	5.893448602	9.43E-20	3.87E-19	33.9748814
EPM2AIP1	-1.266634463	5.93842654	9.52E-20	3.91E-19	33.9657399
PLAGL1	-1.605360546	5.677465551	9.77E-20	4.00E-19	33.940068
NAA50	-1.622121622	4.252966102	9.97E-20	4.09E-19	33.9195441
IRF1	-1.310864514	8.824042902	1.02E-19	4.19E-19	33.8927143
SLFN11	-1.244336714	5.087611142	1.03E-19	4.22E-19	33.885694
DDX3X	-1.204046214	5.370261346	1.04E-19	4.25E-19	33.8779487
RP11-488L18.10	-2.198121443	3.964790361	1.06E-19	4.34E-19	33.856382
TRMT6	-1.034279037	5.292552048	1.07E-19	4.37E-19	33.847888
RAB11FIP2	-1.279560545	5.381429313	1.09E-19	4.44E-19	33.8307036
NUP50	-1.016119504	5.226759211	1.10E-19	4.48E-19	33.8234193
FGD4	-1.042729989	4.628238562	1.10E-19	4.48E-19	33.8232683
TP53INP2	-1.235174968	5.791912343	1.11E-19	4.53E-19	33.8099668
TTC14	-1.647167479	4.334086479	1.13E-19	4.62E-19	33.7904551
MYO1F	-1.247045564	10.35422897	1.13E-19	4.62E-19	33.7900518
PKN2	-1.827353475	5.467616754	1.14E-19	4.65E-19	33.7827594
ZBED1	-1.089412798	6.153172926	1.15E-19	4.67E-19	33.7787681
ACVR1	-1.562086336	5.242645167	1.16E-19	4.71E-19	33.7692464
ADH5	-1.439239212	5.720268414	1.18E-19	4.80E-19	33.749544
LYPLAL1	-1.529351058	4.584515884	1.19E-19	4.81E-19	33.7463277
UBR5	-1.662463499	4.902586452	1.22E-19	4.95E-19	33.7176435
MLLT11	-1.079312528	5.634515527	1.23E-19	4.99E-19	33.7106676
ATF1	-1.743930405	3.927478169	1.23E-19	5.00E-19	33.7085384
YIPF5	-1.207859486	5.591792067	1.24E-19	5.05E-19	33.6974961
CTSC	-1.117939912	6.902472174	1.25E-19	5.06E-19	33.6955444
TSC22D3	-1.096556639	7.38966122	1.29E-19	5.23E-19	33.6610367
TMEM251	-1.901050224	5.257200258	1.34E-19	5.41E-19	33.6263907

VCPKMT	-1.476455335	6.238251036	1.38E-19	5.58E-19	33.5942017
LINC00621	-1.794486514	4.446673377	1.38E-19	5.58E-19	33.5935009
RPL7L1	-1.282608876	7.811009514	1.41E-19	5.68E-19	33.5759901
HIGD1A	-1.25523953	5.946159864	1.42E-19	5.73E-19	33.566803
PLEKHA1	-1.605466009	6.095734831	1.42E-19	5.73E-19	33.5656434
PASK	-1.034521935	5.287784732	1.43E-19	5.77E-19	33.559847
LAT	-1.030658218	7.108521358	1.44E-19	5.83E-19	33.5489796
PPP1R2	-1.152306299	5.915567124	1.49E-19	5.99E-19	33.5206975
UBXN4	-1.140557877	5.709290318	1.50E-19	6.06E-19	33.5098437
NKTR	-1.0158063	5.881647242	1.54E-19	6.21E-19	33.483724
PPAPDC2	-1.690890172	4.657338723	1.56E-19	6.30E-19	33.4695344
CCNL1	-1.105322387	5.061199025	1.58E-19	6.37E-19	33.4584387
ABHD14B	-1.017373439	6.73293558	1.62E-19	6.52E-19	33.4342496
TMX3	-1.111251427	3.821166474	1.63E-19	6.56E-19	33.4279501
VIPR1	-1.089726183	6.611335473	1.67E-19	6.71E-19	33.404412
CXorf65	-1.2018692	5.319648528	1.72E-19	6.91E-19	33.374423
SIGIRR	-1.08884676	7.097575882	1.76E-19	7.06E-19	33.3512735
OARD1	-1.06273668	6.389641052	1.80E-19	7.21E-19	33.3299949
SS18L1	-1.979082684	4.311099054	1.86E-19	7.44E-19	33.2981893
DCTD	-1.039818184	6.109913739	1.87E-19	7.48E-19	33.2920196
KLHL24	-1.027066507	5.102379671	1.90E-19	7.62E-19	33.2732632
KDM6A	-1.366986824	4.606994558	1.95E-19	7.81E-19	33.2480186
ARF4	-1.445386833	7.632961725	1.97E-19	7.89E-19	33.2370449
ZBTB41	-1.92260703	3.189552985	1.98E-19	7.91E-19	33.2332522
PGM1	-1.102348227	6.917910446	2.00E-19	7.99E-19	33.2230321
KPNA2	-1.455606582	6.665740279	2.05E-19	8.19E-19	33.197518
CHST12	-1.065087268	6.698646983	2.05E-19	8.20E-19	33.196726
PRR12	-1.057255079	5.775484214	2.08E-19	8.31E-19	33.1824222
CACNA2D3	-1.422454508	5.584502318	2.10E-19	8.38E-19	33.1735508
DHTKD1	-1.01577727	5.916137775	2.13E-19	8.48E-19	33.1608665
ZFAND6	-1.189202651	7.272048943	2.22E-19	8.85E-19	33.1173355
RRAGD	-1.383507397	6.143957786	2.25E-19	8.94E-19	33.1076036
DENND4C	-1.135321689	5.001287376	2.36E-19	9.37E-19	33.0590204
IL18R1	-1.629847151	4.131796845	2.40E-19	9.54E-19	33.0397643
TMX2	-1.009457259	7.452733779	2.40E-19	9.54E-19	33.0395203
TMEM41B	-1.681571204	5.453282263	2.42E-19	9.59E-19	33.0336328
FNTA	-1.377468622	6.163336509	2.43E-19	9.62E-19	33.0303571
ARRDC4	-1.891482979	5.298937718	2.44E-19	9.68E-19	33.0236586
LTN1	-1.145359805	4.69478964	2.61E-19	1.03E-18	32.9576728
CSGALNACT2	-1.968175532	5.187924108	2.69E-19	1.06E-18	32.9272058
GGCT	-1.193384376	5.954114052	2.87E-19	1.13E-18	32.8606205
BBX	-1.025329424	6.666515952	3.04E-19	1.19E-18	32.8061137
WSB1	-1.001462718	6.491949728	3.06E-19	1.20E-18	32.796948
PAPOLA	-1.428181437	5.979973736	3.27E-19	1.28E-18	32.7315413
CIR1	-1.040973354	5.291376428	3.28E-19	1.29E-18	32.728862
ANAPC5	-1.034506759	6.910036601	3.33E-19	1.31E-18	32.7141089
LRRC37A3	-1.269070636	5.182364289	3.33E-19	1.31E-18	32.7134172
ATP6V1C1	-1.281152411	4.741072546	3.35E-19	1.31E-18	32.7086162
TECR	-1.242263528	5.441491454	3.36E-19	1.32E-18	32.7058478
ANXA7	-1.199688651	7.982446663	3.50E-19	1.37E-18	32.6638202
RFC4	-1.26470762	4.540943195	3.66E-19	1.43E-18	32.6184673
UBE2D2	-1.068220534	6.290531977	3.74E-19	1.46E-18	32.5976605
SPAG1	-1.54439082	3.089273994	3.77E-19	1.47E-18	32.5887473
HVCN1	-1.04524094	8.668294862	3.80E-19	1.48E-18	32.5821131
NUDT19	-1.743949109	5.498263902	3.83E-19	1.49E-18	32.5748386
NLK	-1.031050053	5.062535123	3.88E-19	1.51E-18	32.561129
SUV420H1	-1.006114553	6.440697531	3.88E-19	1.51E-18	32.5605803
GK	-1.310457969	5.548404783	4.15E-19	1.62E-18	32.4924795

FAM175A	-1.190327536	3.530823258	4.22E-19	1.64E-18	32.4770794
AMIGO2	-2.120699712	4.345289445	4.26E-19	1.66E-18	32.4677974
CEP63	-1.09538605	4.904289772	4.36E-19	1.69E-18	32.4439793
RP3-368A4.6	-1.860749647	3.472429752	4.52E-19	1.75E-18	32.4086769
FAM69A	-1.01295789	4.011233728	4.56E-19	1.77E-18	32.3992535
MST4	-2.015947649	4.621173343	4.94E-19	1.91E-18	32.3200084
ZNF200	-1.377699041	5.776349378	4.95E-19	1.91E-18	32.3175048
APOBEC3C	-1.194739785	7.138499241	4.96E-19	1.92E-18	32.3144784
PRKCQ	-1.293352321	6.777616531	5.05E-19	1.95E-18	32.2974012
KLF4	-1.268553944	5.938488131	5.18E-19	2.00E-18	32.2718912
DROSHA	-1.128046804	5.756699823	5.22E-19	2.01E-18	32.2643936
PPTC7	-1.830617896	6.784581092	5.27E-19	2.03E-18	32.2553762
RBM47	-1.063788046	5.49440584	5.43E-19	2.09E-18	32.2243553
C1orf52	-1.404847628	4.861622276	5.52E-19	2.12E-18	32.208554
WTAP	-1.01142892	5.816541426	5.60E-19	2.15E-18	32.1934878
NGLY1	-1.154995248	7.147129085	5.95E-19	2.28E-18	32.1331451
RAB14	-1.078059426	5.796348441	5.98E-19	2.29E-18	32.1287852
MTHFD2	-1.085978168	4.326173109	6.35E-19	2.43E-18	32.0688341
GNPDA2	-1.066766276	3.714513773	6.40E-19	2.45E-18	32.0603498
PDHX	-1.274355666	4.589902331	6.41E-19	2.46E-18	32.0587866
NDFIP1	-1.556110698	6.160309819	6.41E-19	2.46E-18	32.058228
TCF7L2	-1.006501432	5.912758724	6.44E-19	2.47E-18	32.0541277
N4BP2L2	-1.452698167	6.476461599	6.51E-19	2.49E-18	32.0433079
PCED1B-AS1	-1.163382455	6.755765435	6.65E-19	2.54E-18	32.0220063
RAB11A	-1.45827599	5.879292755	6.80E-19	2.60E-18	31.999224
ZYX	-1.223998816	8.725195418	6.96E-19	2.66E-18	31.9769797
MAGT1	-1.20753536	6.382142323	7.13E-19	2.72E-18	31.9520684
GPD1L	-1.126479118	6.17707081	7.26E-19	2.77E-18	31.933943
ZNF14	-1.243771469	4.309051755	7.43E-19	2.83E-18	31.9115258
METTL23	-1.534517131	6.671416531	7.45E-19	2.84E-18	31.9088872
WDFY1	-1.354577657	6.073396306	7.49E-19	2.85E-18	31.9029273
MSMO1	-1.810404509	4.673022679	7.63E-19	2.90E-18	31.885175
AMPD3	-1.430892555	6.716588664	7.96E-19	3.03E-18	31.8423577
IKBIP	-1.554948973	4.509411268	8.44E-19	3.20E-18	31.7842663
HNRNPA0	-1.042286866	5.814179477	8.55E-19	3.25E-18	31.7704396
KIAA0232	-1.546163344	6.06327031	8.55E-19	3.25E-18	31.7703266
DPYSL2	-1.680740216	6.986160225	9.30E-19	3.53E-18	31.6864649
ZC3H12D	-1.474522072	6.077417443	1.03E-18	3.87E-18	31.5885551
OSTC	-1.826981911	6.544020085	1.03E-18	3.88E-18	31.5852776
LYSMD3	-2.234617319	4.752140433	1.04E-18	3.90E-18	31.5799067
SEC31A	-1.081131103	7.500942245	1.11E-18	4.19E-18	31.5056672
ANKRD12	-1.594332269	4.527954402	1.14E-18	4.27E-18	31.4868997
IL10RA	-1.335014318	8.351004897	1.17E-18	4.38E-18	31.4594998
L3MBTL3	-1.154669251	5.029308902	1.19E-18	4.48E-18	31.4369816
DCP2	-1.064939237	5.813092119	1.22E-18	4.58E-18	31.4149125
FKBP11	-1.022167185	6.644732771	1.26E-18	4.72E-18	31.3827884
ABCE1	-1.823789175	4.254959309	1.27E-18	4.74E-18	31.3779839
ABHD13	-1.125759548	2.863079065	1.29E-18	4.82E-18	31.3601429
PAM	-1.45847384	5.310924202	1.37E-18	5.11E-18	31.3006782
ARPP19	-1.340579934	5.710525871	1.40E-18	5.23E-18	31.2769232
ITGAE	-1.282426423	6.393840262	1.48E-18	5.52E-18	31.2234891
EGLN1	-1.378775495	5.88142275	1.48E-18	5.53E-18	31.220939
SECTM1	-1.185573997	9.478509831	1.50E-18	5.59E-18	31.2106195
FAM217B	-1.991479431	5.258472039	1.52E-18	5.68E-18	31.1927238
TBC1D15	-1.300494583	4.116163538	1.58E-18	5.88E-18	31.1577765
CYP11B2	1.210528012	5.804098099	1.59E-18	5.92E-18	31.1515366
BBIP1	-1.063224866	5.714497317	1.65E-18	6.14E-18	31.1142867
PRIM1	-1.106515727	5.352664996	1.67E-18	6.20E-18	31.1042528

FAM200B	-1.031651937	4.932521564	1.68E-18	6.25E-18	31.0956108
ARMC10	-1.098871744	5.376158795	1.70E-18	6.30E-18	31.0862427
UBR1	-1.243478108	4.69878915	1.71E-18	6.35E-18	31.0782821
BCAS2	-1.476637795	5.497466077	1.84E-18	6.81E-18	31.0060148
WDR36	-1.037943248	4.17697074	1.86E-18	6.88E-18	30.9951116
HLTF	-1.944779653	4.52073652	1.86E-18	6.89E-18	30.9937559
PEX11B	-1.217653083	6.709987229	1.86E-18	6.90E-18	30.9918067
RBBP8	-1.136909121	4.227593949	1.87E-18	6.92E-18	30.9896461
TP53BP2	-1.218844186	4.658782649	1.89E-18	7.00E-18	30.9772879
MRPL34	-1.229899426	5.586507341	1.96E-18	7.25E-18	30.9403339
NMD3	-1.093962491	4.231809154	1.98E-18	7.33E-18	30.9296683
ZFR	-1.136430225	5.25618114	2.03E-18	7.51E-18	30.9049546
ATG14	-1.317076334	5.792594453	2.05E-18	7.58E-18	30.8950849
DOCK10	-1.388180636	5.336546156	2.05E-18	7.58E-18	30.8950039
RECQL	-1.599972056	5.423597085	2.10E-18	7.72E-18	30.8747488
SCAF8	-2.083906536	5.944094323	2.10E-18	7.72E-18	30.8747424
PRKX	-1.28825605	5.328359937	2.11E-18	7.76E-18	30.8695764
MAP4K5	-1.313418679	4.023575687	2.13E-18	7.86E-18	30.8566048
PLEKHF2	-2.353212307	4.825745585	2.24E-18	8.23E-18	30.8084652
MOSPD2	-1.17933754	6.92904414	2.26E-18	8.32E-18	30.7974401
ZHX2	-1.063304326	6.238679047	2.29E-18	8.39E-18	30.7884326
CTR9	-1.783451681	6.227730641	2.34E-18	8.59E-18	30.7643778
WIPF1	-1.307816957	7.06253649	2.36E-18	8.65E-18	30.7572834
C14orf119	-1.022051766	7.402681381	2.39E-18	8.78E-18	30.7415005
CXorf40A	-1.047162097	6.739856198	2.42E-18	8.85E-18	30.7331464
POGLUT1	-1.278568823	5.665670286	2.47E-18	9.04E-18	30.7118985
AP5M1	-1.169544745	4.854726001	2.48E-18	9.07E-18	30.7081444
OGFRL1	-1.79313233	6.628276944	2.48E-18	9.07E-18	30.7075082
STK17B	-1.51147477	5.971360526	2.49E-18	9.12E-18	30.7016811
PIGK	-1.1073033	3.518499035	2.49E-18	9.12E-18	30.7016401
FCGRT	-1.02428148	8.778752776	2.52E-18	9.22E-18	30.6912582
ZNF140	-1.530396538	3.819840598	2.69E-18	9.81E-18	30.6270392
ZNF776	-1.660819465	5.140153736	2.75E-18	1.01E-17	30.6015632
RTN1	-1.385119172	6.072678923	2.79E-18	1.02E-17	30.5899382
AMN1	-1.224682093	4.798934197	2.86E-18	1.04E-17	30.5656762
KRBOX4	-1.086977675	4.18443708	2.86E-18	1.04E-17	30.5642651
PPAPDC1B	-1.064163671	5.299193647	2.91E-18	1.06E-17	30.5469333
SGMS1	-1.870690331	3.087202432	2.92E-18	1.06E-17	30.544755
ZNF708	-1.110902116	3.370479314	3.01E-18	1.09E-17	30.5122853
HNRNPA2B1	-1.34287587	6.592017399	3.11E-18	1.13E-17	30.4814396
OSTM1	-1.394120843	4.895792516	3.20E-18	1.16E-17	30.4507567
AK090844	-1.262111207	6.386378237	3.22E-18	1.17E-17	30.4466611
NFATC2IP	-1.319267403	7.105106665	3.23E-18	1.17E-17	30.4426845
METAP1	-1.276690821	6.361485018	3.26E-18	1.18E-17	30.4333611
USP47	-1.026696	4.531617994	3.37E-18	1.22E-17	30.3996609
RIT1	-1.45324823	5.061947249	3.38E-18	1.22E-17	30.3980854
SPCS3	-1.002667492	6.054027105	3.41E-18	1.23E-17	30.389352
MAP3K7	-1.483919871	5.137720396	3.43E-18	1.24E-17	30.3820809
CXCL1	-1.023415873	5.097037704	3.51E-18	1.27E-17	30.3606942
SUDS3	-1.309541923	4.979336636	3.57E-18	1.29E-17	30.3417672
CAPN7	-1.534317552	3.688904434	3.96E-18	1.43E-17	30.2378259
UHRF2	-1.90523989	7.022555215	4.08E-18	1.47E-17	30.2083611
EIF3C	-1.183562864	3.601446008	4.12E-18	1.48E-17	30.2003871
DPP8	-1.072006643	6.785698442	4.15E-18	1.49E-17	30.1930964
TRMT11	-1.271624036	3.369205391	4.16E-18	1.49E-17	30.189811
CNBP	-1.335240171	7.60214318	4.21E-18	1.51E-17	30.1788651
NOTCH2	-1.093015318	7.423194999	4.34E-18	1.56E-17	30.1466347
CCDC69	-1.457526934	7.464273406	4.41E-18	1.58E-17	30.1308588

C1D	-2.227472773	5.238661588	4.51E-18	1.61E-17	30.1090428
ZMYM1	-1.24524741	3.292890556	4.51E-18	1.62E-17	30.1083073
ZNF644	-1.465844292	3.370040093	4.67E-18	1.67E-17	30.0741711
TSPAN13	-1.578819621	5.278005836	4.79E-18	1.71E-17	30.0482596
RUNX1-IT1	-1.288023732	7.734208344	4.86E-18	1.74E-17	30.0345072
ASF1A	-1.938384457	4.604268937	4.88E-18	1.74E-17	30.0298405
TRIP4	-1.141674631	6.205386832	4.98E-18	1.78E-17	30.0100231
IL32	-1.492566814	8.493269592	5.18E-18	1.85E-17	29.9709343
RAB18	-1.112016264	5.82424628	5.23E-18	1.86E-17	29.9617515
RALA	-1.126077184	5.93001581	5.26E-18	1.87E-17	29.9547384
BNIP3	-1.204353958	4.50009811	5.29E-18	1.88E-17	29.9494087
RRM2B	-2.281471474	4.567717137	5.33E-18	1.90E-17	29.9426893
VIMP	-1.528666239	6.37407129	5.44E-18	1.94E-17	29.9212084
C2orf69	-1.840938055	5.018969636	5.47E-18	1.94E-17	29.9168881
SLC39A10	-1.947981644	4.781137475	5.54E-18	1.97E-17	29.9038946
UBL3	-1.835535282	5.532320124	5.56E-18	1.98E-17	29.8996528
BAG3	-1.424502904	5.964842983	6.11E-18	2.16E-17	29.8052813
DAZAP2	-1.300884074	7.854315438	6.18E-18	2.19E-17	29.7935211
NAAA	-1.017529634	6.976007767	6.20E-18	2.19E-17	29.7908507
MOB4	-1.295142447	5.617582914	6.25E-18	2.21E-17	29.7825252
CTD-2293H3.1	-1.00361267	4.807163759	6.48E-18	2.29E-17	29.7471655
DYRK2	-1.314994892	5.700938444	6.91E-18	2.44E-17	29.6828713
SLC25A24	-1.169387679	4.521112707	7.17E-18	2.53E-17	29.6461771
CSTF2T	-1.088824557	4.920712019	7.18E-18	2.53E-17	29.6449706
NMT2	-1.433299268	5.277008233	7.35E-18	2.59E-17	29.6217
RRM1	-1.108349452	5.412947664	7.42E-18	2.61E-17	29.6110474
TMEM263	-1.892200185	3.931102447	7.70E-18	2.71E-17	29.5746261
TRMT13	-1.595846552	5.385907799	7.86E-18	2.76E-17	29.5541021
TCEAL4	-1.283803994	4.714689252	8.05E-18	2.82E-17	29.5307702
TMEM2	-1.257210661	5.83091331	8.10E-18	2.84E-17	29.5245366
SMA4	-1.362786113	5.650376196	8.13E-18	2.85E-17	29.5205823
RTCA	-1.083675015	4.746036338	8.23E-18	2.88E-17	29.5081817
CEP57	-1.33867789	4.371711855	8.45E-18	2.96E-17	29.4820251
TMEM33	-1.185911529	5.529091663	8.50E-18	2.97E-17	29.4763849
PPIP5K2	-2.414616003	4.419885303	8.55E-18	2.99E-17	29.4696393
ATXN2	-1.072506634	6.338301437	8.72E-18	3.05E-17	29.4498271
RTN3	-1.093981271	8.848324025	8.73E-18	3.05E-17	29.4489338
UFL1	-1.074458048	4.115357433	8.83E-18	3.08E-17	29.4376803
UBP1	-1.911396741	5.996548816	8.90E-18	3.11E-17	29.4294502
MGC24103	-1.163465138	3.131606957	8.92E-18	3.11E-17	29.4282447
MGC12488	-1.179356568	3.308611567	9.25E-18	3.23E-17	29.3911393
MINPP1	-1.297211182	3.311990693	9.39E-18	3.27E-17	29.3764314
FAM198B	-1.370200402	4.532427464	9.60E-18	3.35E-17	29.3538502
PREX1	-1.139066907	8.467620438	1.13E-17	3.92E-17	29.1924327
PAXIP1	-1.266650461	5.121410755	1.13E-17	3.92E-17	29.1903541
AMMECR1L	-1.469525668	5.606158178	1.17E-17	4.04E-17	29.1594893
CD36	-1.023720382	6.141693744	1.18E-17	4.07E-17	29.1523554
STIM2	-1.010313103	5.193612556	1.21E-17	4.20E-17	29.1199531
NCK2	-1.258737666	7.324127388	1.24E-17	4.27E-17	29.1015104
LOC102724356	-1.677715516	7.198308541	1.27E-17	4.38E-17	29.0763091
SMIM3	-1.288947206	6.383384409	1.30E-17	4.47E-17	29.0546271
STARD7	-1.276184743	8.137174955	1.30E-17	4.49E-17	29.0504403
NKAP	-1.018258684	5.015243515	1.33E-17	4.60E-17	29.0253122
ZDHHC6	-1.147550798	6.42557681	1.35E-17	4.65E-17	29.0131105
RAB9A	-1.383520732	5.418656759	1.37E-17	4.71E-17	28.9990506
TMX1	-1.716826166	4.974549562	1.38E-17	4.75E-17	28.9899687
OXR1	-1.023794079	3.923412156	1.40E-17	4.80E-17	28.9788302
ID2	-1.034577262	8.090457181	1.40E-17	4.82E-17	28.9762547

ACOT9	-1.091088343	6.985897347	1.42E-17	4.87E-17	28.9635466
EIF2S3	-1.29512614	7.576168355	1.47E-17	5.04E-17	28.928402
HSP90AB1	-1.295387836	7.524010415	1.62E-17	5.52E-17	28.8346202
ANKRD20A11P	-1.027637984	6.152332005	1.64E-17	5.61E-17	28.8169435
MAST3	-1.039551583	7.23350717	1.66E-17	5.66E-17	28.8074652
CDK17	-1.209063746	4.696320341	1.72E-17	5.86E-17	28.7717399
HSDL2	-1.015711888	5.408532835	1.75E-17	5.97E-17	28.7536606
LOC100996425	1.004744762	5.605626955	1.79E-17	6.08E-17	28.7346659
PNN	-1.19264672	6.054440585	1.86E-17	6.33E-17	28.6937676
RP11-468E2.5	-1.239203219	6.005734766	1.90E-17	6.47E-17	28.6703219
RNF146	-1.5269836	5.14455416	1.94E-17	6.58E-17	28.6536211
INTS2	-1.198447861	3.436341252	1.95E-17	6.62E-17	28.6473305
PCED1B	-1.334870141	7.695006552	2.04E-17	6.93E-17	28.5992475
ZDHHC17	-1.498226318	4.65283623	2.04E-17	6.93E-17	28.5991755
MAP2K4	-1.365943362	5.743231223	2.05E-17	6.94E-17	28.598413
SYPL1	-1.519083975	6.419762631	2.09E-17	7.08E-17	28.5769368
HMGN4	-1.575984871	7.898850376	2.10E-17	7.12E-17	28.5706765
MRPL13	-1.181110518	3.582439252	2.18E-17	7.38E-17	28.5344029
HNRNPA3	-1.476008629	6.918442719	2.35E-17	7.93E-17	28.4618309
DERA	-1.59166819	5.120806324	2.40E-17	8.12E-17	28.4377597
LCK	-1.294202873	8.398636159	2.43E-17	8.21E-17	28.4260888
KIAA0247	-1.516095385	8.201736374	2.49E-17	8.39E-17	28.4021017
ND6	1.11645689	7.750892002	2.65E-17	8.90E-17	28.3408942
TLR4	-1.698099329	5.871510483	2.67E-17	8.96E-17	28.3338298
G3BP2	-1.260857667	5.486147905	3.01E-17	1.01E-16	28.2128818
NAT1	-1.63007085	4.059007696	3.08E-17	1.03E-16	28.1899223
SNAP23	-1.520103709	6.755874355	3.14E-17	1.05E-16	28.1722795
RASA1	-2.04786476	4.838882581	3.14E-17	1.05E-16	28.1692904
CBX1	-1.615644739	6.097798247	3.17E-17	1.06E-16	28.1606911
P2RY10	-1.421666603	5.593698506	3.26E-17	1.09E-16	28.1346057
BRI3BP	-1.957814942	5.969916602	3.27E-17	1.09E-16	28.1294667
CUTC	-1.149756712	5.417612319	3.28E-17	1.09E-16	28.1284317
CELF2	-1.036792827	8.296035694	3.31E-17	1.11E-16	28.1181335
SSR1	-1.216122826	7.086944958	3.38E-17	1.13E-16	28.0959652
SRSF3	-1.108641139	5.611456878	3.50E-17	1.17E-16	28.0630988
TOB1	-2.72008222	5.542093503	3.67E-17	1.22E-16	28.0149446
GOLPH3	-1.884153081	6.891290115	3.75E-17	1.25E-16	27.994727
GNAZ	-1.024835714	6.481916647	3.97E-17	1.32E-16	27.9370803
LENG8	-1.372229489	6.77689757	4.12E-17	1.37E-16	27.8988814
ACBD3	-1.131343288	5.422666266	4.17E-17	1.38E-16	27.8868801
RC3H1	-1.156592007	6.321624233	4.24E-17	1.41E-16	27.8705465
SH2D3C	-1.004479032	6.957563234	4.38E-17	1.45E-16	27.8384615
LOC283357	-1.735539023	4.845737574	4.40E-17	1.46E-16	27.832692
AGA	-1.093906455	4.889240969	4.47E-17	1.48E-16	27.8174867
CLEC12B	-1.115751517	3.101067217	4.50E-17	1.49E-16	27.811525
MRPS35	-1.27119918	6.023791232	4.55E-17	1.50E-16	27.8009265
PSIP1	-1.317913577	5.591648242	4.55E-17	1.50E-16	27.8008524
CYB5R4	-1.288691676	4.742700604	4.56E-17	1.51E-16	27.7975682
SSB	-1.128200909	5.377268047	4.71E-17	1.56E-16	27.7650565
FAM13B	-1.524185843	6.033461844	4.78E-17	1.58E-16	27.7502959
C14orf142	-1.09477864	3.609839437	5.02E-17	1.65E-16	27.7014392
BTG1	-1.013651106	7.796647833	5.03E-17	1.66E-16	27.6995535
STXBP3	-1.320395894	5.705958914	5.09E-17	1.68E-16	27.6875216
LINC00657	-1.331558162	6.215224527	5.18E-17	1.70E-16	27.6702372
CXXC5	-1.031006203	7.236409564	5.23E-17	1.72E-16	27.6609049
TOP2B	-1.583688044	7.457587073	5.24E-17	1.72E-16	27.6587925
SUMO1	-1.039998434	5.869238185	5.35E-17	1.76E-16	27.6382491
ADAM10	-2.026517137	5.317715729	5.48E-17	1.80E-16	27.6144051

KLHL8	-1.082501128	5.642885787	5.50E-17	1.80E-16	27.610136
CBX3	-1.22886959	5.859739117	5.58E-17	1.83E-16	27.5969169
ARL14EP	-1.251036081	5.373067899	5.73E-17	1.88E-16	27.5708855
ICE1	-1.885044156	5.100437199	5.78E-17	1.89E-16	27.5621941
JADE1	-1.024492715	5.79733015	5.84E-17	1.91E-16	27.5517097
MARCKSL1	-1.177776867	8.224230619	6.08E-17	1.99E-16	27.510138
BZW2	-1.018096348	6.854509331	6.41E-17	2.09E-16	27.4575803
SERINC5	-1.727322237	7.088705599	6.43E-17	2.10E-16	27.4551475
CNOT6	-1.439635479	5.331178336	6.43E-17	2.10E-16	27.4551094
PYHIN1	-1.290719224	5.504615012	6.58E-17	2.14E-16	27.4325054
ZZZ3	-1.07256104	4.966266001	6.60E-17	2.15E-16	27.4290295
SENP6	-1.368087235	3.813929169	6.79E-17	2.21E-16	27.4006343
HGS	-1.053994879	6.068070656	7.02E-17	2.29E-16	27.367019
DENND5A	-1.462300725	8.186224955	7.31E-17	2.38E-16	27.3271846
SEC24B	-2.151270357	5.929259255	7.50E-17	2.44E-16	27.301259
MAPRE1	-1.121087105	7.423982395	7.58E-17	2.46E-16	27.2905425
SOAT1	-1.095188356	5.895318127	7.74E-17	2.51E-16	27.2698256
HSPA13	-1.374671368	3.400169816	7.81E-17	2.53E-16	27.2609485
IFIH1	-1.129449083	3.723033365	8.16E-17	2.64E-16	27.2167239
RAF1	-1.096912061	8.194235097	8.35E-17	2.70E-16	27.1936122
RASSF5	-1.308851492	8.283015418	8.40E-17	2.72E-16	27.1882343
BEX5	-1.048722081	4.595466164	8.48E-17	2.74E-16	27.178754
TRAM1	-1.312675062	6.581192506	8.49E-17	2.75E-16	27.1770953
FAIM3	-1.342641382	8.844678248	8.61E-17	2.78E-16	27.163383
DMTF1	-1.9622879	5.819974201	8.80E-17	2.84E-16	27.1421656
EBAG9	-1.241409767	5.205925299	8.83E-17	2.85E-16	27.138114
GBE1	-1.360285267	5.778151885	9.00E-17	2.90E-16	27.1195823
LXN	-1.14760373	5.534153407	9.11E-17	2.94E-16	27.1068496
IL6R	-1.004114341	8.010395247	9.26E-17	2.99E-16	27.0907515
TARDBP	-1.33727537	7.222438639	9.47E-17	3.05E-16	27.0683085
SEC23A	-1.383538087	4.679481309	9.83E-17	3.16E-16	27.0310875
CD247	-1.418986281	9.150064205	9.88E-17	3.18E-16	27.0260949
CCNG2	-1.3679951	5.37493155	1.03E-16	3.32E-16	26.9804567
KLF6	-1.003983828	8.368442625	1.04E-16	3.35E-16	26.9709592
SKIV2L2	-1.054982694	4.875084661	1.05E-16	3.37E-16	26.9656314
NUDT21	-1.345846005	4.199793261	1.09E-16	3.49E-16	26.9294567
RP11-124L9.5	-1.656904396	4.192352232	1.14E-16	3.65E-16	26.8848906
ZNF117	-1.089642664	3.389595105	1.17E-16	3.73E-16	26.860349
GALC	-1.189593496	5.171770093	1.17E-16	3.74E-16	26.8581313
TMEM230	-1.574305059	7.121181714	1.17E-16	3.75E-16	26.8560504
ATP6AP2	-1.064987398	6.849865467	1.23E-16	3.93E-16	26.8071256
CTB-167B5.2	-2.643644897	4.832618833	1.27E-16	4.06E-16	26.7735346
KDM3A	-1.176879144	4.548754979	1.29E-16	4.12E-16	26.7577761
SUMO4	-1.076528158	7.221532619	1.30E-16	4.14E-16	26.7529238
PTGER4	-1.609348503	5.747160665	1.37E-16	4.37E-16	26.6981403
MGME1	-1.475974467	5.953957071	1.40E-16	4.44E-16	26.6811775
KPNA3	-1.23779154	6.471085327	1.42E-16	4.50E-16	26.6663252
SNX6	-1.260579662	7.749107351	1.42E-16	4.51E-16	26.664611
RBM5	-1.061005174	7.454771425	1.45E-16	4.62E-16	26.6409961
YTHDF3	-1.772563071	5.090463512	1.48E-16	4.69E-16	26.625191
RSRC2	-1.089513422	6.938081031	1.50E-16	4.77E-16	26.6078705
DGKA	-1.01242937	7.602478531	1.51E-16	4.79E-16	26.6031127
SGK1	-2.009237783	7.492347686	1.52E-16	4.82E-16	26.5962544
LMBRD1	-1.607172622	6.320745795	1.55E-16	4.92E-16	26.5753683
TBC1D4	-1.759388591	5.201974464	1.56E-16	4.93E-16	26.5719449
CAMK4	-1.147359161	5.150720094	1.57E-16	4.98E-16	26.5629667
LINC01003	-1.789604765	5.799898364	1.57E-16	4.98E-16	26.5620112
TRAC	-1.179575926	9.472329052	1.60E-16	5.07E-16	26.5432713

TMEM14C	-1.802645334	5.827233835	1.68E-16	5.30E-16	26.4980543
TMEM30A	-1.973321938	5.246528981	1.68E-16	5.32E-16	26.4935463
ETS1	-1.100121579	6.119059523	1.74E-16	5.49E-16	26.4617867
MED6	-1.226993004	5.551637835	1.81E-16	5.70E-16	26.4213488
BEX2	-1.009237863	5.688954828	1.84E-16	5.78E-16	26.4066001
CEP192	-1.382677307	4.030660238	1.89E-16	5.93E-16	26.380912
NIPSNAP3A	-2.106955166	3.896508334	1.90E-16	5.99E-16	26.3710821
CREB1	-1.1534687	6.573464397	1.91E-16	5.99E-16	26.3707909
GIMAP6	-1.648926587	7.391412631	1.91E-16	6.01E-16	26.3666917
SIRT1	-1.95674928	5.244542603	1.94E-16	6.11E-16	26.3510193
WDR92	-1.165310149	5.741312536	1.95E-16	6.13E-16	26.3475691
TANK	-1.142310523	5.866302285	2.07E-16	6.48E-16	26.29035
CCDC28A	-1.366053765	7.312651124	2.07E-16	6.50E-16	26.2861942
DSC1	-1.199320201	3.712947991	2.11E-16	6.62E-16	26.2678813
ZBTB1	-1.298697475	3.527739138	2.11E-16	6.62E-16	26.2671751
ORC4	-1.165614923	4.84903641	2.17E-16	6.81E-16	26.2385717
CCR2	-1.620417	6.078603229	2.22E-16	6.94E-16	26.2192375
BC047651	-1.618563714	4.213345584	2.22E-16	6.96E-16	26.216813
BCLAF1	-1.264758073	5.531303723	2.27E-16	7.10E-16	26.1954195
C2orf47	-1.181281053	5.383585807	2.33E-16	7.27E-16	26.1711858
MAT2B	-1.040410486	6.304202423	2.38E-16	7.45E-16	26.1467918
USP14	-1.131214263	6.785169638	2.44E-16	7.61E-16	26.1243453
HMGCR	-1.530439966	4.932351051	2.52E-16	7.84E-16	26.0935672
ITGA4	-2.113414504	4.795231576	2.52E-16	7.85E-16	26.0928338
NRIP1	-1.612986998	5.708047239	2.52E-16	7.85E-16	26.0924436
CARKD	-1.065897748	6.51415753	2.55E-16	7.93E-16	26.080784
HLA-DPB1	-1.056091569	7.354651871	2.56E-16	7.97E-16	26.076724
RAB8B	-1.644526836	6.019369375	2.57E-16	8.00E-16	26.0717167
THEM4	-1.108079094	5.696905083	2.60E-16	8.09E-16	26.0600561
MIB1	-1.363575584	4.43597616	2.62E-16	8.16E-16	26.0513212
TM9SF3	-1.066199452	5.263547276	2.67E-16	8.29E-16	26.0351275
CXCR2	-1.614549782	9.623725078	2.74E-16	8.51E-16	26.0075549
DDX1	-1.167599881	6.414128232	2.82E-16	8.75E-16	25.9789611
RAD23B	-1.310575807	5.540110678	2.85E-16	8.83E-16	25.9702421
ABHD5	-1.253940448	5.841648531	2.89E-16	8.97E-16	25.954148
AKAP11	-1.48559193	5.930921715	2.89E-16	8.97E-16	25.9537496
ZBTB21	-1.384595977	4.622206522	3.18E-16	9.83E-16	25.860768
SMAP1	-1.336481125	7.050171182	3.19E-16	9.85E-16	25.8578977
PRPS2	-1.25712485	4.539935192	3.25E-16	1.01E-15	25.8371983
SLC37A3	-1.383870306	4.833829553	3.33E-16	1.03E-15	25.812106
ZFP36	-1.208169451	9.104167598	3.34E-16	1.03E-15	25.8107633
SESN1	-1.003848501	5.322568782	3.39E-16	1.05E-15	25.7966289
RPA3OS	-1.319452277	3.512890219	3.43E-16	1.06E-15	25.7830264
RHOA	-1.111905661	7.53546146	3.51E-16	1.08E-15	25.7612373
SNX2	-1.188374842	5.076210211	3.55E-16	1.09E-15	25.750277
GPCPD1	-1.323987674	6.256372289	3.58E-16	1.10E-15	25.7400867
RASGRP1	-1.894222372	6.929740953	3.60E-16	1.11E-15	25.7363393
PI4KA	-1.057703222	6.616083074	3.70E-16	1.14E-15	25.7096303
CAPZA2	-1.577231348	5.524947162	3.71E-16	1.14E-15	25.706357
GOLGA4	-1.102206621	6.067973912	3.79E-16	1.17E-15	25.6840116
ANXA5	-1.176907564	8.52572489	3.87E-16	1.19E-15	25.6645817
C6orf120	-1.875214881	4.870228531	3.93E-16	1.21E-15	25.649302
P2RX7	-1.037132676	5.354161676	3.95E-16	1.21E-15	25.6431244
NR3C1	-1.24035705	8.087026713	4.03E-16	1.24E-15	25.6232358
RWDD4	-1.330513008	4.175233522	4.06E-16	1.25E-15	25.6150998
ATRX	-1.390031706	5.73005946	4.14E-16	1.27E-15	25.5959686
FCHO2	-1.245028126	3.594518643	4.21E-16	1.29E-15	25.5785138
KMT2E	-1.391531113	7.42508505	4.37E-16	1.34E-15	25.5424547

TM6SF1	-1.897450184	6.18450635	4.67E-16	1.43E-15	25.476309
AC068039.4	-1.036036348	4.992695688	5.05E-16	1.54E-15	25.3980172
SMC4	-1.021380276	4.01125572	5.48E-16	1.67E-15	25.31591
GHITM	-1.172670874	7.467887173	5.53E-16	1.68E-15	25.3077985
RP11-469M7.1	-1.027038692	4.50652512	5.54E-16	1.68E-15	25.3062821
SMIM15	-2.420996681	4.297087282	5.62E-16	1.71E-15	25.2919587
IRF2BPL	-1.244986014	7.969387631	5.75E-16	1.74E-15	25.2691627
CLNS1A	-1.007841827	6.820371589	5.86E-16	1.78E-15	25.2500243
FGFR1OP2	-1.29889731	5.013977901	5.91E-16	1.79E-15	25.240871
TSPAN2	-1.308797683	4.3803211	6.01E-16	1.82E-15	25.2243776
RMI1	-1.445038046	5.166976675	6.05E-16	1.83E-15	25.2182266
KIAA1279	-1.279170203	4.498959476	6.07E-16	1.84E-15	25.2139221
TOMM20	-1.499194679	7.142968925	6.10E-16	1.85E-15	25.2088296
ZNF146	-1.091714631	4.931386412	6.12E-16	1.86E-15	25.2055779
IER3IP1	-1.007687967	4.524717665	6.19E-16	1.87E-15	25.1946692
LRRC25	-1.019109176	8.286323793	6.48E-16	1.96E-15	25.1486929
DARS	-1.047618302	5.302282544	6.99E-16	2.11E-15	25.0734064
CCNH	-1.226824905	6.024912769	7.50E-16	2.26E-15	25.0037162
MTMR6	-1.336131647	4.129756706	7.53E-16	2.27E-15	24.999095
SDHD	-1.286275177	6.732126207	7.54E-16	2.27E-15	24.9978231
TMEM69	-1.34681473	6.497839405	7.78E-16	2.34E-15	24.9667254
TIMM9	-1.037906199	5.160821239	8.02E-16	2.41E-15	24.9365368
ZYG11B	-1.152118661	6.304007721	8.35E-16	2.51E-15	24.896031
CSF3R	-1.191162498	9.802228565	8.36E-16	2.51E-15	24.8956086
ACBD5	-1.133763984	4.298538661	8.40E-16	2.52E-15	24.8898314
LIPT1	-1.359673135	5.017807354	8.98E-16	2.69E-15	24.8242523
FAM206A	-1.081310015	5.874434971	9.04E-16	2.70E-15	24.817201
GSAP	-2.313831261	5.512456568	9.27E-16	2.77E-15	24.7920733
VIM	-1.147777734	7.796024715	9.44E-16	2.82E-15	24.7737735
AC079767.4	-1.003596407	3.88349483	9.48E-16	2.83E-15	24.7694643
SENP7	-1.140412594	3.019298546	1.00E-15	2.98E-15	24.7149123
TMEM126B	-1.069622181	4.375182084	1.01E-15	3.01E-15	24.7077961
RHOT1	-1.185736351	6.156474956	1.02E-15	3.05E-15	24.6918475
KLF10	-2.118818153	5.717747301	1.03E-15	3.07E-15	24.6858207
PTER	-1.377276354	5.420332362	1.05E-15	3.13E-15	24.6654228
UGP2	-1.054434043	6.643084731	1.06E-15	3.15E-15	24.6600306
RCN2	-1.333679272	4.485586312	1.11E-15	3.28E-15	24.6159014
BTN3A3	-1.238434458	6.380467125	1.15E-15	3.40E-15	24.5798705
ZNF195	-1.29365101	4.114034642	1.21E-15	3.57E-15	24.5301754
MICU2	-2.534034323	5.451823236	1.21E-15	3.57E-15	24.5300186
ERGIC2	-1.165804842	4.923745972	1.25E-15	3.69E-15	24.4962843
SLK	-1.823388629	5.834667558	1.28E-15	3.80E-15	24.468555
SLBP	-1.943871363	6.551125216	1.29E-15	3.82E-15	24.4631312
ACSL3	-1.515609366	4.701909669	1.30E-15	3.86E-15	24.451603
FPR2	-1.74707528	6.788851349	1.31E-15	3.87E-15	24.4483925
LRRC47	-1.217933406	6.77262932	1.37E-15	4.03E-15	24.4053275
SLU7	-1.327250435	5.75648808	1.38E-15	4.06E-15	24.3985563
ABHD3	-2.374610334	6.385651543	1.39E-15	4.09E-15	24.3909046
UBE2G1	-1.476031018	6.269063313	1.40E-15	4.12E-15	24.383388
NUFIP2	-1.137628514	6.828610114	1.51E-15	4.44E-15	24.3073456
GOLGA5	-1.271089673	6.123525954	1.52E-15	4.47E-15	24.2984377
USO1	-1.147042404	6.157022707	1.55E-15	4.54E-15	24.282376
ANKRD27	-1.504753219	5.967990155	1.56E-15	4.58E-15	24.2732532
NDUFAB1	-1.076750134	7.796394619	1.57E-15	4.61E-15	24.265926
C7orf60	-2.143858782	4.222453621	1.57E-15	4.61E-15	24.2651614
COPB1	-1.059896951	7.319612036	1.61E-15	4.73E-15	24.2399751
PABPN1	-1.262837959	7.07106377	1.62E-15	4.74E-15	24.237972
TBC1D10C	-1.073825247	8.255502501	1.67E-15	4.89E-15	24.2047619

EBPL	-1.274589337	6.163733804	1.69E-15	4.95E-15	24.1935507
LCOR	-1.591063428	6.021931654	1.72E-15	5.03E-15	24.1760877
POLR2B	-1.127840481	5.618779908	1.76E-15	5.14E-15	24.1527422
TRG-AS1	-1.323511674	7.637704941	1.87E-15	5.45E-15	24.0935361
PDCD6IP	-1.127585135	6.782867664	1.90E-15	5.55E-15	24.0741482
DDHD2	-1.708108722	4.757695495	1.93E-15	5.61E-15	24.0628344
LINC00597	-1.00034031	3.595549296	1.93E-15	5.62E-15	24.061441
LOC101927451	-1.138024351	6.526853205	1.93E-15	5.63E-15	24.058787
ZNF121	-1.451799998	5.656243299	1.96E-15	5.72E-15	24.0434028
FKBP5	-1.331011507	6.946174436	2.08E-15	6.03E-15	23.9883475
EIF4E3	-1.317845491	5.698276066	2.11E-15	6.14E-15	23.9710182
KANSL2	-1.299422068	5.525538879	2.18E-15	6.33E-15	23.9392422
CCDC109B	-1.132757459	7.289174161	2.24E-15	6.49E-15	23.913852
ITK	-2.163062585	7.811720152	2.34E-15	6.78E-15	23.8689418
C1GALT1	-1.031021317	5.170939347	2.39E-15	6.93E-15	23.8455738
MORC3	-2.131237371	5.15253599	2.41E-15	6.97E-15	23.8406279
CD8A	-1.336474618	7.602163668	2.41E-15	6.99E-15	23.8372602
RP5-1074L1.4	-1.446050088	4.904033661	2.48E-15	7.19E-15	23.8088947
SATB1	-1.362919028	7.024308827	2.56E-15	7.41E-15	23.777337
TEP1	-1.126688697	5.848438699	2.62E-15	7.57E-15	23.7558283
PTGS2	-1.715324497	4.158732789	2.65E-15	7.65E-15	23.745619
IPO7	-1.26910958	6.038483612	2.66E-15	7.69E-15	23.7394303
TMEM106B	-1.132385095	5.442784223	2.67E-15	7.70E-15	23.737884
CRLF3	-1.716354398	8.302157552	2.69E-15	7.76E-15	23.7302415
CES1P1	1.05917341	4.52121293	2.71E-15	7.81E-15	23.7233199
YTHDF2	-1.094362881	7.409322717	2.74E-15	7.92E-15	23.709573
FBXO33	-1.294851424	5.875241761	2.78E-15	8.02E-15	23.6963887
ZFAND1	-1.39301667	3.754880051	2.82E-15	8.13E-15	23.6822169
GPRASP1	-1.341289143	4.991122008	2.88E-15	8.28E-15	23.6627952
ACAP2	-1.161188991	5.545452232	2.90E-15	8.34E-15	23.6553399
SLC20A1	-1.084822174	8.189332835	3.06E-15	8.80E-15	23.5997757
BST1	-1.310793384	5.946400227	3.18E-15	9.13E-15	23.56295
MANBA	-1.016949536	6.379056978	3.22E-15	9.24E-15	23.550241
FAM60A	-1.019769805	5.868309973	3.25E-15	9.32E-15	23.5419935
TBC1D23	-1.457335694	5.589824919	3.41E-15	9.78E-15	23.4925824
H3F3A	1.276118942	6.185309908	3.48E-15	9.96E-15	23.4730965
UPF2	-1.424957231	6.071319038	3.50E-15	1.00E-14	23.4661885
LY75	-2.372412608	6.092216861	3.52E-15	1.01E-14	23.461241
HSD17B7	-1.126529749	6.681719275	3.56E-15	1.02E-14	23.4508096
KIR2DS2	-1.131595587	4.582664506	3.67E-15	1.05E-14	23.4192435
OXCT1	-1.249749546	5.219839244	3.67E-15	1.05E-14	23.4187665
FBXL3	-1.366078953	5.67132278	3.75E-15	1.07E-14	23.3993298
PIGP	-1.062682047	6.173103661	3.83E-15	1.09E-14	23.3776854
LAX1	-1.180697272	6.851701612	3.94E-15	1.12E-14	23.3491329
PIK3R1	-1.256016659	6.5452385	3.96E-15	1.13E-14	23.3433058
THEMIS2	-1.00747353	8.99130059	4.19E-15	1.19E-14	23.2873357
UCHL3	-1.248188756	5.512302946	4.30E-15	1.23E-14	23.2607772
IFRD1	-1.475418563	4.924376979	4.32E-15	1.23E-14	23.2576966
MRFAP1L1	-1.860080847	6.046384489	4.44E-15	1.26E-14	23.2310249
TRIM52	-1.20173645	5.880872069	4.55E-15	1.29E-14	23.2060827
HERPUD2	-1.26438571	7.368222016	4.85E-15	1.38E-14	23.1428011
ARID4A	-1.593262454	4.455970265	4.91E-15	1.39E-14	23.1295912
DPY19L3	-1.743865433	4.751057448	4.95E-15	1.40E-14	23.1211735
EHBP1L1	-1.004037645	6.692575933	4.96E-15	1.41E-14	23.1191143
PCNA	-1.226364716	6.550150306	5.08E-15	1.44E-14	23.0956626
ST8SIA4	-1.472951042	4.787106428	5.18E-15	1.47E-14	23.0761954
RTN4	-1.046392562	8.841261165	5.22E-15	1.48E-14	23.0694538
VRK1	-1.213601245	3.950718931	5.31E-15	1.50E-14	23.0509433

CCNC	-1.068549219	4.113020448	5.33E-15	1.51E-14	23.0477753
DPH3	-1.439149593	5.144352022	5.42E-15	1.53E-14	23.0308471
PRKAR1A	-1.591129906	7.538676719	5.86E-15	1.65E-14	22.9533202
ASNSD1	-1.446302315	5.992319234	6.07E-15	1.71E-14	22.9176931
SEC22B	-1.35615612	7.482679857	6.08E-15	1.71E-14	22.9164126
CCDC91	-1.759956968	4.506822629	6.58E-15	1.85E-14	22.8373326
LYPLA1	-1.876791126	6.554904806	7.54E-15	2.11E-14	22.7018214
SON	-1.118680778	7.734697144	7.67E-15	2.15E-14	22.6853699
LIMK2	-1.006604143	6.889152209	7.82E-15	2.19E-14	22.6653957
FUNDC1	-1.297425394	4.066900887	8.00E-15	2.24E-14	22.6426154
VAMP3	-1.265927212	7.593813588	8.07E-15	2.26E-14	22.634147
BC045784	-1.074591507	3.942940978	8.14E-15	2.27E-14	22.6264169
RANBP6	-1.739623359	3.878762547	8.44E-15	2.35E-14	22.5900342
HNRNPDL	-1.01154545	6.018298506	8.60E-15	2.40E-14	22.5711021
VEZF1	-1.11183922	7.057497721	9.63E-15	2.68E-14	22.4578957
CDC42SE2	-1.67070593	6.624531844	9.98E-15	2.77E-14	22.4230496
UBE2Q2	-2.311157258	5.066039739	1.02E-14	2.83E-14	22.4016341
RP11-97C16.1	-1.502099579	4.730982754	1.05E-14	2.92E-14	22.3696992
KLRG1	-1.274600356	7.285632077	1.06E-14	2.95E-14	22.3600002
CLK4	-1.160349829	4.427054648	1.10E-14	3.04E-14	22.3273333
CCL4	-1.057993359	7.455738004	1.11E-14	3.06E-14	22.3201372
NARS	-1.338350928	8.095422015	1.12E-14	3.09E-14	22.3116037
TRMT61B	-1.319840311	4.522299578	1.13E-14	3.13E-14	22.2990003
IKZF3	-1.037688351	5.716619926	1.14E-14	3.16E-14	22.2858517
SACM1L	-2.372715738	6.639108424	1.17E-14	3.23E-14	22.2658773
MOB1A	-1.543017498	7.310953716	1.19E-14	3.29E-14	22.2449242
WWP1	-1.214530335	5.638437793	1.21E-14	3.34E-14	22.2311949
DNMT1	-1.046564902	7.18077918	1.24E-14	3.41E-14	22.2097832
ABI1	-1.285549042	5.384144038	1.26E-14	3.48E-14	22.1879727
FRAT2	-1.164199911	9.288436799	1.28E-14	3.53E-14	22.1744195
BTBD1	-1.581752588	5.936848576	1.28E-14	3.53E-14	22.1723869
PELI1	-1.504341762	7.865923979	1.32E-14	3.62E-14	22.1472682
C16orf72	-1.516269006	6.323115381	1.37E-14	3.78E-14	22.1039261
SMNDC1	-1.934397638	6.421662248	1.39E-14	3.82E-14	22.0911037
DMXL1	-1.314386001	5.003402339	1.41E-14	3.88E-14	22.0766562
DNAJA2	-1.090748611	5.874664895	1.46E-14	3.99E-14	22.0458289
STK17A	-1.005229326	5.606827219	1.49E-14	4.09E-14	22.0200821
KRCC1	-1.477168872	5.011134515	1.51E-14	4.14E-14	22.0073116
HMGN3	-1.699564447	6.941899552	1.54E-14	4.21E-14	21.9913163
LPIN2	-1.089409023	7.617618556	1.60E-14	4.36E-14	21.9544009
MAP3K1	-1.249657214	6.724560307	1.78E-14	4.85E-14	21.8447567
BZW1	-2.12544427	6.208483096	1.78E-14	4.86E-14	21.8435142
PRPF4B	-1.782107066	5.578297331	1.80E-14	4.89E-14	21.8370642
ATP6V1B2	-1.245997971	9.879335718	1.82E-14	4.96E-14	21.822826
DRAM2	-1.380855145	5.495136184	1.84E-14	5.01E-14	21.8122563
TXK	-2.289382581	5.626031293	1.96E-14	5.32E-14	21.7495486
PPP6C	-1.025177125	7.082257686	2.07E-14	5.61E-14	21.6957177
CUL4A	-1.00459641	6.089197855	2.12E-14	5.75E-14	21.6708985
LOC101060691	-1.281214275	5.116849549	2.14E-14	5.80E-14	21.6619542
CD164	-1.800520345	7.047877568	2.19E-14	5.92E-14	21.6413626
LOC100190986	-1.831955342	7.284603475	2.24E-14	6.04E-14	21.6191837
OSCAR	-1.017653252	6.236365362	2.28E-14	6.15E-14	21.6010824
STX3	-1.047688415	6.607775798	2.37E-14	6.39E-14	21.5621752
WAPAL	-1.056275689	5.157674999	2.37E-14	6.40E-14	21.5607717
POLE3	-1.21521683	6.62826893	2.56E-14	6.89E-14	21.4855729
STARD3NL	-1.471294948	5.757064146	2.61E-14	7.02E-14	21.4648861
HMGN1	-1.294659787	8.539448572	2.70E-14	7.27E-14	21.4300887
NRROS	-1.012608725	6.457436657	2.70E-14	7.27E-14	21.4293283

ANKRD55	-1.127904938	5.547312364	2.79E-14	7.48E-14	21.3999756
MMGT1	-1.116485477	7.005667102	2.80E-14	7.52E-14	21.3952661
SCP2	-1.580421875	6.71201789	2.89E-14	7.76E-14	21.3625114
PDZD8	-1.054121228	3.906290153	2.91E-14	7.81E-14	21.3555411
ENTPD1	-1.099679631	5.83037787	3.05E-14	8.16E-14	21.3109733
TSNAX	-2.213289059	4.786526112	3.22E-14	8.60E-14	21.2560803
RHOBTB1	-1.001705173	3.295003837	3.27E-14	8.74E-14	21.2401418
NPTN	-2.461078185	7.487744029	3.28E-14	8.75E-14	21.2382961
SMAP2	-1.079860225	10.21800127	3.30E-14	8.81E-14	21.2309749
CDKN1B	-1.812966436	7.963191046	3.48E-14	9.29E-14	21.1772466
TOPORS	-1.59583995	6.135538793	3.62E-14	9.62E-14	21.1404333
MS4A1	-1.910311684	6.237170921	3.64E-14	9.70E-14	21.1323874
ERAP2	-1.12607136	4.489466788	3.73E-14	9.92E-14	21.1094487
ARL8B	-1.499109356	6.396428842	3.75E-14	9.97E-14	21.1042942
TIPARP	-1.29844332	6.197084761	3.88E-14	1.03E-13	21.0704341
ITM2B	-1.230364917	11.50894492	3.91E-14	1.04E-13	21.0614014
PPP4R1	-1.514833955	7.768298774	4.05E-14	1.07E-13	21.0262957
CCBL2	-1.202037828	6.502409007	4.12E-14	1.09E-13	21.0106436
XRN2	-1.109806649	6.538095163	4.18E-14	1.11E-13	20.9949339
FYB	-1.047237049	7.627827111	4.35E-14	1.15E-13	20.9554985
LOC101928893	-1.465138523	3.004321452	4.52E-14	1.19E-13	20.9182354
CRNKL1	-1.735675566	5.009727331	4.55E-14	1.20E-13	20.9121993
CNTNAP3	-1.144666393	2.869879872	4.79E-14	1.26E-13	20.8593419
FAR1	-1.693572776	4.997956353	4.88E-14	1.29E-13	20.8408655
MRPL32	-1.151796853	5.565167439	4.88E-14	1.29E-13	20.8408623
ARRDC3	-2.142247002	6.104309778	4.90E-14	1.29E-13	20.8385015
OSBPL9	-1.307983296	7.132150731	4.92E-14	1.29E-13	20.8341397
EPS15	-1.503587554	6.305902602	4.92E-14	1.30E-13	20.8333037
C4BPA	-1.124937338	5.32806559	5.22E-14	1.37E-13	20.7744448
DEGS1	-1.113683389	7.277381022	5.38E-14	1.41E-13	20.7445933
RAB33B	-1.582607008	4.198286095	5.42E-14	1.42E-13	20.7372197
CMC2	-1.109275219	5.671446048	5.92E-14	1.55E-13	20.6486022
FAM118A	-1.149725934	5.423761939	6.07E-14	1.59E-13	20.6243822
TRAPPC10	-1.018382501	7.396603017	6.23E-14	1.63E-13	20.5982616
LEMD3	-1.563827831	6.226840819	6.27E-14	1.64E-13	20.591555
C1QBP	-1.2778636	6.045650204	6.32E-14	1.65E-13	20.5835145
TM9SF2	-1.399268928	8.714610994	6.34E-14	1.66E-13	20.5814356
TTC19	-1.382647588	6.055761414	6.58E-14	1.72E-13	20.5439234
GOLT1B	-1.398498328	4.966164093	6.91E-14	1.80E-13	20.4951628
FASTKD1	-1.326094599	5.106936538	7.09E-14	1.85E-13	20.4693891
TNFAIP8	-1.604573524	6.040404393	7.22E-14	1.88E-13	20.4512141
TMEM87B	-1.258718596	4.882581149	7.28E-14	1.89E-13	20.4431266
DDX26B	-1.344203877	4.793157876	7.35E-14	1.91E-13	20.4342723
HIST2H2BE	-1.282643712	6.423224745	7.73E-14	2.00E-13	20.3835467
PAPD4	-1.221004731	6.563502995	7.78E-14	2.02E-13	20.3767958
NAP1L1	-1.304931512	7.195344763	8.06E-14	2.09E-13	20.3424635
CHUK	-1.480070042	4.694329695	8.17E-14	2.12E-13	20.3281079
UBE4A	-1.520749069	7.38115946	8.23E-14	2.13E-13	20.321794
CLEC5A	-1.081895252	3.951800614	8.26E-14	2.14E-13	20.3178924
CTD-3092A11.2	-1.312879255	4.680087697	8.31E-14	2.15E-13	20.3111385
GPR18	-1.303014974	6.757886188	8.44E-14	2.18E-13	20.2960479
GDI2	-1.299603766	7.897657601	8.45E-14	2.18E-13	20.2953383
RP11-158G18.1	-1.528140852	6.621011124	8.81E-14	2.27E-13	20.2539164
OAT	-1.622739128	6.339882875	8.96E-14	2.31E-13	20.2365036
MAN2A1	-1.435778079	6.119785508	8.97E-14	2.31E-13	20.2358943
ANAPC4	-1.188780675	6.293833121	9.79E-14	2.52E-13	20.1484351
AP3S1	-1.469114919	7.313051762	1.02E-13	2.61E-13	20.1125036
CREBL2	-1.230933868	6.233794068	1.02E-13	2.62E-13	20.1061929

EIF5	-1.041420968	6.72290254	1.13E-13	2.90E-13	20.0036514
GTF2H2B	-1.192116938	5.736697101	1.14E-13	2.92E-13	19.9971255
PRKRIR	-1.766553076	5.847736056	1.15E-13	2.93E-13	19.992044
PTPLB	-1.20162208	5.210561255	1.16E-13	2.96E-13	19.9818199
UHMK1	-1.201423668	6.663207451	1.19E-13	3.05E-13	19.9508721
HIF1A	-1.764645431	8.421727738	1.20E-13	3.07E-13	19.9442127
RCSD1	-1.070508758	8.009124854	1.22E-13	3.11E-13	19.9298944
RALB	-1.045446916	7.936989378	1.23E-13	3.15E-13	19.9176769
15-Sep	-1.633289757	8.296563018	1.23E-13	3.15E-13	19.9176234
YWHAQ	-1.647796104	7.631541155	1.25E-13	3.19E-13	19.9042256
UBE2E1	-1.826392446	6.930278859	1.27E-13	3.23E-13	19.8920254
ZNF101	-1.161456047	6.200556344	1.36E-13	3.47E-13	19.8189605
IFNGR2	-1.008917782	9.04597727	1.37E-13	3.48E-13	19.81584
ANKRD44	-1.03639879	5.814873478	1.42E-13	3.62E-13	19.7759669
AGTPBP1	-1.215696395	5.985814399	1.42E-13	3.62E-13	19.7756306
PCBP1	-1.005325729	9.301336315	1.43E-13	3.64E-13	19.7693811
DTX3L	-1.029798795	8.612002268	1.45E-13	3.68E-13	19.7584165
A2M-AS1	-1.987697282	6.346792502	1.46E-13	3.69E-13	19.7533255
SERTAD2	-1.046819348	7.478458041	1.52E-13	3.87E-13	19.7073633
AP1S2	-1.131384525	7.11518163	1.56E-13	3.95E-13	19.6859156
PPP2R5E	-1.081335928	6.344652466	1.58E-13	4.00E-13	19.6738405
COPS5	-1.04486277	6.085081239	1.58E-13	4.01E-13	19.6707272
ADRB2	-1.176693657	6.88629708	1.58E-13	4.01E-13	19.6697455
AZIN1	-1.692106187	5.942670558	1.59E-13	4.02E-13	19.6673434
SPDYE2	-1.289239988	6.628417486	1.63E-13	4.11E-13	19.6437475
LOC100127972	-1.32998887	6.632135199	1.69E-13	4.27E-13	19.6050156
FOXN2	-1.053217018	5.040861439	1.72E-13	4.34E-13	19.586882
SNORA21	-1.205338634	5.731498486	1.87E-13	4.72E-13	19.5035456
CHRM3-AS2	-1.485616792	4.977991873	1.88E-13	4.73E-13	19.5012431
PAG1	-1.228671792	7.061559629	1.88E-13	4.75E-13	19.4965032
OXNAD1	-1.079373538	6.541913902	2.06E-13	5.18E-13	19.4067439
GPR34	-1.068260277	4.102750558	2.15E-13	5.41E-13	19.3633938
PNMA1	-1.348358512	5.695876102	2.20E-13	5.52E-13	19.3433802
ITGAV	-1.454340434	3.653676354	2.20E-13	5.53E-13	19.34138
STAT1	-1.133388402	7.352666441	2.25E-13	5.65E-13	19.3190372
RBM39	-1.047334765	6.882772007	2.28E-13	5.71E-13	19.3076918
ITGAM	-1.006780307	7.866417626	2.31E-13	5.79E-13	19.2938075
ATP10D	-1.614172121	5.81324972	2.35E-13	5.87E-13	19.2788798
NAIP	-1.106151955	6.675950321	2.37E-13	5.92E-13	19.270226
GPBP1	-1.776753885	5.971523237	2.37E-13	5.93E-13	19.2686799
BOD1L1	-1.052038773	6.162733626	2.44E-13	6.09E-13	19.2407123
PAN3	-1.637060556	7.475503885	2.45E-13	6.13E-13	19.2348996
KLF3	-1.070227274	6.027861366	2.47E-13	6.16E-13	19.2283521
CHMP2B	-1.23757048	5.841114216	2.48E-13	6.19E-13	19.2244203
GNA13	-1.295578043	5.53520946	2.57E-13	6.41E-13	19.1875736
BLNK	-1.654061412	5.523266631	2.62E-13	6.53E-13	19.1684218
WRB	-1.314303432	5.252889847	2.66E-13	6.63E-13	19.1538406
NUP107	-1.28666735	5.495822336	2.67E-13	6.65E-13	19.1507127
HEXB	-1.218887288	8.128322236	2.69E-13	6.70E-13	19.1429515
ZMPSTE24	-1.575848682	6.825123	2.75E-13	6.85E-13	19.1193619
LYRM5	-1.192371519	5.749633897	2.85E-13	7.08E-13	19.0859673
MGC57346	-1.088318285	3.951419477	2.95E-13	7.32E-13	19.0513371
EID1	-1.166693994	5.720340533	2.98E-13	7.41E-13	19.0391727
TRAT1	-1.71040553	5.040726938	3.15E-13	7.80E-13	18.9868706
TM2D3	-1.619140822	6.812620839	3.33E-13	8.25E-13	18.928862
ARGLU1	-1.013439592	5.504113413	3.34E-13	8.27E-13	18.9270659
C16orf54	-1.005879194	9.475166659	3.35E-13	8.29E-13	18.9243999
CX3CR1	-1.072254853	8.816558089	3.49E-13	8.61E-13	18.8848965

FGFBP2	-1.716166771	7.273404907	3.51E-13	8.67E-13	18.8778132
ATP6V1A	-1.45058491	8.118454516	3.57E-13	8.82E-13	18.860219
ADNP	-1.205387433	6.238144692	3.59E-13	8.86E-13	18.8549999
PMS2P5	-2.006626771	5.483866992	3.68E-13	9.06E-13	18.8320574
TBC1D9	-1.021441239	5.285517912	3.72E-13	9.17E-13	18.8197366
H1FX	-1.152031504	6.972655833	3.85E-13	9.47E-13	18.7862558
ZNF652	-1.075326697	7.587660083	3.90E-13	9.58E-13	18.7738181
CXCR4	-1.528437302	9.032381365	4.06E-13	9.97E-13	18.7339054
F2RL1	-1.424470545	5.311118598	4.17E-13	1.02E-12	18.7060272
ESCO1	-1.127397175	4.992780185	4.29E-13	1.05E-12	18.6773451
HHEX	-1.395821717	6.514209219	4.35E-13	1.07E-12	18.6650025
CLINT1	-1.124626996	6.026303268	4.35E-13	1.07E-12	18.6646411
KIAA1468	-1.136075878	5.419334855	4.41E-13	1.08E-12	18.6513693
ANP32E	-1.513915655	4.788650607	4.64E-13	1.14E-12	18.5997406
BACH2	-1.064367201	5.78074336	4.97E-13	1.21E-12	18.5320501
DNAJB14	-2.037458943	5.652206744	5.08E-13	1.24E-12	18.5099242
RNF125	-1.640937708	6.720670245	5.24E-13	1.28E-12	18.4789213
IER5	-1.323398448	6.93998367	5.30E-13	1.29E-12	18.4672802
KDM6B	-1.010925049	7.650080222	5.90E-13	1.43E-12	18.3613389
RNF138	-1.815434386	4.297712594	5.96E-13	1.45E-12	18.3519711
USP9Y	-1.424795906	3.224180602	6.02E-13	1.46E-12	18.3418921
SH3BGRL	-1.155138613	8.627769126	6.02E-13	1.46E-12	18.3406912
TRAPPC6B	-1.316384426	4.796019422	6.17E-13	1.50E-12	18.3169022
MAN1A1	-1.601169171	4.748719045	6.27E-13	1.52E-12	18.3006567
PIGB	-1.176580735	6.191940792	6.44E-13	1.56E-12	18.2746099
SGK223	-1.158203866	7.772997436	6.44E-13	1.56E-12	18.2744273
ZNF266	-1.123212847	7.57262305	6.72E-13	1.63E-12	18.2314834
ZNF514	-1.518768831	5.563105512	6.73E-13	1.63E-12	18.2299801
HDHD2	-1.580336288	5.864618054	7.06E-13	1.71E-12	18.1834446
HPS5	-1.046209099	5.110044268	7.62E-13	1.84E-12	18.1074442
ZNF600	-1.963940164	4.82449212	7.62E-13	1.84E-12	18.1072886
CYP1B1	-1.106434278	5.458336134	7.75E-13	1.87E-12	18.0904147
PARP12	-1.133235804	7.728401242	7.82E-13	1.89E-12	18.0808505
HNRNPU-AS1	-2.13932699	5.59231217	8.13E-13	1.96E-12	18.0422599
RAN	-1.362648965	5.730082997	8.23E-13	1.98E-12	18.0298526
FAM126B	-1.342116598	5.425580443	8.54E-13	2.05E-12	17.9938423
RP11-271C24.3	-1.216422423	6.720499012	8.73E-13	2.10E-12	17.971903
WSB2	-1.338163684	7.380199839	9.46E-13	2.27E-12	17.8914834
DMXL2	-1.265188464	6.398773782	9.60E-13	2.30E-12	17.8777356
LOC158402	-1.316044921	4.72795925	9.62E-13	2.30E-12	17.8755617
TMEM128	-1.345761297	4.292258739	9.74E-13	2.33E-12	17.8626618
RPS6KA3	-1.457314645	6.921373502	9.89E-13	2.37E-12	17.8475819
RAD21	-1.419458423	7.487239475	1.04E-12	2.48E-12	17.7993069
CAB39	-1.451880076	6.398522818	1.07E-12	2.56E-12	17.7686282
CLK1	-2.03573828	4.419049728	1.09E-12	2.61E-12	17.7490733
TRBC1	-1.037421431	10.09427983	1.14E-12	2.71E-12	17.7106232
PDP1	-1.397475548	5.147597204	1.15E-12	2.75E-12	17.6939097
LOC340085	-1.038478523	6.176635612	1.19E-12	2.84E-12	17.6623596
EVL	-1.067961511	8.384953626	1.24E-12	2.95E-12	17.6248026
CCT6A	-1.049808451	5.509190932	1.24E-12	2.96E-12	17.6201984
NRAS	-1.235295752	4.834733683	1.24E-12	2.96E-12	17.6189982
CPNE3	-1.339495982	6.951172411	1.29E-12	3.08E-12	17.5801629
SLC25A46	-1.659189382	4.818963052	1.32E-12	3.13E-12	17.5643196
SCOC	-1.096700903	3.135052582	1.33E-12	3.16E-12	17.5545262
TMEM243	-1.444003831	5.915003807	1.42E-12	3.37E-12	17.4868287
CREBRF	-1.058338787	7.555338884	1.44E-12	3.42E-12	17.4709992
NABP1	-1.500259785	5.128921375	1.47E-12	3.49E-12	17.452417
MTIF2	-1.012838095	5.010471373	1.58E-12	3.73E-12	17.3837242

CEBPB	-1.092424872	10.96909854	1.63E-12	3.86E-12	17.3497228
MMADHC	-1.320285808	7.491017628	1.64E-12	3.87E-12	17.3457109
ADD3	-1.316848755	8.935422956	1.69E-12	3.98E-12	17.3165591
PPP1R15B	-1.759960665	6.467093167	1.77E-12	4.17E-12	17.2678192
NKG7	-1.432984036	9.418611494	1.80E-12	4.23E-12	17.2539275
GOLGA7	-1.089996606	7.606633054	1.87E-12	4.40E-12	17.2143051
RAP2C	-1.732633115	5.473146397	1.91E-12	4.50E-12	17.1917503
PCMTD2	-1.070191312	6.152596552	1.94E-12	4.56E-12	17.1767344
HMGB1	-1.180522151	7.665122444	1.96E-12	4.59E-12	17.1696744
MRPL1	-1.173679179	4.261076097	2.04E-12	4.77E-12	17.1297913
MBNL1	-1.101637885	6.358517333	2.18E-12	5.09E-12	17.0634149
KB-431C1.4	-1.882385128	5.249313173	2.25E-12	5.25E-12	17.0314719
BC043356	-1.178399571	5.26492362	2.25E-12	5.27E-12	17.0291244
PICALM	-1.270129398	6.725453512	2.32E-12	5.41E-12	17.0013131
LAMP2	-1.386532676	7.619797615	2.39E-12	5.56E-12	16.9726407
MDH1	-1.12834163	7.521249849	2.41E-12	5.63E-12	16.9612265
TDG	-1.501468148	5.463484694	2.46E-12	5.74E-12	16.9410239
STX11	-1.268995615	5.982464001	2.48E-12	5.78E-12	16.9329796
DHX15	-1.514013484	7.416614726	2.67E-12	6.21E-12	16.8598809
LYRM1	-1.028128311	7.126098816	2.78E-12	6.46E-12	16.8200675
FOXO6	1.001540617	4.40019831	2.79E-12	6.47E-12	16.8179759
LBR	-2.081133957	7.465080088	2.88E-12	6.68E-12	16.785265
SLC35A1	-1.803107565	5.747705402	2.98E-12	6.89E-12	16.7529836
RPS11	1.51459266	7.568108658	3.03E-12	7.02E-12	16.7350553
KLRF1	-1.705832574	6.378131404	3.11E-12	7.19E-12	16.7095171
UBA3	-2.013237017	6.565111703	3.29E-12	7.60E-12	16.6534442
AX747730	-1.470655935	4.445925169	3.41E-12	7.87E-12	16.6181476
SCYL2	-1.221772674	5.930966194	3.41E-12	7.87E-12	16.6181462
BNIP2	-1.629817793	5.426845852	3.42E-12	7.90E-12	16.6134486
FBXL5	-1.015471744	8.271927992	3.76E-12	8.65E-12	16.5205239
UBR7	-1.12835004	5.580733252	3.79E-12	8.71E-12	16.5126476
ND2	-1.01253687	11.30857506	3.94E-12	9.05E-12	16.4743631
PAX8-AS1	-1.008991873	4.586755254	4.18E-12	9.58E-12	16.4156623
IMPA1	-1.706134329	5.758764529	4.25E-12	9.74E-12	16.3987843
RNF139	-1.542721628	5.921292804	4.46E-12	1.02E-11	16.3502967
PTTG1IP	-1.016889112	8.909527956	4.97E-12	1.13E-11	16.2440114
LUC7L3	-1.003412914	6.589351933	5.17E-12	1.18E-11	16.2045567
GLO1	-1.032875074	7.050447248	5.36E-12	1.22E-11	16.1685284
ARMC1	-1.45227649	6.383606148	5.64E-12	1.28E-11	16.1187324
LILRA5	-1.020165586	5.603918171	5.64E-12	1.28E-11	16.1183061
RASA2	-1.377597753	7.045601672	5.99E-12	1.36E-11	16.0577805
CD69	-1.546789727	3.312455587	6.04E-12	1.37E-11	16.0502893
CD160	-1.130145474	5.839375185	6.07E-12	1.38E-11	16.0449501
SLTM	-1.569990877	6.11418548	6.41E-12	1.45E-11	15.9917253
KIAA0226L	-1.23464859	6.378279886	6.42E-12	1.45E-11	15.98954
RBMXL1	-1.175843154	6.603820415	6.68E-12	1.51E-11	15.9505513
EIF3J	-1.106303302	5.095235658	6.68E-12	1.51E-11	15.9497277
LRRN3	-1.662512523	5.330965545	6.71E-12	1.52E-11	15.9459478
CRIM1	-1.033830731	6.198873865	6.81E-12	1.54E-11	15.93034
ZNF12	-1.535711062	5.105144894	7.13E-12	1.61E-11	15.8849579
CMC1	-1.139535569	4.018092049	7.56E-12	1.70E-11	15.8274224
PPIL3	-1.371325736	5.743054296	7.56E-12	1.71E-11	15.8265483
BTAF1	-1.557367631	5.694896492	8.13E-12	1.83E-11	15.7553086
CYBRD1	-1.002831755	5.514281955	8.19E-12	1.84E-11	15.7481791
NOC3L	-1.440446843	4.854369445	8.51E-12	1.91E-11	15.7101159
EOMES	-1.361772041	6.694537165	8.76E-12	1.97E-11	15.6812388
DOCK11	-1.296116453	6.828651485	9.28E-12	2.08E-11	15.6237438
SLC16A6	-1.038716305	5.627867662	9.70E-12	2.17E-11	15.5801912

GZMB	-1.128199907	8.571820711	1.08E-11	2.41E-11	15.4756562
MRPL15	-1.002048273	5.448564018	1.14E-11	2.55E-11	15.4158337
IVNS1ABP	-1.020731638	5.624562258	1.15E-11	2.55E-11	15.4150475
GYG1	-1.034795993	7.49699768	1.15E-11	2.56E-11	15.4140737
KTN1	-1.040759484	6.165830604	1.16E-11	2.58E-11	15.4031901
TMEM181	-1.415414246	6.707914221	1.16E-11	2.59E-11	15.4017894
1-Mar	-1.05865617	5.202821968	1.17E-11	2.62E-11	15.3901177
DUSP6	-1.456288773	8.537319769	1.23E-11	2.73E-11	15.346304
DCK	-1.978643557	5.235551572	1.24E-11	2.77E-11	15.3336575
CLPX	-1.072216167	5.311269191	1.29E-11	2.86E-11	15.2985829
STT3B	-1.847581483	6.58150442	1.29E-11	2.87E-11	15.2949666
DNAJC3	-1.045359264	6.398044898	1.35E-11	2.99E-11	15.2549632
STXBP5	-1.1294402	4.607894416	1.40E-11	3.10E-11	15.2165442
TXNDC9	-1.050645653	4.283032031	1.42E-11	3.15E-11	15.2013975
SF3B1	-1.023724248	7.920900724	1.50E-11	3.32E-11	15.1476382
RP11-589P10.5	-1.007308324	7.265680857	1.53E-11	3.40E-11	15.124409
C21orf91	-1.211294847	5.656728224	1.66E-11	3.67E-11	15.0450713
EFR3A	-1.617311653	5.744473016	1.77E-11	3.90E-11	14.9840896
PPP1R12A	-1.511348919	6.189997899	1.80E-11	3.97E-11	14.9652314
SARAF	-1.683054117	9.358196828	2.12E-11	4.65E-11	14.8047815
ARHGEF3	-1.541204735	6.190702034	2.20E-11	4.82E-11	14.7686354
GVINP1	-1.22698593	5.979758788	2.25E-11	4.94E-11	14.7438496
GNLY	-1.288684576	9.727261186	2.29E-11	5.02E-11	14.7270932
CYP4F3	-1.459440754	8.197318057	2.36E-11	5.17E-11	14.6962623
AGL	-1.937941268	4.059904483	2.54E-11	5.55E-11	14.6256109
CHD1	-1.059687387	6.922955885	2.61E-11	5.69E-11	14.5989297
CDKN2AIP	-1.179717252	5.328440658	3.02E-11	6.57E-11	14.4530709
NOD2	-1.213708391	6.79713363	3.05E-11	6.64E-11	14.4417751
ACTR3	-1.002070318	8.148940164	3.06E-11	6.66E-11	14.4396434
MAP1LC3B	-1.010375962	8.434039506	3.37E-11	7.31E-11	14.343818
C2CD5	-1.641318529	6.091960146	3.42E-11	7.41E-11	14.3304041
C6orf211	-1.916767469	4.33636534	3.47E-11	7.51E-11	14.3157753
VNN1	-1.240788904	4.528834736	3.58E-11	7.75E-11	14.2846516
CMPK1	-1.204427859	4.970065962	3.90E-11	8.43E-11	14.1986476
SERINC1	-1.77049795	7.554628599	3.93E-11	8.49E-11	14.1917118
HIST1H2BC	-1.155194129	5.551982168	3.93E-11	8.49E-11	14.1916428
CHSY1	-1.350541023	6.398266781	3.98E-11	8.59E-11	14.1796854
ZNF559	-1.324935297	5.626534017	4.15E-11	8.95E-11	14.13676
DLD	-1.113679951	5.866119909	4.65E-11	9.99E-11	14.0253152
LAP3	-1.362910374	7.128978907	4.69E-11	1.01E-10	14.015745
RB1CC1	-1.233205964	5.439871978	4.78E-11	1.03E-10	13.9983171
ZBTB16	-1.023347029	5.816846014	4.83E-11	1.04E-10	13.9873292
TVP23B	-1.152018194	3.040834563	4.85E-11	1.04E-10	13.9825107
FAM35A	-1.58374854	5.699966555	5.06E-11	1.08E-10	13.9420756
GCA	-1.761639433	8.719966272	5.13E-11	1.10E-10	13.9281591
C12orf29	-1.080779563	3.950620886	5.49E-11	1.17E-10	13.8605052
NRBF2	-1.577308176	6.870182602	5.54E-11	1.18E-10	13.8513492
MARCKS	-1.064021998	7.03701376	5.72E-11	1.22E-10	13.8204687
TMEM123	-1.907096719	7.8862232	6.11E-11	1.30E-10	13.7546267
CGGBP1	-1.128580666	6.67069063	6.40E-11	1.36E-10	13.7086239
LYSMD2	-1.252946883	8.443411942	6.42E-11	1.37E-10	13.7059957
SHOC2	-1.495192975	7.291881495	6.44E-11	1.37E-10	13.7028368
DSERG1	-1.057001725	7.266173217	6.46E-11	1.38E-10	13.69922
UGCG	-1.040878838	6.331256466	7.37E-11	1.56E-10	13.5683171
ZNF267	-1.751008369	4.02296632	7.57E-11	1.61E-10	13.5416494
CPD	-1.007049222	5.629070609	8.58E-11	1.81E-10	13.4183482
EXOC1	-1.52472399	6.020031709	8.82E-11	1.86E-10	13.3905073
SLC9A6	-1.344691386	5.787257209	8.87E-11	1.87E-10	13.3854305

SGTB	-1.023688333	4.07097449	9.18E-11	1.94E-10	13.3508052
REEP5	-1.126259549	7.211580326	9.47E-11	1.99E-10	13.3207673
PSMD14	-1.148842751	5.97750821	9.71E-11	2.04E-10	13.2956041
GMFB	-1.313283221	4.936408174	9.90E-11	2.08E-10	13.2763272
EXOSC8	-1.014621426	5.609685352	1.02E-10	2.15E-10	13.2421393
NOP58	-1.33425451	6.496143029	1.07E-10	2.25E-10	13.1966656
SRP9	-2.359679755	7.149366141	1.13E-10	2.37E-10	13.1448211
LOC286052	-1.362713171	5.46465568	1.15E-10	2.42E-10	13.123732
PNPLA8	-1.327302584	5.94780682	1.16E-10	2.43E-10	13.1169728
KIAA1033	-1.396974361	6.479022499	1.20E-10	2.52E-10	13.0835925
ATP2B1	-1.339966004	6.177557576	1.34E-10	2.79E-10	12.9780723
CNEP1R1	-1.326267779	6.483980304	1.41E-10	2.93E-10	12.9295772
TCF15	1.126112528	5.639776207	1.48E-10	3.07E-10	12.8814077
RNF111	-1.469680767	6.397012321	1.59E-10	3.30E-10	12.8087912
GIMAP7	-1.11169943	7.623984751	1.75E-10	3.62E-10	12.7135695
KIAA1324	-1.106993379	5.797703836	1.81E-10	3.75E-10	12.6764613
SERP1	-1.355776299	6.589189527	1.82E-10	3.77E-10	12.6720239
NLRC3	-1.120022348	7.599497084	1.82E-10	3.77E-10	12.67119
CPPED1	-1.006099777	8.858328363	1.89E-10	3.90E-10	12.6379497
CST7	-1.018030277	9.157513484	1.89E-10	3.90E-10	12.6367306
MAP3K7CL	-1.371182759	5.746568597	1.90E-10	3.93E-10	12.6287157
KLHL2	-2.023850246	5.165893765	2.04E-10	4.21E-10	12.5586584
HSD17B11	-1.269702455	8.855788601	2.18E-10	4.48E-10	12.4968958
SH3BGRL2	-1.067530135	6.324761454	2.61E-10	5.35E-10	12.3165012
RTP4	-1.063887729	6.003060633	2.78E-10	5.69E-10	12.2542917
TMCC3	-1.13032381	6.397537958	2.85E-10	5.83E-10	12.229861
DDX21	-1.016966578	7.162554424	2.88E-10	5.89E-10	12.2189598
MYC	-1.150820594	7.009159729	3.06E-10	6.25E-10	12.1576399
HSPE1	-1.033449849	5.132463697	3.14E-10	6.39E-10	12.1346525
CLIC3	-1.314065411	6.127087073	3.16E-10	6.43E-10	12.1288686
LEPROTL1	-1.082966322	7.252502479	3.22E-10	6.55E-10	12.1099601
CD46	-1.624582226	7.062454733	3.34E-10	6.80E-10	12.0715281
ITM2A	-1.073532181	7.628061231	3.36E-10	6.82E-10	12.0675526
CCT2	-1.083461304	6.228097355	3.41E-10	6.92E-10	12.0527531
TGFBR3	-1.12439746	6.579618867	3.49E-10	7.09E-10	12.0285679
HIAT1	-1.642986323	6.568649572	3.77E-10	7.63E-10	11.9536274
IGJ	-1.695327779	5.47069069	3.81E-10	7.71E-10	11.9424186
TMEM170B	-1.620129856	5.322295259	3.81E-10	7.72E-10	11.9416886
NOL11	-1.005683129	6.119929131	3.85E-10	7.79E-10	11.931433
FMR1	-1.782471033	5.74990009	4.13E-10	8.34E-10	11.8626421
GCH1	-1.612394206	7.03993444	4.31E-10	8.69E-10	11.8209736
FBXO6	-1.014745075	5.833776778	4.47E-10	9.01E-10	11.7841662
LIPA	-1.126327398	8.331107747	4.68E-10	9.41E-10	11.7387595
IL13RA1	-1.054122753	7.013589495	5.72E-10	1.14E-09	11.5407926
PTPRC	-1.256546424	8.163306779	5.83E-10	1.17E-09	11.5216472
TMEM71	-1.476555802	7.66953165	6.14E-10	1.23E-09	11.4709996
ZNF700	-1.225071485	4.928956363	6.78E-10	1.35E-09	11.3717416
ZNF281	-1.004060526	6.449915819	7.11E-10	1.41E-09	11.3257635
CSF2RB	-1.427730879	9.716531851	7.15E-10	1.42E-09	11.3199831
SELT	-1.154597691	5.884472726	7.53E-10	1.49E-09	11.2682497
GNAI3	-1.122402481	6.304610755	8.57E-10	1.70E-09	11.1405265
KCTD9	-1.059061743	4.614415687	9.13E-10	1.80E-09	11.0781247
PTGES3	-1.342731647	8.781623702	9.85E-10	1.94E-09	11.003493
PGRMC1	-1.111947921	6.873036002	1.04E-09	2.04E-09	10.9509021
CASP3	-1.165474565	4.912991116	1.09E-09	2.15E-09	10.901465
TAB2	-1.321419839	6.347545779	1.18E-09	2.32E-09	10.8240752
EXOC8	-1.664868336	4.824996156	1.20E-09	2.36E-09	10.8068065
AKR1C3	-1.148433562	4.408240665	1.30E-09	2.54E-09	10.730073

BAZ1A	-1.131671938	7.415849691	1.32E-09	2.59E-09	10.7116788
PPP3CA	-1.118406655	7.506819886	1.44E-09	2.81E-09	10.6278096
NELL2	-1.219181897	7.26328436	1.46E-09	2.85E-09	10.6129831
RASSF3	-1.101296605	7.338431035	1.89E-09	3.67E-09	10.3578352
FAM65B	-1.040805004	9.352689567	2.05E-09	3.96E-09	10.2814756
GALNT7	-1.321949618	5.827830948	2.29E-09	4.42E-09	10.1706586
HSPBAP1	-1.020561218	6.918792418	2.30E-09	4.44E-09	10.1649979
ANP32A-IT1	-1.479478662	5.978369501	2.43E-09	4.68E-09	10.1131758
GIMAP2	-1.425088104	7.470190346	2.56E-09	4.92E-09	10.061535
SAMD9	-1.017192118	5.164870478	2.57E-09	4.95E-09	10.0546112
ZBTB34	-1.193213485	6.506544916	2.60E-09	5.00E-09	10.0460438
OSBPL11	-1.107418816	6.836176728	2.77E-09	5.32E-09	9.9813468
ZRANB2	-1.212974849	6.491624086	2.85E-09	5.46E-09	9.95565781
PARP8	-1.350093109	6.6814381	3.11E-09	5.95E-09	9.86733258
HSP90AA1	-1.318951883	7.942920129	3.36E-09	6.42E-09	9.79045334
BAZ2B	-1.453400164	6.522937481	3.52E-09	6.71E-09	9.7468071
TAF1D	-1.194490958	6.058335451	3.56E-09	6.80E-09	9.7331353
AGAP4	-1.252135966	6.396220762	3.77E-09	7.17E-09	9.67803179
FAS	-1.33060862	5.20636893	3.99E-09	7.58E-09	9.62183047
TCL1A	-1.075800575	6.426902217	4.85E-09	9.17E-09	9.42957764
GBP3	-1.869222854	5.090183276	4.90E-09	9.26E-09	9.41932412
SUCO	-1.333735152	5.038666024	5.03E-09	9.49E-09	9.39402057
PPA1	-1.137185334	7.401168156	5.60E-09	1.05E-08	9.28791067
GBP5	-1.150412705	6.295273202	5.69E-09	1.07E-08	9.27202559
LAMTOR3	-1.2013605	4.848156391	5.71E-09	1.08E-08	9.26798164
ACSL4	-1.0431212	5.627221966	6.91E-09	1.29E-08	9.08095478
KCTD12	-1.249801634	8.191130904	7.66E-09	1.43E-08	8.97910366
MFSD1	-1.272996027	7.874544986	7.96E-09	1.48E-08	8.94093171
SMCHD1	-1.159016995	7.072621811	8.85E-09	1.65E-08	8.83626684
DYNLT3	-1.11302462	5.695669013	9.48E-09	1.76E-08	8.76914418
RP11-549J18.1	-1.655242601	3.044979223	9.75E-09	1.81E-08	8.74138431
PPT1	-1.018718339	10.02577569	9.94E-09	1.84E-08	8.72201891
GSKIP	-1.661262052	5.132072377	1.07E-08	1.97E-08	8.65415031
CDC42-IT1	-1.154304963	7.535716754	1.14E-08	2.10E-08	8.59012016
DDX60	-1.160850056	4.239929416	1.25E-08	2.30E-08	8.49790885
TDP2	-1.69655743	6.781432968	1.28E-08	2.34E-08	8.4761913
HIST1H2AC	-1.194927095	9.375639236	1.42E-08	2.59E-08	8.37433552
TRIM22	-1.416003728	8.820347628	1.44E-08	2.63E-08	8.36034255
FAM96A	-1.079018117	6.937483964	1.49E-08	2.73E-08	8.32171279
MME	-1.08971891	7.80920771	1.60E-08	2.92E-08	8.25223845
POMZP3	-1.097698313	5.086484955	1.78E-08	3.24E-08	8.14608738
SPOPL	-1.233149636	6.181738127	1.82E-08	3.30E-08	8.12746905
PHOSPHO1	-1.023963676	7.214504756	2.17E-08	3.94E-08	7.95146471
ANKRD49	-1.106131494	6.379908034	2.35E-08	4.24E-08	7.87578992
RRN3P2	-1.004429445	5.90403788	2.35E-08	4.24E-08	7.87519561
QPCT	-1.097762183	8.454175985	2.84E-08	5.10E-08	7.6903606
FAM174A	-1.190784426	6.072481089	2.86E-08	5.13E-08	7.68334044
SLC38A2	-1.651795757	7.306136613	3.03E-08	5.45E-08	7.62374446
CTB-181H17.1	-1.10885136	5.276657044	3.39E-08	6.07E-08	7.51430688
DENND6A	-1.188597146	6.028578652	3.61E-08	6.44E-08	7.4539611
ZNHIT3	-1.106068974	4.884085371	3.72E-08	6.64E-08	7.42371055
CCNG1	-1.538427643	6.735742621	3.73E-08	6.65E-08	7.4216057
LOC100505874	-1.278987349	3.973024939	4.01E-08	7.14E-08	7.35078999
GPR183	-1.036161969	6.771539192	4.32E-08	7.69E-08	7.27606525
TLR8	-1.098492227	7.47806195	4.60E-08	8.18E-08	7.21404367
TLR1	-1.191461296	7.893875735	4.98E-08	8.84E-08	7.13589764
RBM38	1.106418137	9.832165374	5.05E-08	8.95E-08	7.12290001
USP1	-1.159988963	6.600678079	5.07E-08	8.98E-08	7.11917629

GOLGA8N	-1.242466965	6.907545803	5.56E-08	9.82E-08	7.0293638
SNORD89	-1.145490453	7.604525805	6.54E-08	1.15E-07	6.86867237
NAE1	-1.070754902	5.929742904	6.67E-08	1.17E-07	6.85053956
PSMC6	-1.496994012	5.459629028	6.91E-08	1.21E-07	6.81573668
SDCBP	-1.044566136	11.23681924	6.98E-08	1.23E-07	6.80481694
PPP1CC	-1.188152157	8.448718091	7.13E-08	1.25E-07	6.78389187
CCR7	-1.011373773	8.423265349	7.76E-08	1.36E-07	6.70133763
DHX29	-1.057352369	5.894689904	7.88E-08	1.38E-07	6.68630121
VBP1	-1.471897701	6.312706868	7.99E-08	1.40E-07	6.67282474
SDPR	-1.053116129	5.730412701	8.02E-08	1.40E-07	6.66869318
YPEL5	-1.038625081	8.242507976	8.04E-08	1.41E-07	6.66694741
VAMP7	-1.095648362	7.083406203	8.33E-08	1.46E-07	6.63160056
MYL4	1.076200549	9.364609791	8.76E-08	1.53E-07	6.5818314
MYOM2	-1.218422546	5.760626931	9.20E-08	1.60E-07	6.53464946
NPTN-IT1	-1.174271449	6.173458728	9.44E-08	1.64E-07	6.50930368
DPM1	-1.177774704	6.323234435	9.89E-08	1.72E-07	6.46352367
CTBS	-1.035771613	6.747803955	1.06E-07	1.84E-07	6.39555765
TUBB1	-1.196891871	7.728955455	1.23E-07	2.13E-07	6.24877017
ITLN1	1.096279857	5.403979104	1.64E-07	2.81E-07	5.96830802
SIRPB2	-1.081867287	7.887734443	1.69E-07	2.91E-07	5.9354157
CSGALNACT1	-1.385032503	5.394884331	1.76E-07	3.02E-07	5.8955258
OLIG1	-1.056288896	6.898510876	1.81E-07	3.10E-07	5.87056163
SNRNP200	-1.109260296	6.430192948	2.27E-07	3.86E-07	5.6506945
CCZ1B	-1.053089837	6.378258472	2.28E-07	3.88E-07	5.64484066
WLS	-1.051520795	5.989551475	2.73E-07	4.63E-07	5.46785892
DEK	-1.587704361	6.720268076	2.91E-07	4.93E-07	5.40465932
HLA-DRB4	-1.795255905	5.139862567	3.13E-07	5.29E-07	5.33385064
SOD2	1.02046075	7.973189867	3.46E-07	5.82E-07	5.23752384
RNF149	-1.100301647	9.948199741	3.50E-07	5.89E-07	5.22516623
ZNF23	-1.037636612	3.931086023	3.67E-07	6.18E-07	5.1780318
COMMD8	-1.460855949	5.020333859	4.06E-07	6.80E-07	5.08091357
GPR65	-1.275430055	6.501257143	4.21E-07	7.05E-07	5.04495455
RAP1B	-1.330387274	9.034234211	4.41E-07	7.39E-07	4.99817561
NFXL1	-1.183603819	4.034685781	5.78E-07	9.61E-07	4.73381316
GPR160	-1.239473246	6.079211357	5.86E-07	9.73E-07	4.72124033
ADAT1	-1.001952694	6.198603596	6.40E-07	1.06E-06	4.63430339
RPS24	-1.145069477	7.079650963	6.62E-07	1.10E-06	4.60126821
TMEM167A	-1.073519179	6.322816599	7.02E-07	1.16E-06	4.54467282
RP11-68I3.11	-1.116380056	5.524133088	7.50E-07	1.24E-06	4.47948759
RNF13	-1.145710829	7.95210324	7.73E-07	1.27E-06	4.45088127
AHSP	1.50707113	10.20486513	8.73E-07	1.43E-06	4.33183453
P2RY14	-1.37411889	5.593410215	1.12E-06	1.83E-06	4.08751283
VPS4B	-1.1677501	7.24380994	1.39E-06	2.26E-06	3.87704461
RGS18	-1.818720034	6.683566623	1.61E-06	2.60E-06	3.73726382
FGL2	-1.055771971	9.329328408	1.62E-06	2.62E-06	3.72681979
HAT1	-1.187488977	4.407886285	2.04E-06	3.28E-06	3.50398671
ACSL1	-1.124292307	9.09551315	2.67E-06	4.25E-06	3.2428208
BC044596	-1.230488494	6.603716	2.71E-06	4.32E-06	3.22659258
GNG10	-1.286780562	8.897307801	2.84E-06	4.53E-06	3.18054567
FLJ12120	-1.318041428	6.284578223	5.16E-06	8.07E-06	2.60112584
TAF7	-1.122041276	7.642654838	5.32E-06	8.31E-06	2.57209369
FAM8A1	-1.106952788	7.725285015	6.10E-06	9.48E-06	2.43983556
GZMH	-1.227533189	8.347079698	6.25E-06	9.72E-06	2.41554371
SNX10	-1.31845132	6.931322294	8.17E-06	1.26E-05	2.1559972
LYZ	-1.175487076	10.74142117	8.47E-06	1.31E-05	2.1206327
TXLNGY	-1.08951745	4.205630063	1.26E-05	1.92E-05	1.73802986
NAMPT	-1.016192931	9.327638111	1.45E-05	2.21E-05	1.59833397
PPP1CB	-1.132672785	6.592561472	1.69E-05	2.55E-05	1.45526907

LOC102724387	1.064825441	4.091686697	1.89E-05	2.85E-05	1.34617552
IFI44	-1.085270635	4.585670928	2.69E-05	4.01E-05	1.00403372
NEK7	-1.297920098	6.837496273	4.40E-05	6.48E-05	0.52979271
PRKAR2B	-1.10816076	7.374646353	5.79E-05	8.45E-05	0.26627083
RGS2	-1.017180028	10.73228077	6.10E-05	8.90E-05	0.21585124
ABCB10	-1.163482289	4.599812551	8.29E-05	0.000119779	-0.0774897
PF4V1	-1.161472378	6.392290154	0.000111256	0.000159448	-0.3595206
LRRK2	-1.066401911	7.464303274	0.000192494	0.000271808	-0.8829375
KRT1	1.243475499	9.436360118	0.000346464	0.000481312	-1.4417174
CMPK2	-1.0635043	6.596959522	0.000530235	0.000727093	-1.8445994
KDM5D	-1.081175605	5.800888392	0.001952627	0.002580662	-3.067889
APOBEC3B	-1.037409123	3.784904302	0.025806494	0.031265504	-5.4128863

Degree data						
name	Degree	Edge Count	Indegree	Neighborhood Connectivity	Number of Directed Edges	Topological Coefficient
ACVR1C	1	1	0	2	1	0
ACVR2A	2	2	1	1.5	2	0.5
TGFBR3	2	2	2	3	2	0.5
ADRB2	7	7	0	8	7	0.27093596
PTGER2	4	4	2	4.75	4	0.67857143
EGF	12	12	3	19.66666667	12	0.28381643
MC4R	4	4	1	4.75	4	0.67857143
PTGER4	4	4	3	4.75	4	0.67857143
VIPR1	4	4	4	4.75	4	0.67857143
CCL4	14	14	1	10.5	14	0.1779661
CXCR4	14	14	7	15.21428571	14	0.2237395
APC	9	9	0	6.77777778	9	0.28502415
PSMD7	8	8	5	12.75	8	0.27717391
PSME3	8	8	6	12.75	8	0.27717391
CACYBP	1	1	1	9	1	0
PSMC6	8	8	2	12.75	8	0.27717391
PSMD14	8	8	4	12.75	8	0.27717391
HDAC1	7	7	5	5.14285714	7	0.21118012
PSMD11	8	8	3	12.75	8	0.27717391
CASP3	9	9	1	8.77777778	9	0.16326531
TCF7L2	4	4	4	6.25	4	0.31578947
ATF1	2	2	0	7.5	2	0.625
ATF2	9	9	1	9.11111111	9	0.19806763
CREB1	6	6	2	14.83333333	6	0.29333333
NR3C1	12	12	6	15.58333333	12	0.19479167
IRF1	9	9	4	11.33333333	9	0.18888889
EED	2	2	1	8	2	0.61538462
HMGB1	10	10	2	13	10	0.21666667
RELA	26	26	24	11.61538462	26	0.13994439
FOS	8	8	3	12	8	0.21428571
BCL10	5	5	0	15.8	5	0.36744186
PRKCQ	15	15	14	14.53333333	15	0.2127451
MALT1	5	5	3	15.8	5	0.36744186
CHUK	16	16	1	8.875	16	0.17401961
IKBKB	17	17	7	8.58823529	17	0.17058824
BLNK	11	11	0	18.09090909	11	0.36181818
PIK3R1	30	30	25	13	30	0.15116279
VAV3	11	11	11	18.09090909	11	0.31578947
PIK3CA	36	36	27	12.83333333	36	0.14750958
CBLB	11	11	1	17.09090909	11	0.35416667
FGR	9	9	4	17.33333333	9	0.36111111
FYN	28	28	10	14.57142857	28	0.18214286
LCK	31	31	16	14.35483871	31	0.16312317
LAT	13	13	8	16.30769231	13	0.28609987
CD19	6	6	2	16.33333333	6	0.38492063
ZAP70	15	15	15	14.13333333	15	0.27712418
NCK1	9	9	6	16.88888889	9	0.3015873
BRAF	6	6	0	13.33333333	6	0.33760684
CSK	18	18	5	15.94444444	18	0.26574074
RAF1	11	11	9	18.63636364	11	0.24521531
NGF	8	8	4	19.875	8	0.29477612
PAK2	17	17	10	11.82352941	17	0.19607843
NRAS	16	16	9	18	16	0.2278481
MAP2K1	10	10	5	17.3	10	0.31272727

GZMB	4	4	3	4.75	4	0.28333333
TNFRSF1(	5	5	4	9.6	5	0.368
SLK	1	1	1	9	1	0
VIM	1	1	1	9	1	0
RHOA	19	19	14	15.10526316	19	0.19365722
UBR1	1	1	1	11	1	0
PIK3CG	9	9	6	19.44444444	9	0.32956685
CCR7	8	8	1	11.25	8	0.41666667
CXCL5	8	8	4	11.25	8	0.41666667
S1PR1	14	14	14	16.92857143	14	0.27751756
CXCR2	8	8	5	11.25	8	0.41666667
CX3CR1	8	8	2	11.25	8	0.41666667
CXCL1	9	9	3	10	9	0.38034188
TNF	18	18	17	9.61111111	18	0.14478114
FPR2	15	15	9	12.6	15	0.23333333
CD81	1	1	1	6	1	0
CD1D	1	1	0	14	1	0
CD3E	14	14	3	15.64285714	14	0.27318296
CD247	12	12	0	17.5	12	0.32407407
CD28	13	13	1	18.07692308	13	0.296343
CD8B	11	11	4	18.72727273	11	0.34680135
SH3BP2	5	5	3	16.6	5	0.48823529
CD8A	11	11	3	18.72727273	11	0.34680135
ITGAV	12	12	6	17.91666667	12	0.26348039
ITGAL	3	3	2	13.33333333	3	0.44444444
HLA-DPB	10	10	2	12	10	0.25319149
CDK4	3	3	0	9.66666667	3	0.44444444
HSP90AA	15	15	3	14.46666667	15	0.2159204
HSP90AB	7	7	3	11.42857143	7	0.2484472
IFIH1	5	5	1	15.6	5	0.35348837
NOD2	4	4	3	10.5	4	0.58333333
FAS	5	5	1	20.8	5	0.4
TLR1	3	3	3	14.33333333	3	0.65151515
CYLD	5	5	1	14.2	5	0.355
TLR4	7	7	6	15.57142857	7	0.31142857
TNFAIP3	4	4	4	13.75	4	0.47413793
CSF2RB	5	5	0	23	5	0.38965517
STAT1	16	16	15	9.1875	16	0.13257576
ZYX	6	6	6	18.33333333	6	0.39492754
LYZ	1	1	1	9	1	0
F2R	5	5	0	18.2	5	0.40444444
F2RL1	4	4	1	21.5	4	0.48863636
NCK2	5	5	4	17.4	5	0.39090909
GNLY	2	2	0	3	2	0.75
PRF1	2	2	2	3	2	0.75
RXRB	1	1	1	7	1	0
TRIM22	2	2	2	9.5	2	0.59375
TRIM5	2	2	2	9.5	2	0.59375
LGMN	1	1	1	10	1	0
HSPA4	2	2	2	11	2	0.73333333
NR3C2	3	3	3	11.33333333	3	0.53968254
TANK	3	3	1	10	3	0.48333333
IFNGR2	1	1	0	16	1	0
IL10RA	1	1	0	16	1	0
IL12RB2	2	2	0	23.5	2	0.55952381
IL13RA1	1	1	0	16	1	0
IL15	1	1	0	4	1	0
IL18	4	4	1	11.75	4	0.359375

IL18R1	2	2	1	15	2	0.55555556
IL1RAP	3	3	0	30.66666667	3	0.55757576
IL21R	1	1	0	16	1	0
IL6R	2	2	0	13	2	0.52173913
TGFBR1	4	4	3	8.5	4	0.34782609
LIMS1	2	2	0	5.5	2	0.5
NLK	1	1	0	4	1	0
NR2C2	1	1	0	4	1	0
PPP3CA	1	1	0	1	1	0
PPP3CB	1	1	1	1	1	0
PTGS2	1	1	0	18	1	0
RFX5	1	1	0	1	1	0
RFXAP	1	1	1	1	1	0
TNFRSF1(	1	1	1	5	1	0
TXK	1	1	0	11	1	0

Transcription factor					
TF ID	Count	P. Value	Benjamini	FDR	Genes
POU6F1	83	4.35E-04	0.041277	0.037055	SCYL3, ATF2, VIPR1, GMFB, LEAP2, CBLB, HMGB1, IL1RAP, NR3C1, PIK3CG, BACH2, NR3C2, IFIH1, IKBKB, PPP3CA, PPP3CB, SLK, NAMPT, BLNK, NCK2, ITGAV, FAM3C, TRIM22, IL13RA1, VAV3, MAP2K1, MME, CHUK, IL15, SPINK5, FOS, NGF, DCK, TGFBR1, TGFBR3, FGR, PIK3CA, RFX5, PRKCQ, RAF1, TLR4, PSMD11, PSMD14, TXK, IREB2, CXCR4, UBR1, CD1D, PIK3R1, PTGS2, NLK, RLF, TANK, RXRB, NRAS, ACVR1C, PDGFD, S1PR1, FYN, PAK2, TCF7L2, ERAP2, CMTM4, KLRC3, STAT1, IL10RA, ERAP1, CRIM1, SH2D1B, BRAF, ACVR2A, AIMP1, CYLD, PSMC6, APC, TNFSF4, CD28, ZYX, VIM, CD247, CRLF3, SMAP2, IL18R1
IRF7	87	6.07E-04	0.041277	0.037055	ATF2, GMFB, HSP90AB1, CD81, CBLB, IL1RAP, NR3C1, PIK3CG, BACH2, CXCL5, NR3C2, IFIH1, PPP3CA, PPP3CB, SLK, NAMPT, NCK2, ITGAV, SCYL2, PF4V1, NCK1, VAV3, MAP2K1, MME, IL15, EED, LANCL1, F2R, SPINK5, IL18, SYTL1, FOS, TMEM173, RHOA, TAPBP, TGFBR3, FGR, PIK3CA, CD8A, IRF1, PSME3, RFX5, PRKCQ, PIGF, PSMD14, PTGER2, TXK, IREB2, CXCR4, CSF2RB, UBR1, NOD2, NR2C2, NLK, TANK, RXRB, PGRMC2, ACVR1C, CD19, PDGFD, CCL4, S1PR1, CSK, FYN, ICOS, IL12RB2, MICB, TCF7L2, HLA-DRB4, EGF, CMTM4, HSPA4, STAT1, IL10RA, ERAP1, CRIM1, ACVR2A, AIMP1, CYLD, APC, CD28, VIM, CD247, SMAP2, LAT, IL18R1, LIMS1
FOXD3	69	7.04E-04	0.041277	0.037055	SCYL3, ATF2, CBLB, HMGB1, IL1RAP, NR3C1, PIK3CG, BACH2, CXCL5, NR3C2, IFIH1, MC4R, PPP3CA, PPP3CB, SLK, GNLY, CASP3, NAMPT, BLNK, TNFRSF8, FAM3C, IL6R, IL13RA1, VAV3, MME, CHUK, IL15, IL18, NGF, TGFBR1, TAPBP, TGFBR3, FGR, CREB1, NOV, LAP3, CX3CR1, PSMD14, PTGER2, IREB2, CXCR4, UBR1, CD1D, ADRB2, PIK3R1, NR2C2, NLK, TANK, MALT1, ACVR1C, S1PR1, FYN, IL12RB2, TCF7L2, ERAP2, EGF, STAT1, ERAP1, CRIM1, BRAF, ACVR2A, AIMP1, CYLD, PSMC6, APC, CDK4, CD28, CD247, SMAP2

ISRE	84	9.50E-04	0.0418	0.037525	CSF3R, GMFB, HSP90AB1, LEAP2, SECTM1, CBLB, HMGB1, IL1RAP, NR3C1, PIK3CG, BACH2, CXCL5, NR3C2, PPP3CA, PPP3CB, PSMD7, FCGRT, CASP3, NAMPT, BLNK, NCK2, CCR7, ITGAV, FAM3C, CCR6, TRIM27, IL6R, PF4V1, IL13RA1, NCK1, VAV3, MAP2K1, MME, IL15, EED, LANCL1, SPINK5, RHOA, TAPBP, TGFBR3, FGR, CREB1, CD8B, PIK3CA, NOV, CD8A, IRF1, PSME3, RFX5, PRKCQ, PIGF, TLR4, PSMD11, PSMD14, TXK, CXCR4, CSF2RB, ADRB2, NOD2, TANK, MALT1, RXRB, NRAS, ACVR1C, PDGFD, SH3BP2, S1PR1, FYN, IL12RB2, TCF7L2, EGF, CMTM4, HSPA4, IL10RA, ERAP1, CRIM1, SH2D1B, ACVR2A, CYLD, PSMC6, APC, RFXAP, SMAP2, IL18R1
